# Genome-wide survey and phylogeny of S-Ribosylhomocysteinase (LuxS) enzyme in bacterial genomes

**DOI:** 10.1186/s12864-016-3002-x

**Published:** 2016-09-20

**Authors:** Rajas M. Rao, Shaik Naseer Pasha, Ramanathan Sowdhamini

**Affiliations:** 1 National Centre for Biological Sciences, Tata Institute of Fundamental Research, GKVK campus, Bellary Road, Bangalore, 560065 India; 2 Division of Biological Sciences, School of Natural Sciences, Bangalore University, Bangalore, 560056 India

**Keywords:** Genome wide survey, Phylogeny, Homology modelling, LuxS protein

## Abstract

**Background:**

The study of survival and communication of pathogenic bacteria is important to combat diseases caused by such micro-organisms. Bacterial cells communicate with each other using a density-dependent cell-cell communication process called Quorum Sensing (QS). LuxS protein is an important member of interspecies quorum-sensing system, involved in the biosynthesis of Autoinducer-2 (AI-2), and has been identified as a drug target. Despite the above mentioned significance, their evolution has not been fully studied, particularly from a structural perspective.

**Results:**

Search for LuxS in the non-redundant database of protein sequences yielded 3106 sequences. Phylogenetic analysis of these sequences revealed grouping of sequences into five distinct clusters belonging to different phyla and according to their habitat. A majority of the neighbouring genes of LuxS have been found to be hypothetical proteins. However, gene synteny analyses in different bacterial genomes reveal the presence of few interesting gene neighbours. Moreover, LuxS gene was found to be a component of an operon in only six out of 36 genomes. Analysis of conserved motifs in representative LuxS sequences of different clusters revealed the presence of conserved motifs common to sequences of all the clusters as well as motifs unique to each cluster. Homology modelling of LuxS protein sequences of each cluster revealed few structural features unique to protein of each cluster. Analyses of surface electrostatic potentials of the homology models of each cluster showed the interactions that are common to all the clusters, as well as cluster-specific potentials and therefore interacting partners, which may be unique to each cluster.

**Conclusions:**

LuxS protein evolved early during the course of bacterial evolution, but has diverged into five subtypes. Analysis of sequence motifs and homology models of representative members reveal cluster-specific structural properties of LuxS. Further, it is also shown that LuxS protein may be involved in various protein-protein or protein-RNA interactions, which may regulate the activity of LuxS proteins in bacteria.

**Electronic supplementary material:**

The online version of this article (doi:10.1186/s12864-016-3002-x) contains supplementary material, which is available to authorized users.

## Background

Communication between individuals is a critical factor that decides the survival of a population. It is a pivotal factor for the survival of pathogenic and non-pathogenic bacteria alike, the diseases caused by the former being a major health concern, particularly in developing countries. The above objective is achieved in bacteria by means of a cell-to-cell communication process, involving chemical signals called Quorum-Sensing (QS). The process of QS involves the bacterial cell producing chemical signals known as Autoinducers (AIs) [1], which are secreted into the extracellular space.

Till date, three types of autoinducers have been characterised in bacteria: AI-1, AI-2 and AI-3. AI-2 is involved in inter-species cell-cell communication [2], and it was found to be a furanosyl-borate diester, making it the only boron-containing biomolecule characterised till date [3]. Even though AI-2 is observed to contain this element, its presence is highly dependent on the growth conditions of bacteria.

Biosynthesis of AI-2 involves a three-step reaction, which is part of a methionine catabolism cycle, known as Activated Methyl Cycle (AMC). First step involves removal of methyl group from S-Adenosyl Methionine (SAM), which is catalysed by SAM-dependent methyltransferases. Resulting product, S-Adenosyl Homocysteine (SAH), is converted to S-Ribosyl Homocysteine (SRH) by the enzyme SAH Nucleosidase [4]. SRH, in turn, is hydrolysed to 4,5-dihydroxy-2,3-pentanedione (4,5-DPD) by the enzyme S-Ribosylhomocysteinase, also referred to as LuxS protein [5]. 4,5-DPD further undergoes hydrolysis autocatalytically to form AI-2 [2].

An important enzyme involved in AI-2 biosynthesis is S-Ribosylhomocysteinase, also referred to as LuxS protein. This enzyme belongs to LuxS/MPP-like metallohydrolase superfamily according to SCOP system of protein classification. A remarkable feature of this protein is that it is one of the few enzymes capable of cleaving thioether bonds without using a redox cofactor [6]. Moreover, studies on LuxS gene in *E. coli, V. cholerae and S. typhi* have shown that the gene is highly conserved in different species, but do not share any homology with other gene [7].

Numerous structural studies have been performed on LuxS protein. The first attempts to obtain a crystal structure of LuxS protein [8, 9] showed the LuxS protein was a homodimer, retaining eight stranded β-barrel surrounded by six alpha-helices. The active site consists of a zinc-ion, coordinated by residues His54, His58 and Cys126, which are all highly conserved. It was also observed that access to the active site seems to be restricted and is triggered by conformational changes in the protein, involving residues 125–131 and the residues around N-terminus.

Previous studies on evolution of LuxS protein showed that LuxS had evolved early during the divergence of major prokaryotic phyla, based on its broad consensus with single subunit ribosomal RNA tree of bacteria. However, it was inferred that there were instances of horizontal and lateral gene transfer [10]. Another genome-wide survey on LuxS genes in various bacterial genomes have shown that LuxS gene is widespread across the bacterial domain, and AI-2 mediated signalling may indeed be interspecies universal mode of cell-cell communication system [11]. In contrary to these reports, in a study that examined the AI-2 binding receptors, the authors suggested that AI-2 mediated QS is restricted to certain members of Vibrionales, and to some members of pathogenic gut bacteria, and that the role of LuxS protein is limited to AMC [12].

However, with the advent of large-scale bacterial genome sequencing projects, and increasing recognition of the role of LuxS in growth and virulence of various bacterial pathogens, a broader perspective is required on the evolution of QS systems augmented with structural data. Thus, this study aims to examine the evolution of LuxS protein on a phylogenetic, as well as structural perspective.

## Results and discussion

### Genome-wide survey of LuxS protein sequences

LuxS homologues were searched using Hidden Markov Models [13]. The number of hits retrieved from the sequence search is 3106 and the phyletic distribution is shown in Additional file 1. High abundance of LuxS protein sequences in phyla such as *Actinobacteria*, *Firmicutes*, *Gamma-Proteobacteria* and *Bacteroidetes* could be observed. However, no LuxS homologues could be identified in many phyla, such as *Chloroflexi*, *Aquificae*, *Thermotogales*, *Cyanobacteria* etc. (Additional file 2). LuxS homologues could not be identified in certain pathogenic bacteria, such as *Mycobacterium tuberculosis, Chlamydia trachomatis*, *Mycoplasma pneumoniae,* and in Archaea, using the current search protocol. However, genome-wide survey of Pfs-protein, another protein involved in AMC that catalysed conversion of S-Adenosyl Homocysteine to S-Ribosyl Homocysteine, conducted with similar parameters using phmmer search program [13], yielded about 8000 positive hits (data not shown). Discrepancy in the number of hits, despite both of the proteins being a part of the same metabolic pathway, suggests the possibility of other enzymes playing a similar role as LuxS in organisms where no homologues could be observed.

### Phylogeny of LuxS protein

**Fig. 1:**
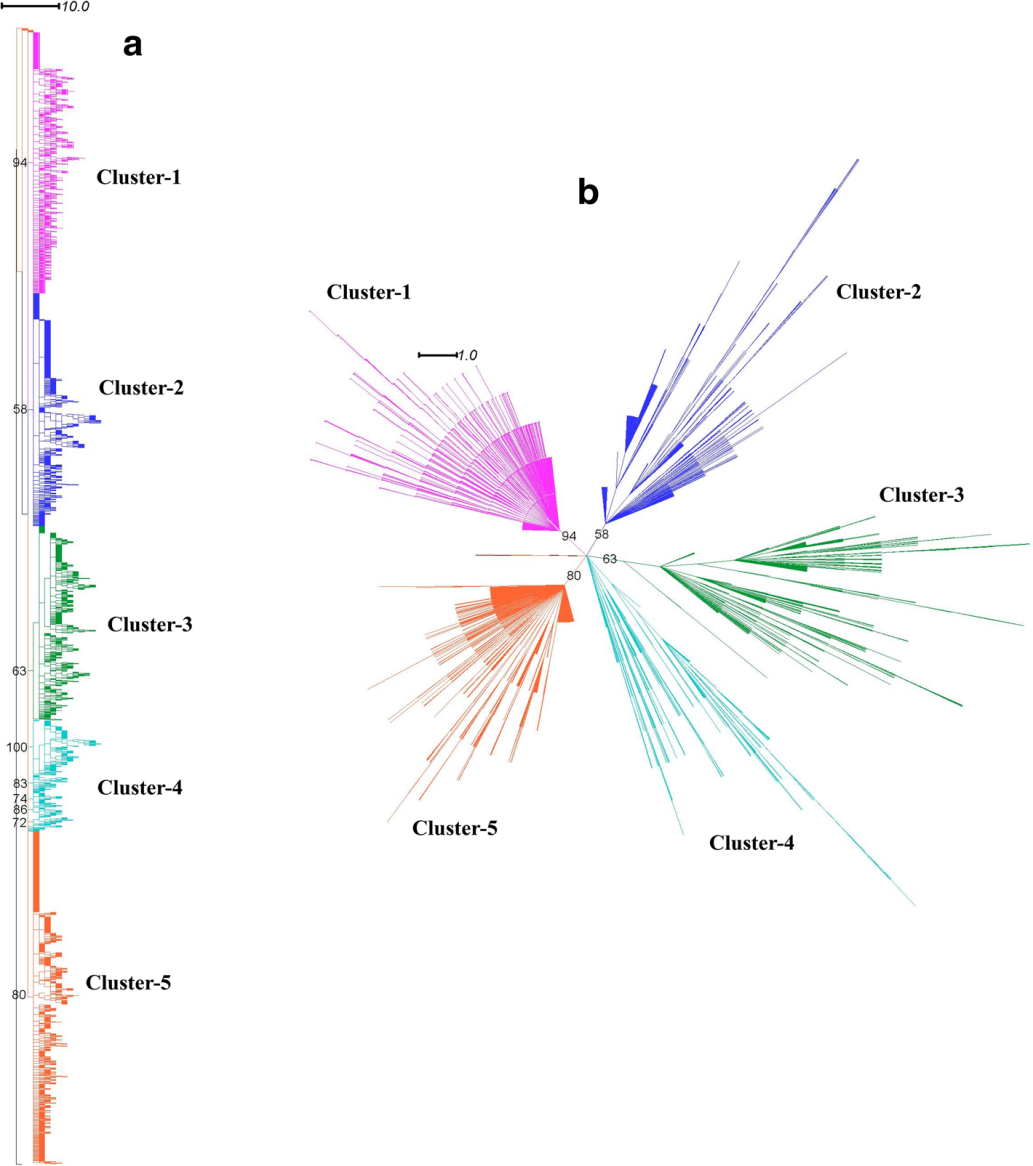
**a** Cladogram of LuxS protein sequences of different bacterial species constructed by Maximum Likelihood method with 100 bootstrap replicates; **b** Dendrogram of LuxS protein sequences constructed with above mentioned methods

We performed phylogenetic analysis of 3106 LuxS protein sequences identified from different bacterial genomes. The phylogenetic tree showed grouping of sequences into five distinct clusters, which has not been reported in previous studies to the best of our knowledge (Fig. 1a and b). The absence of phylum-specific clusters and co-clustering of LuxS sequences of different bacterial phyla confirms previous reports of high degree of conservation of LuxS sequences among different bacterial species [10]. Taxonomic distribution of LuxS protein shows grouping of *Proteobacterial* sequences in the fifth cluster. The *Firmicute* sequences are spread throughout the remaining five clusters, while *Actinobacterial* sequences are distributed in second and third clusters. The sequences of *Bacteroidetes*, *Spirochaetes*, *Fusobacteria* are present in Cluster-1, while the sequences of phylum *Dienococcus-Thermales* is present in the Cluster-4 (Additional files 3 and 4). There are a few cases where LuxS protein may have evolved by horizontal gene transfer events, such as in *Helicobacter pylori*, which belongs to *Epsilon-Proteobacteria*. LuxS sequence of *H. pylori* is grouped in the second cluster. This observation of distinct clustering of *H. pylori* LuxS sequences (in Cluster-2) could be reasoned that *H. pylori* may have acquired LuxS gene from another species, within Cluster-2, by means of horizontal or lateral gene transfer. Another such case is grouping of many LuxS sequences from *Actinobacteria* in Cluster-3. These sequences are of various species from *Bifidobacteria, Acidaminococcus* and *Gardenerella* genera. This observation is in line with previous study on LuxS proteins in *Bifidobacterium* genus, even though the study suggests the close homology of *Bifidobacterium* LuxS sequences with LuxS sequence of *Vibrio harveyi* [14].

We also analysed the LuxS sequence clusters according to their habitat distribution (Additional files 3 and 5) and found that most of the sequences in Cluster-1 were gut endosymbionts in mammals and other animals, while many sequences in Cluster-2 were halophiles, alkaliphiles and psychrophiles. Many LuxS sequences in Cluster-3 are from plant/food associated bacteria and many LuxS sequences were of extremophilic origin in Cluster-4 (including thermophiles, alkaliphiles, deep-sea hydrothermal vent residents and psychrophiles). In Cluster-5, many LuxS sequences were gut-associated bacteria, marine inhabitants or were plant pathogens. In general, LuxS sequences of pathogenic bacteria were distributed across Clusters-1, 2, 3 and 5, but were not observed in Cluster-4. The grouping of many *Actinobacterial* and *Firmicute* sequences in different clusters hint at the evolution of the protein through a series of lateral gene transfers. Nevertheless, the classification of LuxS sequences according to their habitat distribution suggests that LuxS protein may also have evolved through convergent evolution, particularly in case of LuxS sequences of Firmicute species.

### Gene synteny analyses of LuxS genes

It is well-known that genes that are involved in the pathway of quorum sensing occur as chromosomal neighbours. Hence, we examined for genes neighbouring to LuxS genes in different bacterial genomes. We observed that majority of genes neighbouring to LuxS genes (present upstream, as well as downstream of LuxS gene) are those annotated as hypothetical proteins (Additional files 6 and 7). We performed Multiple Sequence Alignment (MSA) of the hypothetical proteins to examine whether these genes, coding for hypothetical proteins, have a significant common evolutionary origin, and found that they do not have any identical or similar sequences, and thus remain different from each other. However, the hypothetical protein coding genes were functional neighbours, i.e., they were a component of an operon along with the LuxS gene in only 6 of 36 genomes. We also observed that genomes of phyla *Gamma-Proteobacteria*, and *Bacteroidetes* tend to have similar syntenies, though this pattern is not observed in all the phyla (Additional file 6). All members of *Gamma-Proteobacteria* have gshA gene (coding for Glutamine-cysteine ligase) upstream of LuxS, and both members of the phylum *Bacteroidetes* have rpsO (coding for Ribosomal subunit protein S15) downstream of LuxS gene. One possible reason for this may be that there is an evolutionary constraint for species of *Gamma-Proteobacteria* and *Bacteoidetes* to retain the order of genes conserved. The biological aspect behind this constraint has to be further investigated.

Moreover, in many species considered in our analysis, genes that are important for survival of the organism are present downstream of LuxS gene, such as gene coding for DNA-protecting protein in *Geobacillus thermodenitrificans* (Radiation-resistant bacteria of Bacillales order), another on cell wall-associated hydrolase in *Clostridium acetobutylicum* and third for hemolysin in *Vibrio cholerae* (Additional file 6). These genes may be unique to bacterial-species in question, or hold high functional significance for the organism in question.

### Motif analyses of LuxS sequences from different clusters

**Fig. 2:**
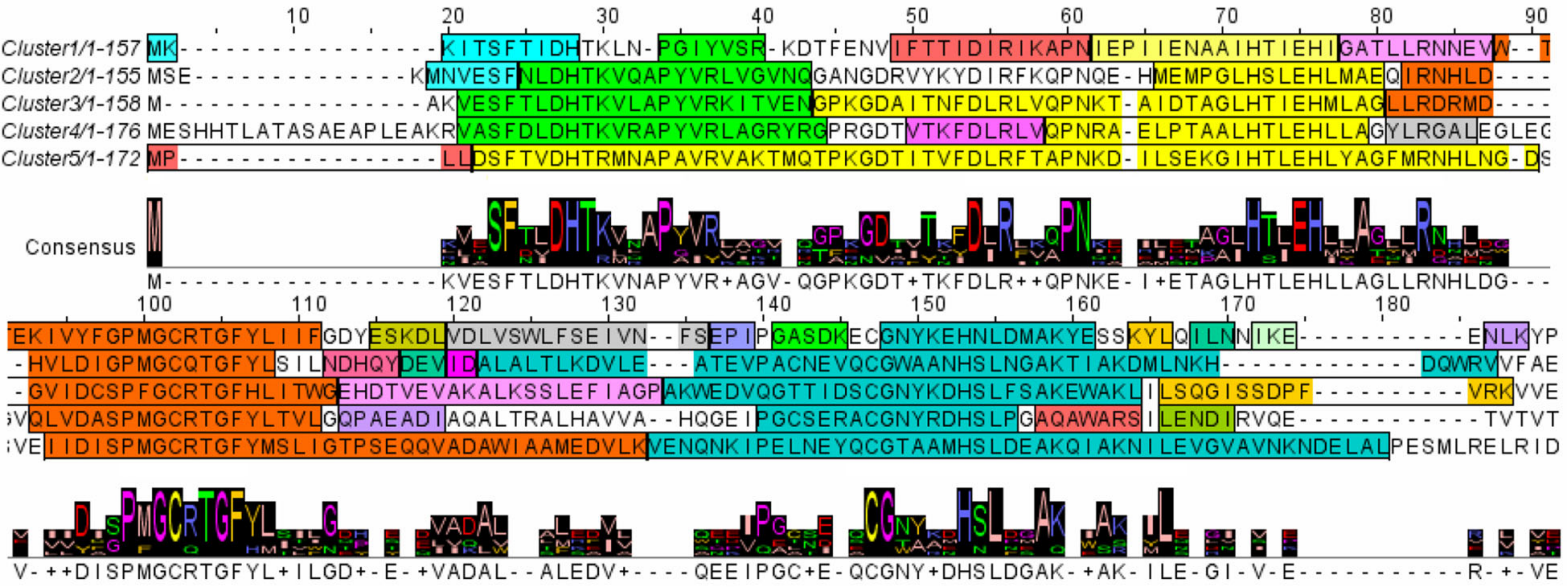
LuxS protein sequences of bacterial species from each cluster, showing conserved structural motifs. Each colour corresponds to a conserved motif. Motifs having same colour in all the five sequences represents motifs common to sequences from all the five clusters, while motifs having non-equivalent colours represent motifs unique to the sequences of the cluster

We next examined unique conserved motifs in LuxS sequences of each cluster obtained from the phylogenetic tree. We found the presence of motifs conserved in all the five clusters, and motifs unique to each cluster were also seen (Fig. 2). For example, structural motif corresponding to the first α-helix of LuxS protein is conserved in LuxS proteins of the entire five clusters. However, Cluster-1 has 15 conserved motifs, Cluster-2 has 8 conserved motifs, Cluster-3 each has 6 conserved motifs, Cluster-4 has 9 motifs and Cluster-5 has 6 conserved motifs.

### Surface electrostatic potential analysis of LuxS protein of *Bacillus subtilis* 168 (PDB Id: 1J98)

**Fig. 3:**
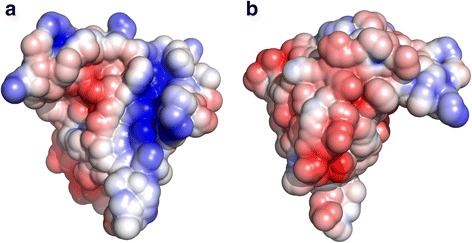
Structure of LuxS protein of *B. subtilis* (PDB Id: 1J98) showing surface electrostatic potentials from front view (**a**) and back view (**b**)

The electrostatic surface potential was next analyzed using the crystal structure of LuxS protein of *Bacillus subtilis* (PDB id: 1J98). We observed the presence of a patch of positively charged potential, comprising residues on the first and second β-strand (K25, K35 and R39) (Fig. 3, Additional file 8). It has also been observed in the crystal structure of LuxS protein of *Deinococcus radiodurans* that these residues are involved in conformational changes that facilitate binding of the substrate to the active site [15]. Therefore, these interactions may contribute to the structural stability of the LuxS homodimer. Negative electrostatic potential can also be seen around the metal-ion binding motif (HXXEH), and on the region behind the metal-ion binding motif, that corresponds to the surface-exposed residues of second helix, and a mixture of negative, as well as positive electrostatic potentials can be observed on surface-exposed residues of third helix. Presence of negative electrostatic potentials on the residues near the N-terminus further confirms the fact that these residues may facilitate binding of the substrate in the dimer form of LuxS protein, as discussed by Ruzheinikov and coworkers [8]. It has been reported that a small RNA molecule MicA is involved in biofilm formation in *Salmonella enterica* and is located in close proximity to LuxS gene [16]. It has also been reported that certain peptides can bind to LuxS protein and inhibit its activity partially in *Streptococcus suis* [17]. Our results might suggest the possible mode of interaction of LuxS proteins with these molecules.

### Homology modelling of representative LuxS protein sequences from each cluster and surface electrostatic potential analyses

We next generated homology models of LuxS protein sequences of *Borrelia burgdorferi, Amphibacillus jilinensis, Lactobacillus plantarum, Truepera radiovictrix* and *Vibrio harveyi*, belonging to Clusters 1–5, respectively, using MODELLER program [18–20] (Additional files 9 and 10). Twenty models were generated for each sequence, starting from LuxS crystal structure of *B. subtilis* as a template retrieved from PDB database [21]. The best model was selected based on MODELLER/DOPE scores. Multiple structural alignment of the homology models has shown that motifs having secondary structural elements have similar spatial orientation. In contrary, N- terminus and C-terminus regions (shown in blue and red respectively in Additional file 10), the region between first α-helix and third β-strand, as well as the loop regions between second and third α-helices (shown in green and orange, respectively, in Additional file 10) were found to have structural deviations. Around 67 % of residues of reference structure were found to be equivalent, when the superposed homology models were compared at 0.8Å threshold.

A multiple structural alignment of homology models with LuxS crystal structure of *B. subtilis* as template, followed by clustering on the basis of Lesk-Hubbard (L-H) structural parameters, shows the formation of two main clusters of homology models (Additional file 11). Homology models of LuxS proteins from *Vibrio harveyi*, *Amphibacillus jilinensis* and LuxS crystal structure of *Bacillus subtilis* tend to form one cluster, which implies that these three proteins are likely to be similar to each other. This is also supported by their higher identities in their sequences (average sequence identity of 43 %). Similarly, homology models of LuxS proteins of *Lactobacillus plantarum* and *Truepera radiovictrix* formed a distinct cluster, leaving behind model of LuxS protein of *Borrelia burgdorferi* unclustered with other protein models. Formation of clusters in LH plot provides some perspective on the structural distances of LuxS protein. The N-terminal region of *T. radiovictrix* was found to be structurally dissimilar with respect to same regions of other homology models. Indeed, the model of *T. radiovictrix* tends to be present on one side of the L-H plot.

We next examined the surface electrostatic potentials of the homology models in order to examine any difference in their profiles amongst the models. We observed the presence of negative surface electrostatic potentials at regions corresponding to metal binding motif (HXXEH) at the first helix, and we also observed negative electrostatic potentials on residues of second and third helices in all five homology models, *albeit* at low conservation. This further supports our inference that the second helix may be an interacting partner with regulatory proteins or small-RNAs, as negative electrostatic potentials seem to be more conserved than positive electrostatic potentials (Additional files 12 and 13). Presence of negative and positive electrostatic potentials on the residues near N-terminus signifies that these residues may be important for the structural stability of the protein, even though this region is structurally dissimilar as seen in the multiple structural alignment. Similarly, presence of negative electrostatic potential on residues next to the metal-ion binding residue (C133 on LuxS protein of *B. subtilis*) show that these residues are involved in conformational change and interaction with the substrate. Though it was seen in the surface potential map of LuxS protein of *B. subtilis* that some residues in the first and second β-strand contribute to the structural stability of LuxS homodimer, similar potentials were not observed in the homology models, even though corresponding residues are conserved, as in the block corresponding to R39 of LuxS protein of *B. subtilis*. These may be electrostatic potentials unique to proteins of each cluster.

## Conclusion

In this study, we investigated the evolution of LuxS protein through a phylogenetic and structural perspective. Molecular phylogeny studies on 3106 LuxS protein sequences, an important enzyme in Autoinducer-2 biosynthesis of different bacteria species, has shown grouping of sequences into five distinct clusters. These LuxS protein sequences appear to be grouped on the basis of their habitats and lifestyles, which may be a case of convergent evolution. Synteny analysis of LuxS genes has shown the presence of large number of neighbouring genes annotated as hypothetical proteins suggesting a broader repertoire of biological functions are yet to be discovered. Furthermore, many genes that may be critical for survival of the organism are present downstream of LuxS gene. On the structural front, surface electrostatic analysis of LuxS protein of *B. subtilis* shows the presence of regions having positive and negative electrostatic potentials, which contribute to the structural stability of LuxS homodimer and may be the sites of protein-protein and protein-RNA interactions. Homology modelling of LuxS protein sequences from each cluster shows the similarities as well as differences among LuxS proteins of different clusters, which are more clear when the models are subjected to structural alignment and analyses of surface electrostatic potentials. Structural alignment showed the grouping of models into two clusters, which may provide some clues about evolution of LuxS protein from a structural perspective. Molecular phylogeny analysis of LuxS protein on evolutionary as well as structural perspective has yielded some insights into the evolution of enzymes involved in biosynthesis of Autoinducers. However, further studies are required to obtain a clearer picture of evolution of quorum-sensing apparatus in bacteria.

## Methods

### Sequence search

The LuxS protein sequence of *Bacillus subtilis* 168 (UniProt Id: O34667) was used as a query to retrieve LuxS sequences of different bacterial phyla (*Bacteroidetes*, *Chloroflexi*, *Dienococcus*-*Thermus*, *Fusobacteriales*, *Alpha*-*Proteobacteria* and *Haloplasmatales*) using BLASTp program of NCBI [22]. The resulting 20 sequences from the BLASTp sequence searches were used as queries again to search for other LuxS homologues against Non-Redundant database using phmmer program (version 1.4) [13]. The results were retrieved and merged to eliminate redundancy.

### Phylogeny of LuxS protein

The non-redundant LuxS homologues were then aligned by PROMALS3D multiple alignment web server [23]. The consensus regions were identified, and non-consensus regions were removed using Jalview (version: 2.8.2) [24]. The phylogenetic tree was constructed by Maximum Likelihood method using RAxML program [25] with 100 bootstrap replicates (version 8.0.0). The resulting tree was visualised and edited using Dendroscope program (version 3.2.10) [26].

### Gene synteny analysis of LuxS gene

The gene locations of different bacterial species were examined in BioCyc database collection (version 19.0) [27] in order to examine the location of LuxS genes in different bacterial genomes. Information regarding gene position, location in operon, particulars of genes located upstream and downstream of LuxS gene was noted, SCOP classification was obtained by SUPERFAMILY HMM library and genome assignment server (version 1.75) [28, 29]. The hypothetical proteins were aligned using ClustalW algorithm [30] of MEGA6 program [31] to analyse the similarities between the hypothetical proteins present downstream of LuxS gene,

### Motif analysis of LuxS protein

The unique gi-identification numbers of sequences in each cluster were used as search terms to retrieve the corresponding sequences in .fasta format from the NCBI-protein database. The retrieved sequences were analysed for conserved structural motifs using MOTIFS program [32]. The motifs were mapped to MSA of the protein sequences from each cluster generated by T-coffee program [33] using Jalview program (version 2.8.2) [24].

### Analysis of surface electrostatic potentials of *Bacillus subtilis* LuxS protein (PDB Id: 1J98)

Crystal structure of LuxS protein of *B. subtilis* was submitted to the PDB2PQR web server [34, 35] and surface electrostatic potential calculations were performed using Adaptive Poisson Boltzmann Solver (APBS) plugin [36] of Pymol program [37]. The results were visualised using the Pymol program [37], and corresponding residues having positive and negative electrostatic potentials were mapped using Pymol program [37].

### Homology modelling of LuxS proteins of different species and analyses of surface electrostatic potential analyses

Query LuxS protein sequences (marked forest green in the LuxS phylogenetic tree showing taxonomic distribution) were selected from each cluster of the phylogenetic tree. The sequences were retrieved in PIR format. The sequences were searched for closest homologues in PDB database [21] using NCBI-BLASTp search program [22], and resulting highest scoring hit was used as template for modelling. 20 homology models were obtained from MODELLER program (version 9.14) [18–20]. The models were evaluated and the best model was chosen based on MODELLER scores. Ramachandran plot of the candidate model was mapped by Rampage program [38] (Additional file 10). Furthermore, the resulting homology models were aligned using MUSTANG multiple structural alignment server [39] with crystal structure of LuxS protein of *B. subtilis* (PDB Id: 1J98) used as reference. The superposed structure was visualised using Pymol program [37], and the Lesk-Hubbard plot of C^α^ atoms vs. RMSD and sieved structure of reference protein (Crystal structure of LuxS protein of *B. subtilis*) set to 0.8Å threshold was retrieved (Additional file 12).

PQR results were obtained for the best homology model of cluster representatives, exactly as done for the crystal structure. The multiple structure-based sequence alignment of homology models, generated by MUSTANG structural alignment server [39], was used to map these potentials to their respective residues using Jalview program (version 2.8.2) [24].

## Additional files

Additional file 1: Table containing Number of hits obtained from each sequence searches and phylum-wise distribution of LuxS sequences obtained from phmmer searches. (XLSX 39 kb) Additional file 2: Table showing Taxonomic distribution of LuxS sequences in bacteria. (XLSX 38 kb) Additional file 3: Table showing Gene-synteny information of LuxS genes of different species. (XLSX 41 kb) Additional file 4: Phylogenetic tree of LuxS sequences showing taxonomic distribution of sequences. Colour codes: Blue: *Firmicutes*; Pink: *Actinobacteria*; Yellow: *Bacteroidetes*; Cyan: *Spirochaetes*; Red: *Proteobacteria*; Brown: *Fusobacteria*; Grass Green: *Deinococcus*-*Thermales*; Forest green: Query sequences selected for homology modelling. (PDF 2242 kb) Additional file 5: Phylogenetic tree of LuxS sequences showing habitat distribution of sequences. Colour codes: Pink: Gut/Faecal microorganisms; Purple: Animal and Human pathogens; Brown: Soil-dwelling bacteria; Yellow-green: Food-associated bacteria; Forest-green: Plant-associated bacteria; Sky blue: Halophiles; Navy blue: Alkaliphiles; Cyan: Psychrophiles; Orange: Thermophiles; Grey: Radiation-resistant bacteria; Yellow: Hydrothermal vent inhabiting bacteria; Lignt blue: Marine bacteria; Blue-green: Plant pathogens; Black: Habitat information not available (PDF 108 kb) Additional file 6: Cladograms of LuxS protein sequences showing (A) taxonomic distribution of species (Colour code: Blue: *Firmicutes*; Pink: *Actinobacteria*; Yellow: *Bacteroidetes*; Cyan: *Spirochaetes*; Red: *Proteobacteria*; Brown: *Fusobacteria*; Green: *Deinococcus*-*Thermales*) and habitat distribution of bacteria possessing the LuxS proteins (B), colour codes: Pink: Gut/Faecal microorganisms; Purple: Animal and Human pathogens; Brown: Soil-dwelling bacteria; Yellow-green: Food-associated bacteria; Forest-green: Plant-associated bacteria; Sky blue: Halophiles; Navy blue: Alkaliphiles; Cyan: Psychrophiles; Orange: Thermophiles; Grey: Radiation-resistant bacteria; Yellow: Hydrothermal vent inhabiting bacteria; Light blue: Marine bacteria; Blue-green: Plant pathogens; Black: Habitat information not available). (PDF 2242 kb) Additional file 7: Pie chart showing distribution of SCOP protein superfamily-coding genes present as a component of an operon (A), present upstream (B) and present downstream (C) with respect to the LuxS gene in different bacterial species. (PDF 553 kb) Additional file 8: Pymol session file showing APBS map of LuxS protein of *Bacillus subtilis* (PDB Id: 1J98). As per the usual convention, blue regions correspond to the residues having positive surface electrostatic potentials, while red regions correspond to the residues having negative surface electrostatic potentials. (PSE 29862 kb) Additional file 9: Folder containing homology models of LuxS proteins of *Borrelia burgdorferi* (Cluster-1), *Amphibacillus jilinensis* (Cluster-2), *Lactobacillus plantarum* (Cluster-3), *Truepera radiovictrix* (Cluster-4) and *Vibrio harveyi* (Cluster-5); Multiple structural alignment of homology models; Sieved structure of LuxS protein of *B. subtilis* against 0.8Å threshold from the multiple structural alignment. (ZIP 677 kb) Additional file 10: Homology models of LuxS of representatives from the clusters and Ramachandran plots of homology models. (ZIP 936 kb) Additional file 11: Lesk-Hubbard plot of the multiple structural alignment of the homology models. (PDF 40 kb) Additional file 12: Multiple structure-based sequence alignment of homology models of LuxS protein sequences, showing surface electrostatic potentials. Residues labelled in red are regions with negative surface potentials; residues labelled in blue are regions having positive surface electrostatic potentials. (PDF 157 kb) Additional file 13: Folder containing pymol session files showing APBS maps of the homology models. Colour coding is same as in Additional file 8. (ZIP 35165 kb)

## Abbreviations

AI: Autoinducer
DOPE: Discrete Optimized Protein Energy
HMM: Hidden Markov Model
MSA: Multiple Sequence Alignment
NCBI: National Center for Biotechnology Information
PROMALS3D: PROfile Multiple Alignment with predicted Local Structures and 3D constraints
RAxML: Randomized Axelerated Maximum Likelihood
