# Supplementary figures and images for: Genome-wide survey and phylogeny of S-Ribosylhomocysteinase (LuxS) enzyme in bacterial genomes
Source: BMC Genomics. 2016 Sep 20;17:742. doi: 10.1186/s12864-016-3002-x (PMC5029033; doi:10.1186/s12864-016-3002-x)

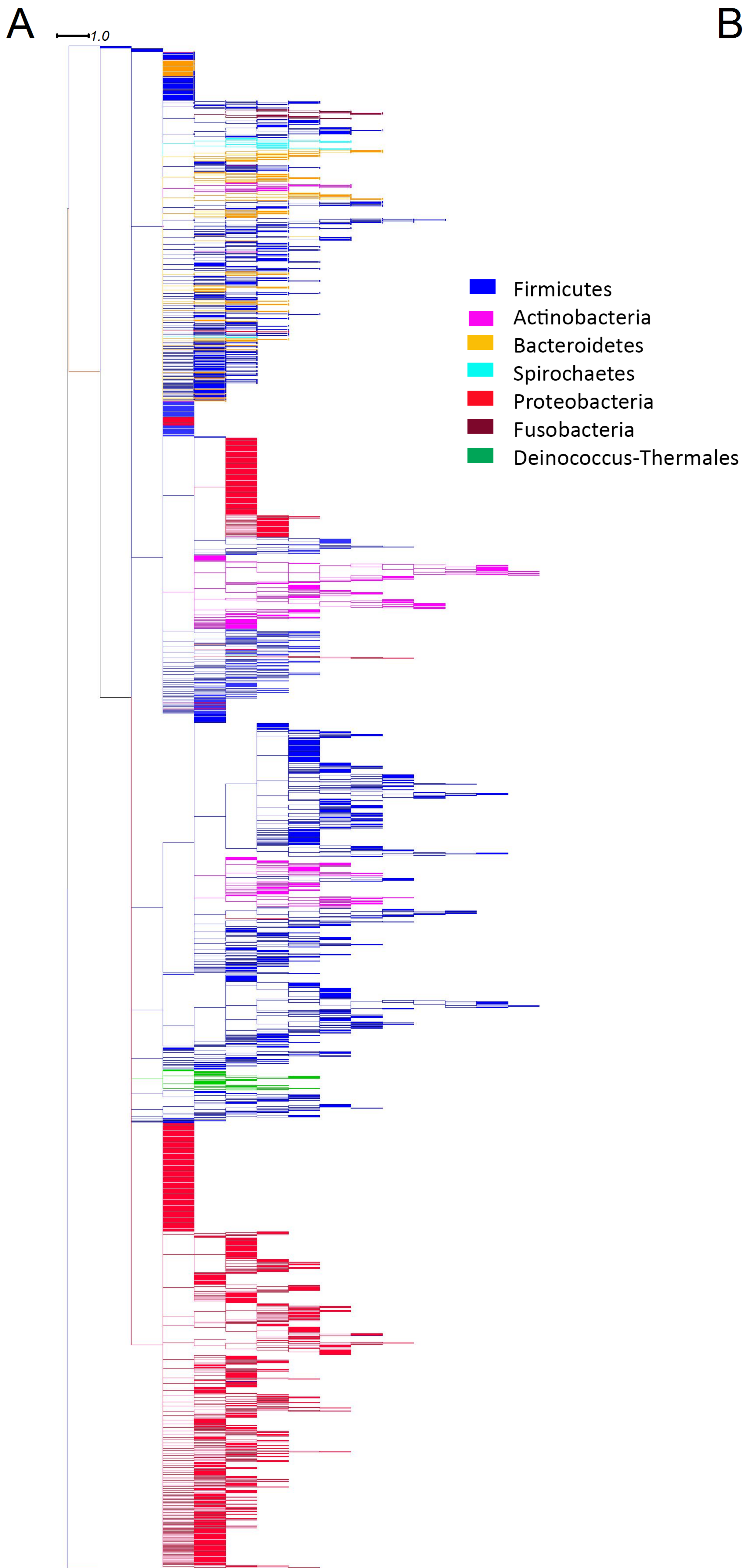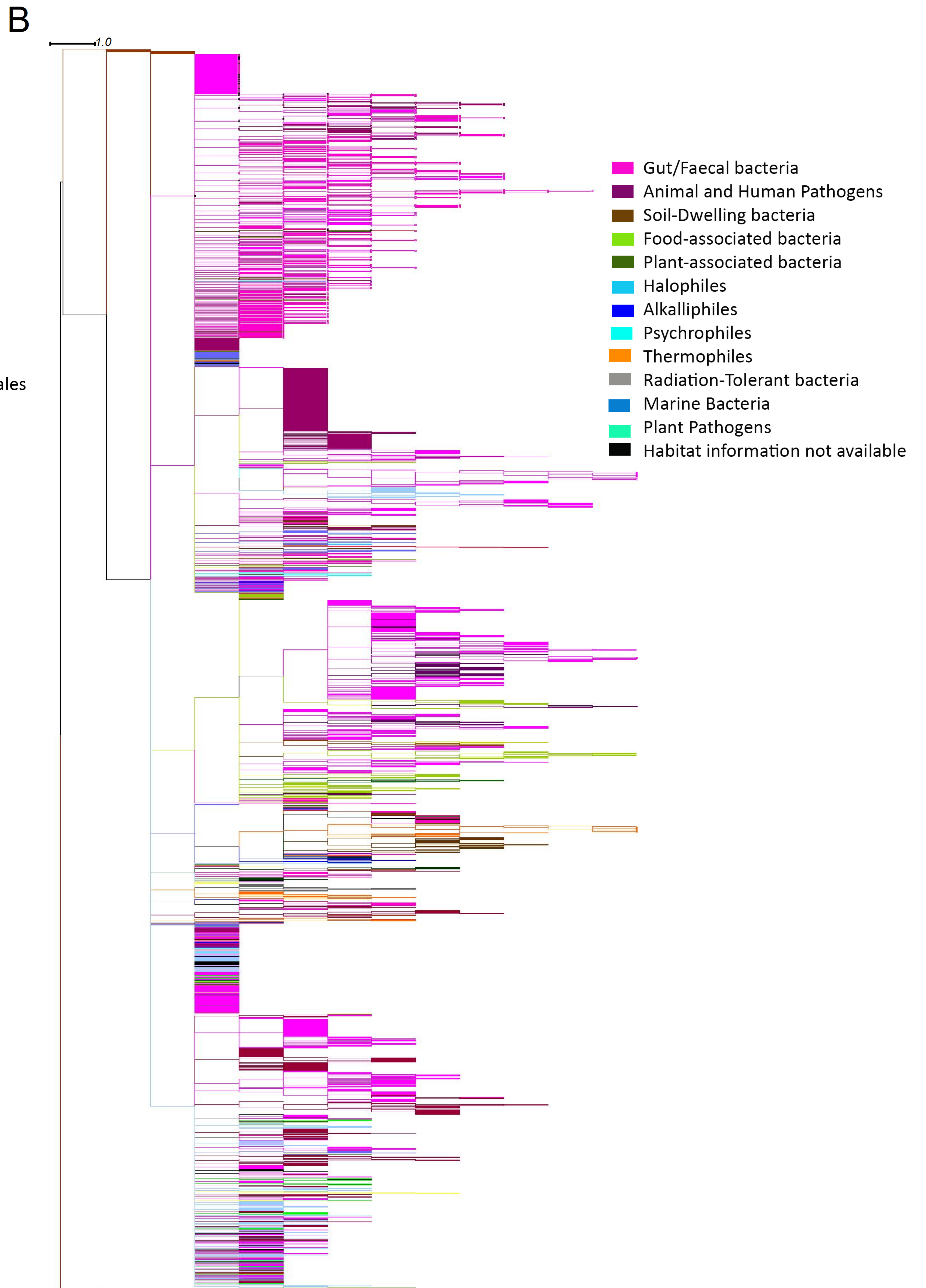

Supplement: Additional file 4: — Phylogenetic tree of LuxS sequences showing taxonomic distribution of sequences. Colour codes: Blue: Firmicutes; Pink: Actinobacteria; Yellow: Bacteroidetes; Cyan: Spirochaetes; Red: Proteobacteria; Brown: Fusobacteria; Grass Green: Deinococcus-Thermales; Forest green: Query sequences selected for homology modelling. (PDF 2242 kb) [file 12864_2016_3002_MOESM4_ESM.pdf]

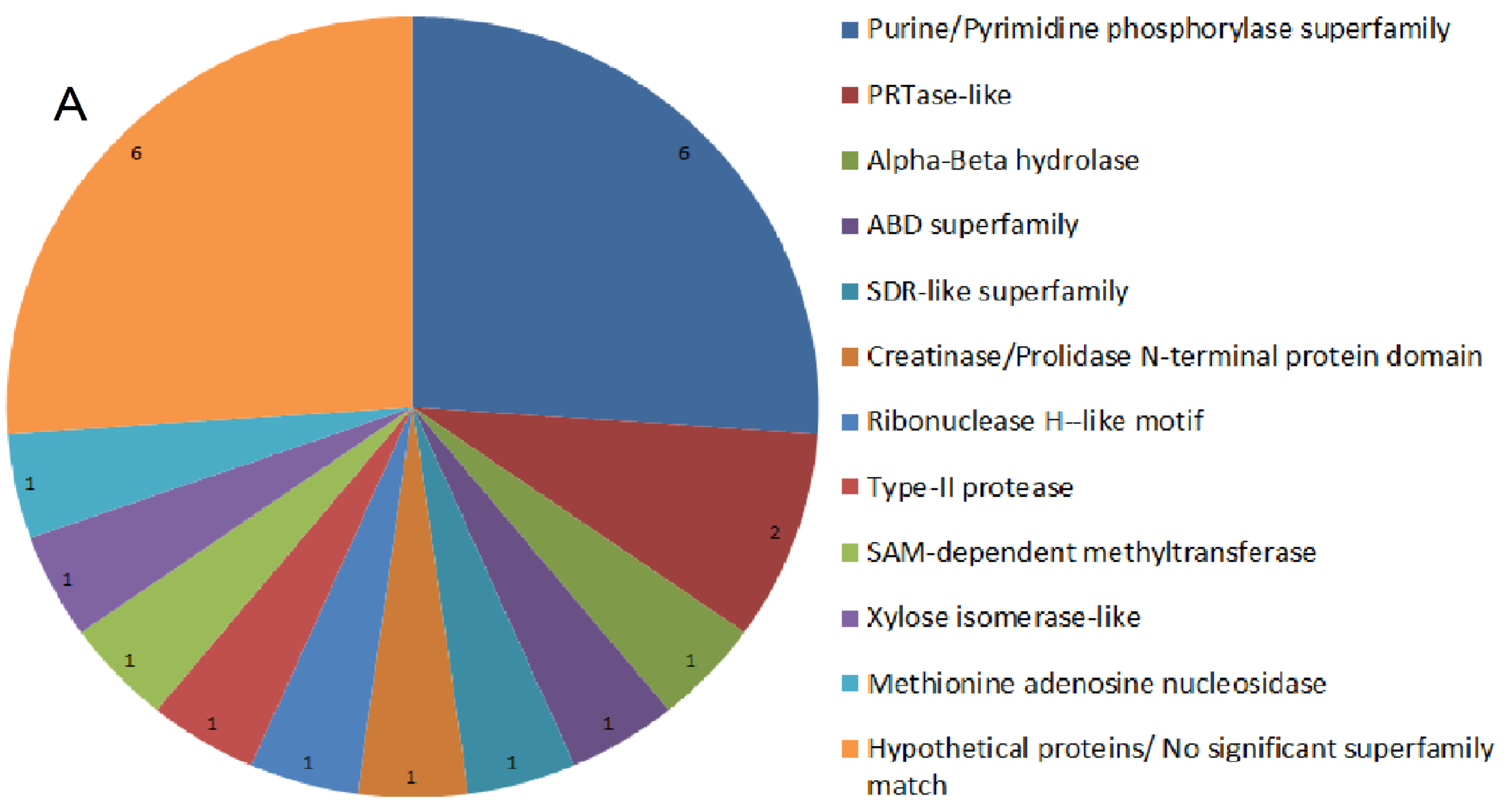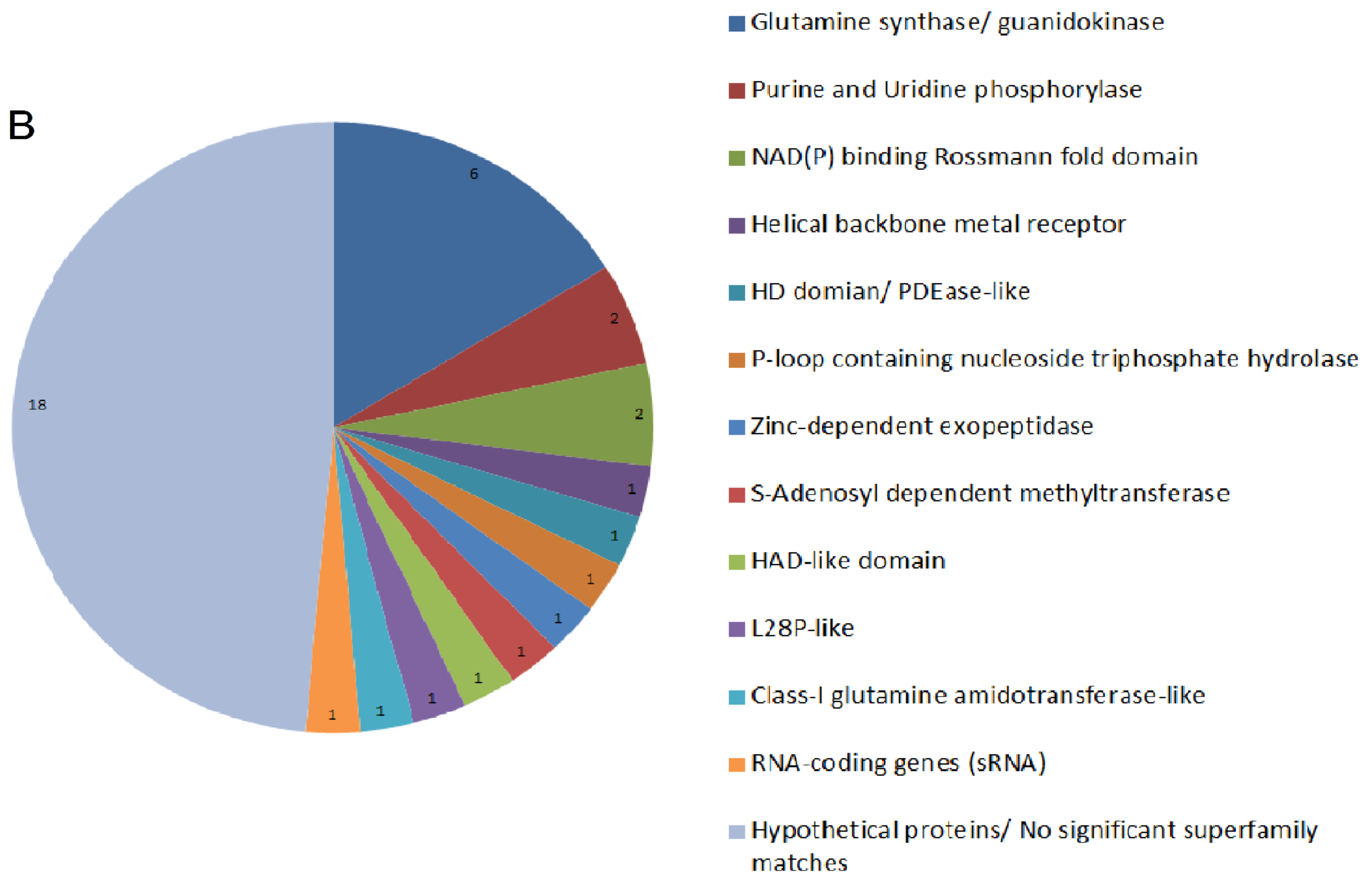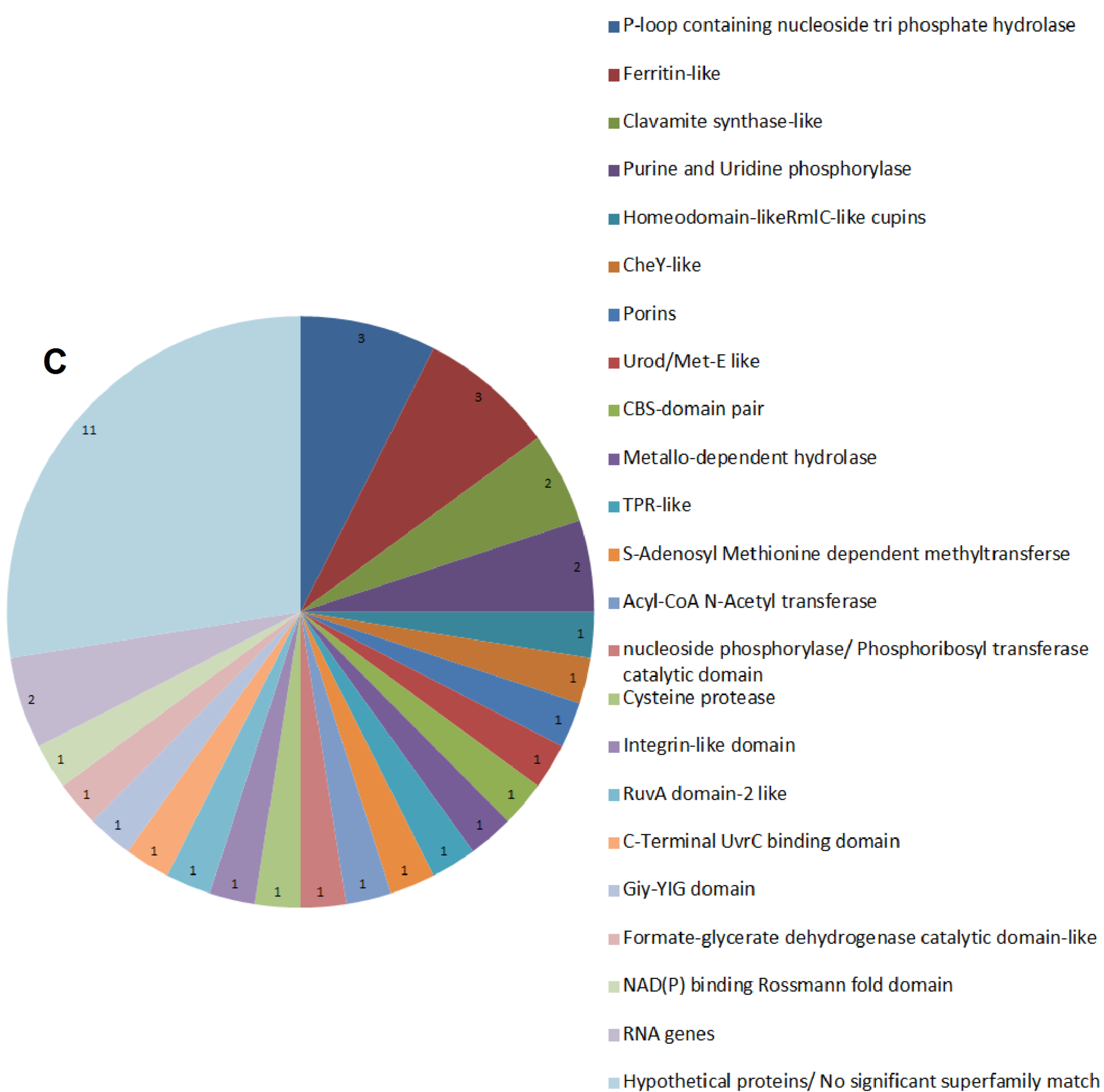

Supplement: Additional file 7: — Pie chart showing distribution of SCOP protein superfamily-coding genes present as a component of an operon (A), present upstream (B) and present downstream (C) with respect to the LuxS gene in different bacterial species. (PDF 553 kb) [file 12864_2016_3002_MOESM7_ESM.pdf]
