# Supplementary material for: Genome-wide survey and phylogeny of S-Ribosylhomocysteinase (LuxS) enzyme in bacterial genomes
Source: BMC Genomics. 2016 Sep 20;17:742. doi: 10.1186/s12864-016-3002-x (PMC5029033; doi:10.1186/s12864-016-3002-x)
Supplement: Additional file 10: — Homology models of LuxS of representatives from the clusters and Ramachandran plots of homology models. (ZIP 936 kb) [file 12864_2016_3002_MOESM10_ESM.zip › Additional_file_10/Amphibacillus_jilinensis.pdf]

RAMPAGE: Assessment of the Ramachandran Plot

File: Amphibacillus_jilinensis.pdb

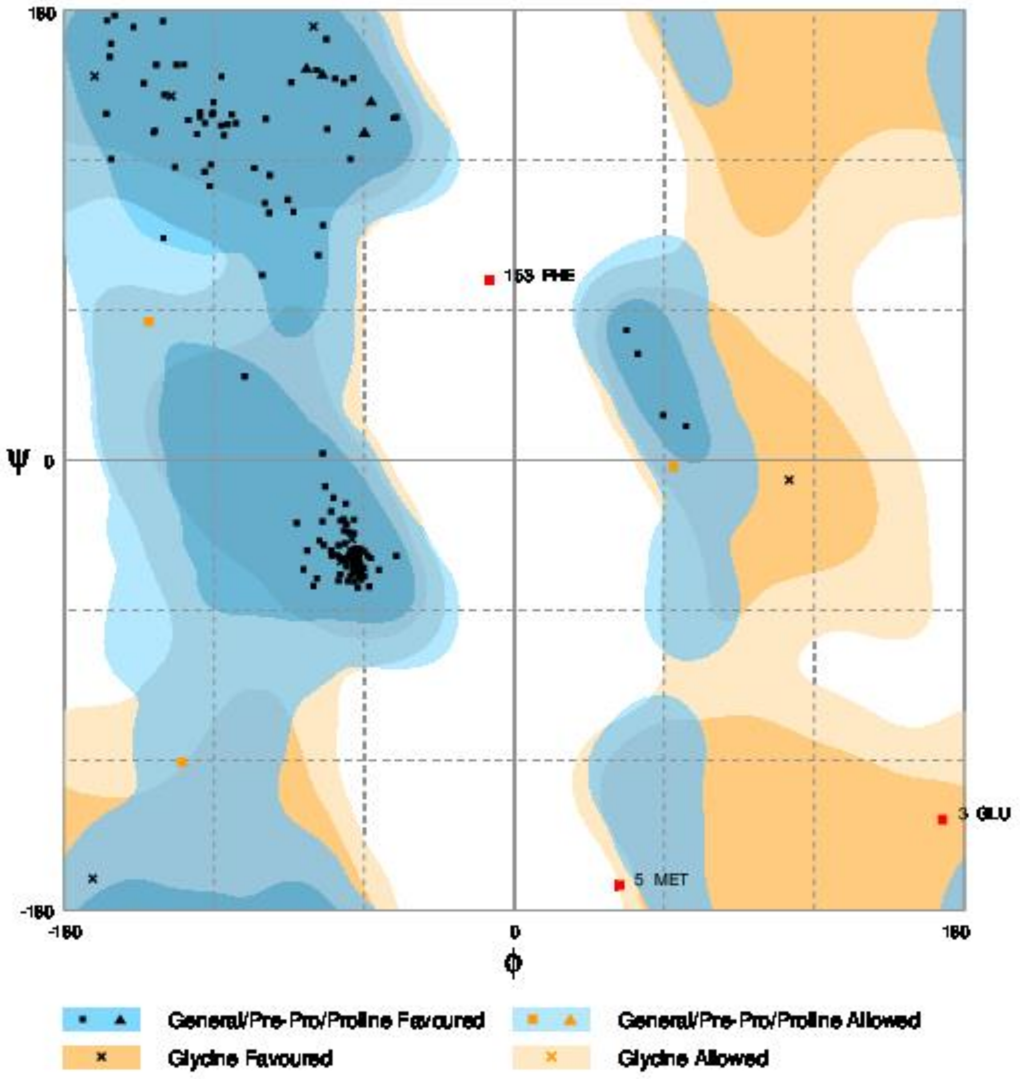

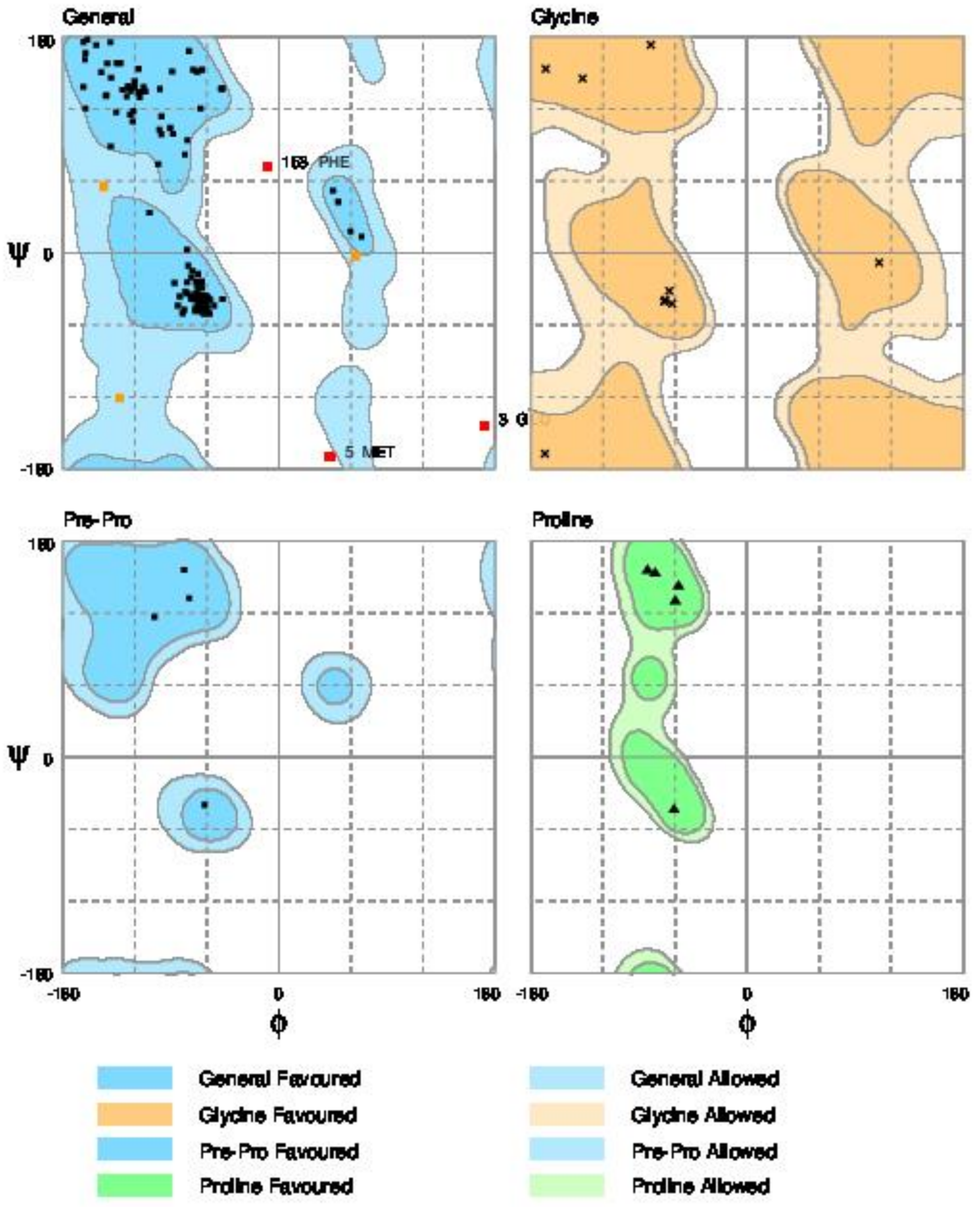

Evaluation of residues

```
Residue [   47 :ASN] (  63.80,   -2.71) in Allowed region
Residue [   94 :HIS] (-146.07,   55.40) in Allowed region
Residue [  154 :ALA] (-132.73,-120.77) in Allowed region
Residue [    3 :GLU] ( 171.37,-143.70) in Outlier region
Residue [    5 :MET] (  42.39,-169.90) in Outlier region
Residue [  153 :PHE] (  -9.63,   71.98) in Outlier region
Number of residues in favoured region    (~98.0% expected)  :  147 ( 96.1%)
Number of residues in allowed region     ( ~2.0% expected)  :    3 (  2.0%)
Number of residues in outlier region                        :    3 (  2.0%)
```
