# Supplementary material for: Genome-wide survey and phylogeny of S-Ribosylhomocysteinase (LuxS) enzyme in bacterial genomes
Source: BMC Genomics. 2016 Sep 20;17:742. doi: 10.1186/s12864-016-3002-x (PMC5029033; doi:10.1186/s12864-016-3002-x)
Supplement: Additional file 10: — Homology models of LuxS of representatives from the clusters and Ramachandran plots of homology models. (ZIP 936 kb) [file 12864_2016_3002_MOESM10_ESM.zip › Additional_file_10/Borrelia_burgdorferi.pdf]

RAMPAGE: Assessment of the Ramachandran Plot

File: Borrelia_burgdorferi.pdb

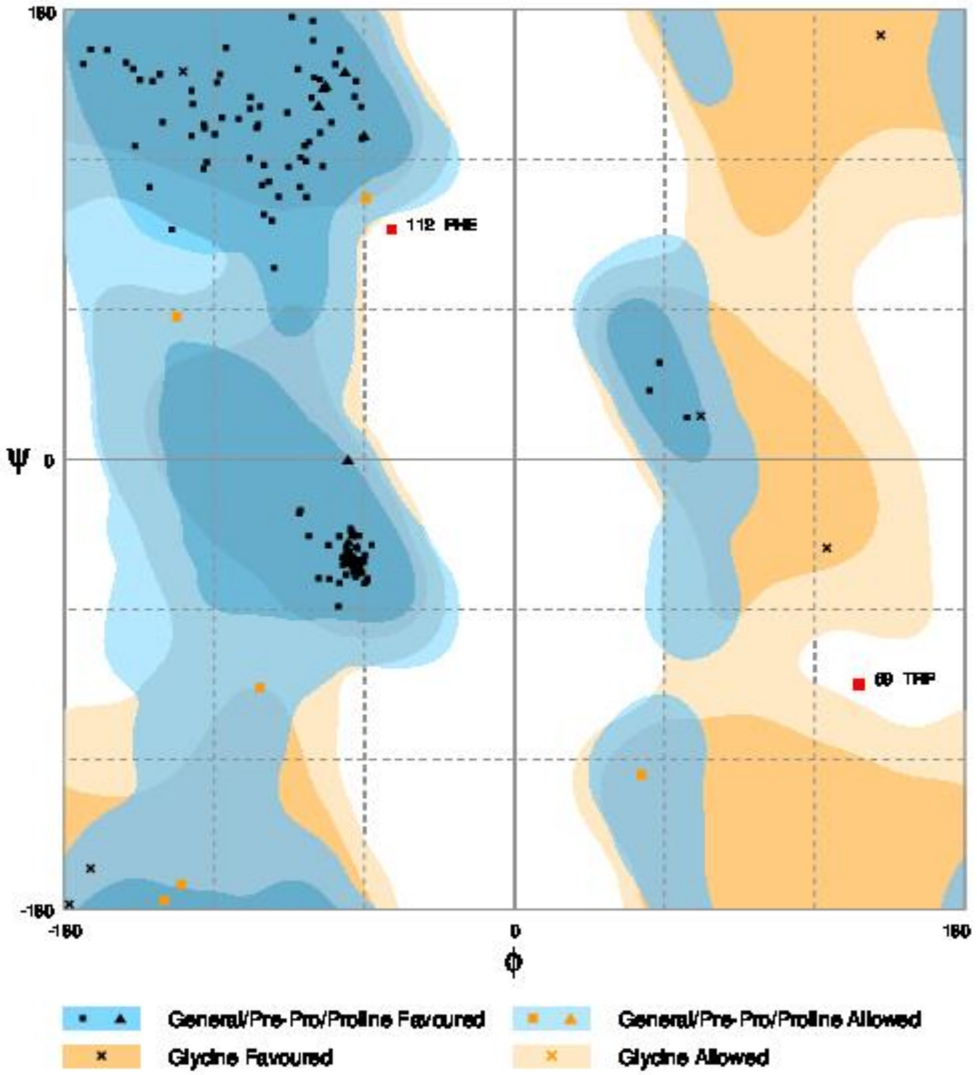

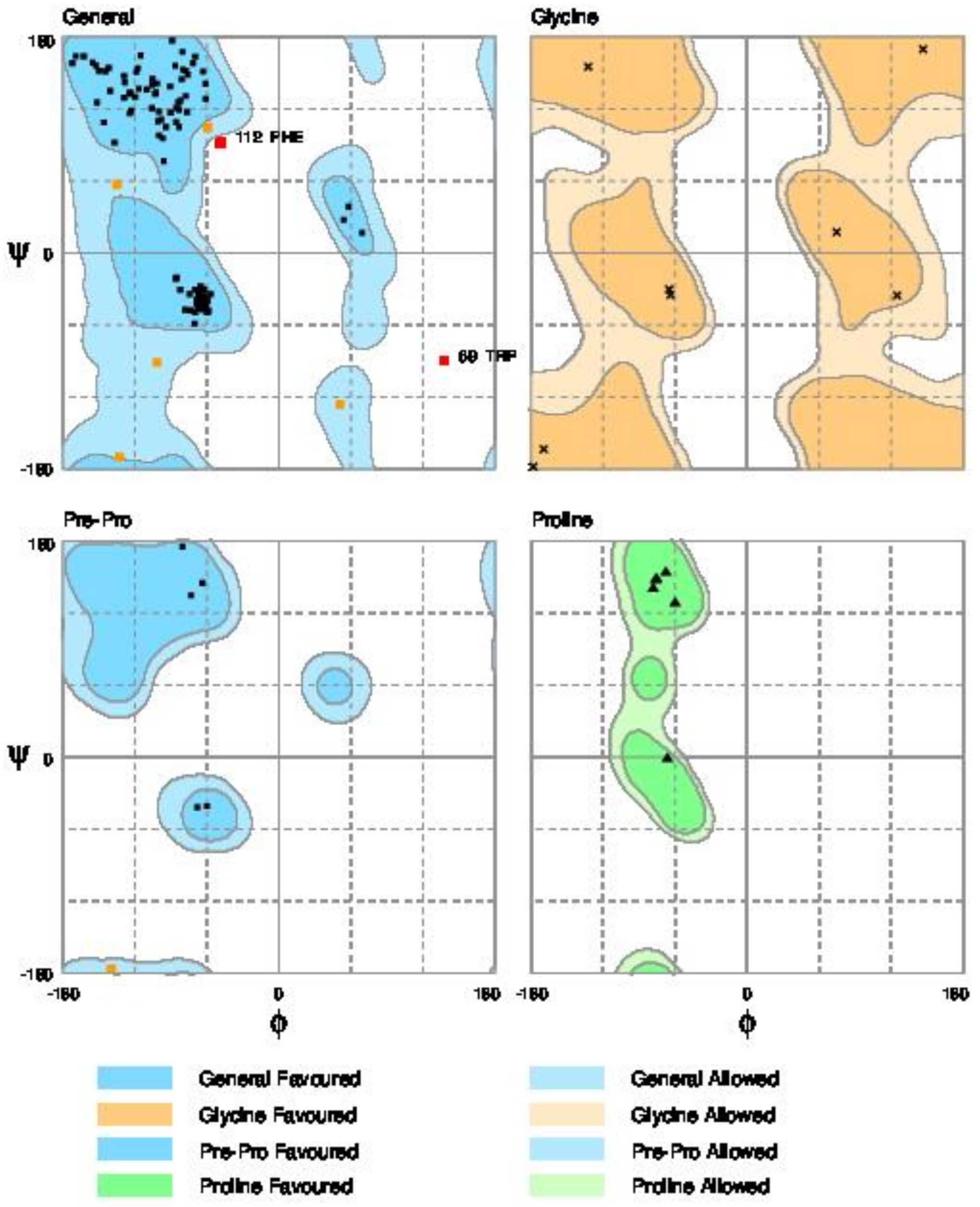

Evaluation of residues

```
Residue [   15 :ASN] (-139.88,-176.40) in Allowed region
Residue [   26 :PHE] (-133.08,-169.99) in Allowed region
Residue [   27 :GLU] ( -59.33, 104.55) in Allowed region
Residue [   71 :GLU] (  50.88,-126.14) in Allowed region
Residue [  124 :CYS] (-101.66, -91.24) in Allowed region
Residue [  126 :ASN] (-135.01,  57.06) in Allowed region
Residue [   69 :TRP] ( 137.92, -89.97) in Outlier region
Residue [  112 :PHE] ( -48.87,  91.83) in Outlier region
Number of residues in favoured region    (~98.0% expected)  :  147 ( 94.8%)
```

```
Number of residues in allowed region      ( ~2.0% expected)  :    6 (  3.9%)
Number of residues in outlier region                         :    2 (  1.3%)
```
