# Supplementary material for: Genome-wide survey and phylogeny of S-Ribosylhomocysteinase (LuxS) enzyme in bacterial genomes
Source: BMC Genomics. 2016 Sep 20;17:742. doi: 10.1186/s12864-016-3002-x (PMC5029033; doi:10.1186/s12864-016-3002-x)
Supplement: Additional file 10: — Homology models of LuxS of representatives from the clusters and Ramachandran plots of homology models. (ZIP 936 kb) [file 12864_2016_3002_MOESM10_ESM.zip › Additional_file_10/Lactobacillus_plantarum.pdf]

RAMPAGE: Assessment of the Ramachandran Plot

File: Lactobacillus_plantarum.pdb

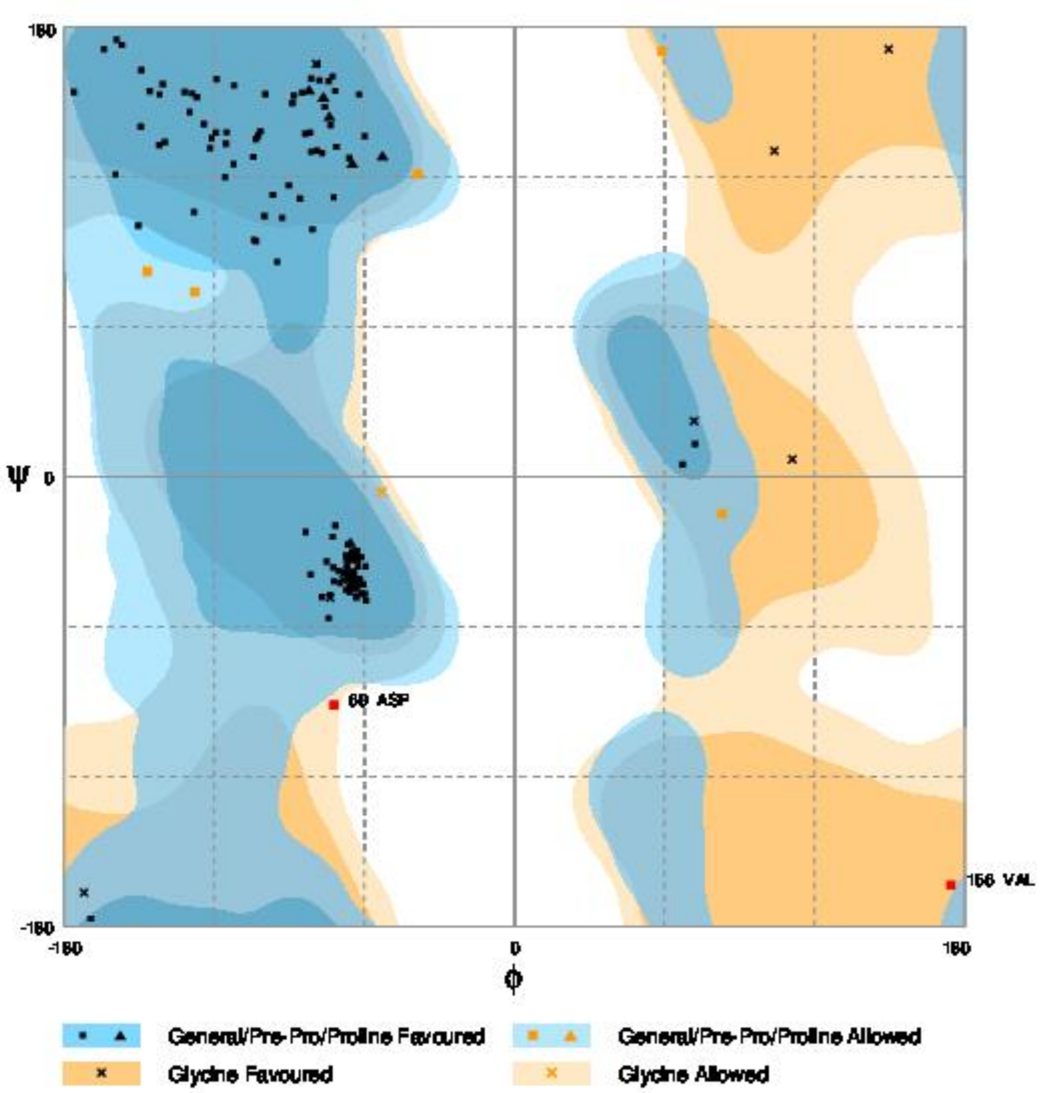

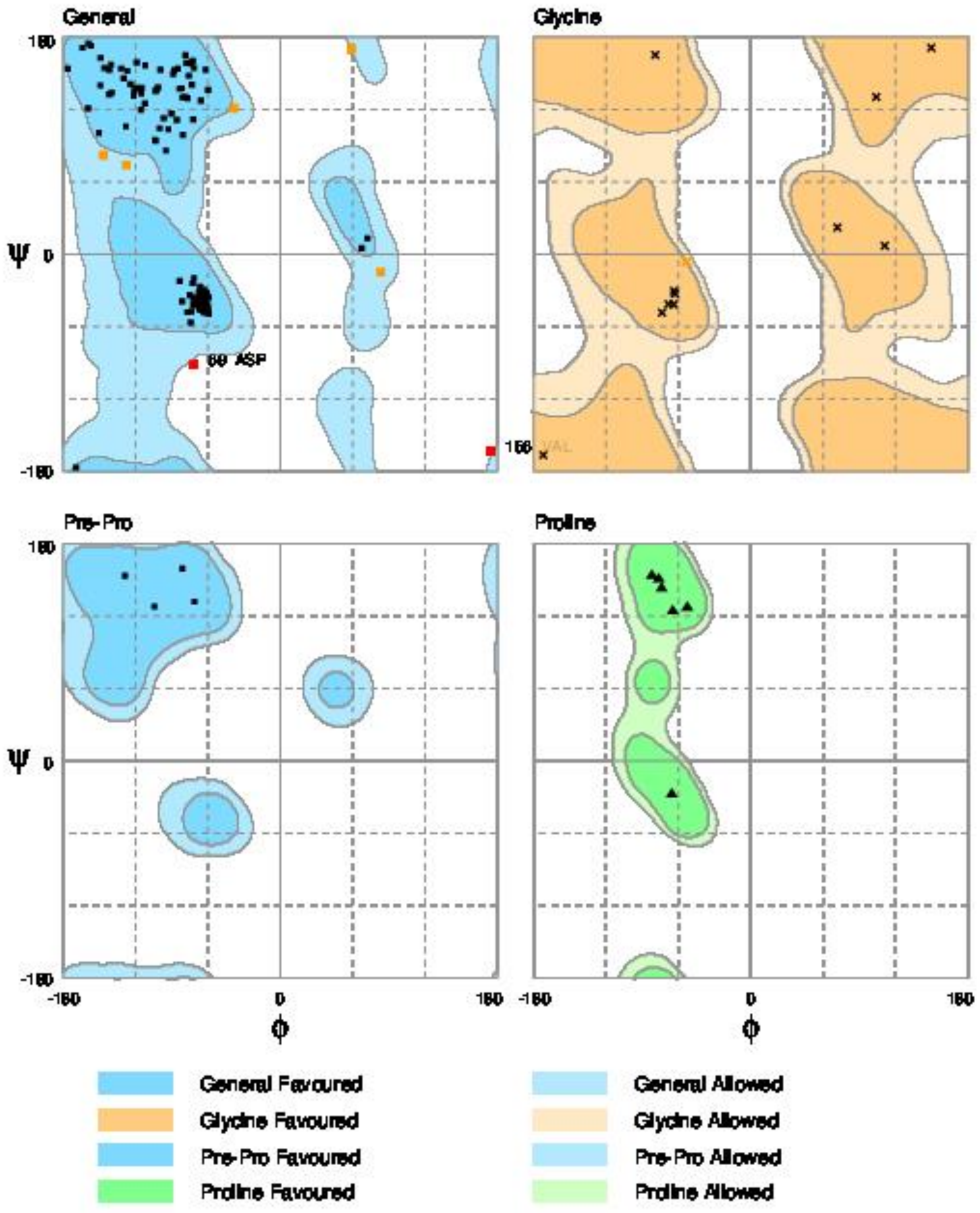

Evaluation of residues

```
Residue [   70 :GLY] ( -52.99,   -6.01) in Allowed region
Residue [   71 :VAL] ( -38.44, 121.08) in Allowed region
Residue [   92 :ASP] (-146.75,   82.08) in Allowed region
Residue [  112 :LYS] (  83.20, -14.83) in Allowed region
Residue [  126 :ASN] (-127.64,   74.03) in Allowed region
Residue [  154 :ARG] (  59.09, 170.22) in Allowed region
Residue [   69 :ASP] ( -72.06, -91.29) in Outlier region
Residue [  156 :VAL] ( 174.62,-163.40) in Outlier region
Number of residues in favoured region    (~98.0% expected)  :  148 ( 94.9%)
```

Number of residues in allowed region	(~2.0% expected)	:	6 (3.8%)
Number of residues in outlier region		:	2 (1.3%)
