# Supplementary material for: Genome-wide survey and phylogeny of S-Ribosylhomocysteinase (LuxS) enzyme in bacterial genomes
Source: BMC Genomics. 2016 Sep 20;17:742. doi: 10.1186/s12864-016-3002-x (PMC5029033; doi:10.1186/s12864-016-3002-x)
Supplement: Additional file 10: — Homology models of LuxS of representatives from the clusters and Ramachandran plots of homology models. (ZIP 936 kb) [file 12864_2016_3002_MOESM10_ESM.zip › Additional_file_10/Truepera_radiovictrix.pdf]

RAMPAGE: Assessment of the Ramachandran Plot

File: Truepera_radiovictrix.pdb

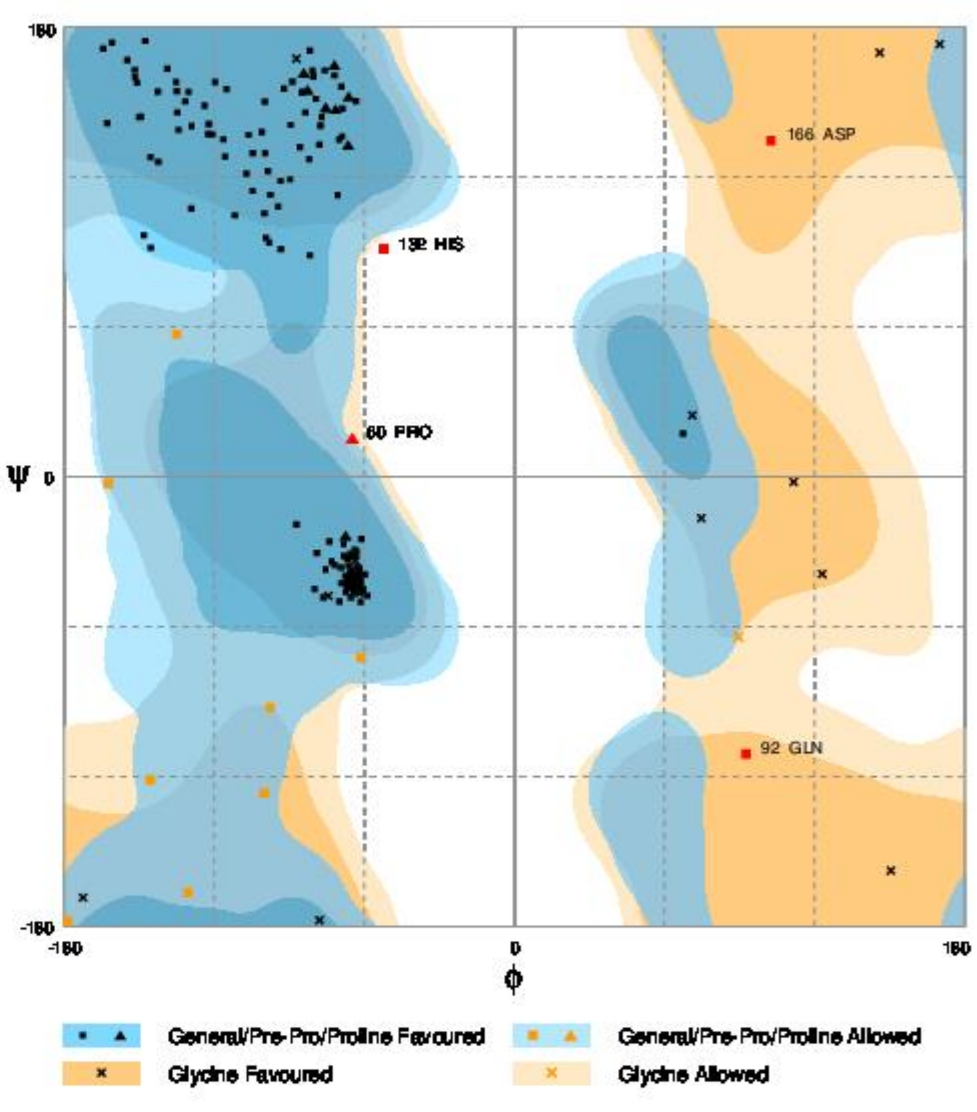

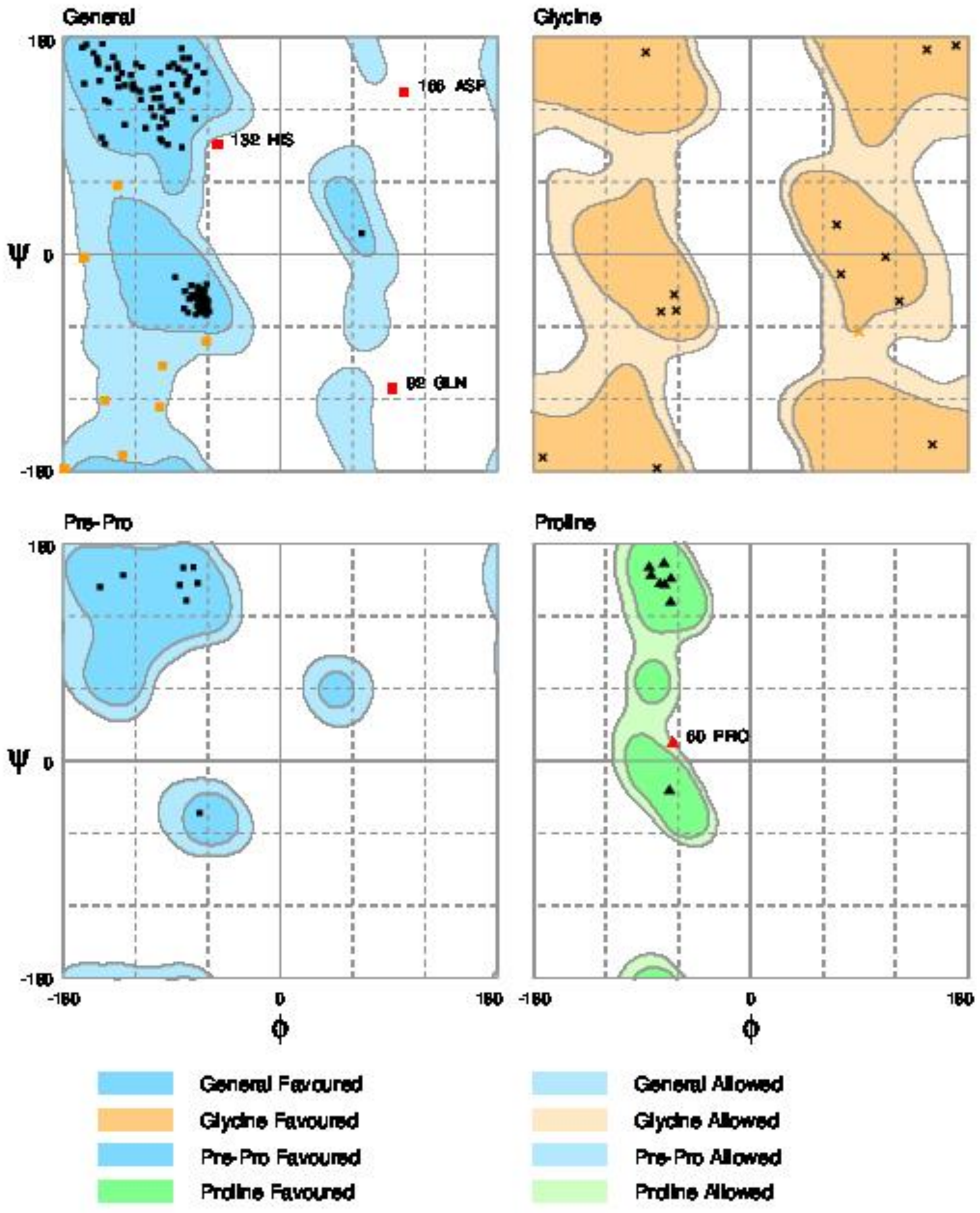

Evaluation of residues

```
Residue [   20 :ARG] (-145.46,-121.25) in Allowed region
Residue [   61 :ASN] (-162.26,   -2.66) in Allowed region
Residue [   63 :ALA] (-178.63,-177.84) in Allowed region
Residue [   86 :GLU] (-130.55,-166.43) in Allowed region
Residue [   88 :LEU] ( -61.43,  -72.39) in Allowed region
Residue [   93 :LEU] ( -99.94,-126.58) in Allowed region
Residue [  144 :CYS] ( -97.63,  -92.44) in Allowed region
Residue [  146 :ASN] (-134.97,   56.96) in Allowed region
Residue [  154 :GLY] (  89.66,  -64.20) in Allowed region
```

```
Residue [   60 :PRO] ( -64.72,  15.48) in Outlier region
Residue [   92 :GLN] (  92.73,-110.89) in Outlier region
Residue [  132 :HIS] ( -52.29,  91.01) in Outlier region
Residue [  166 :ASP] ( 102.58, 134.55) in Outlier region
Number of residues in favoured region    (~98.0% expected)  :  161 ( 92.5%)
Number of residues in allowed region     ( ~2.0% expected)  :    9 (  5.2%)
Number of residues in outlier region                        :    4 (  2.3%)
```
