# Supplementary material for: Genome-wide survey and phylogeny of S-Ribosylhomocysteinase (LuxS) enzyme in bacterial genomes
Source: BMC Genomics. 2016 Sep 20;17:742. doi: 10.1186/s12864-016-3002-x (PMC5029033; doi:10.1186/s12864-016-3002-x)
Supplement: Additional file 10: — Homology models of LuxS of representatives from the clusters and Ramachandran plots of homology models. (ZIP 936 kb) [file 12864_2016_3002_MOESM10_ESM.zip › Additional_file_10/Vibrio_harveyi.pdf]

RAMPAGE: Assessment of the Ramachandran Plot

File: Vibrio_harveyi.pdb

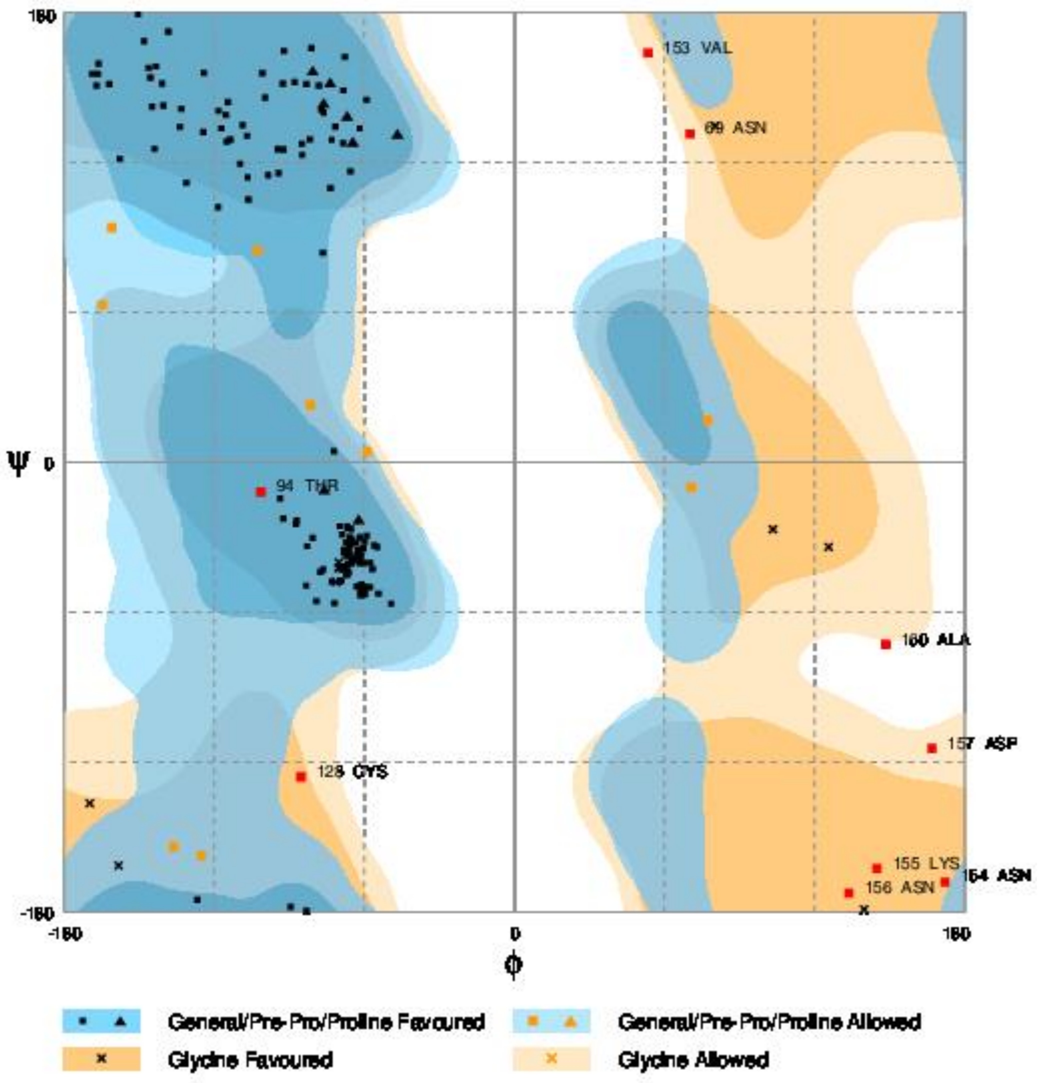

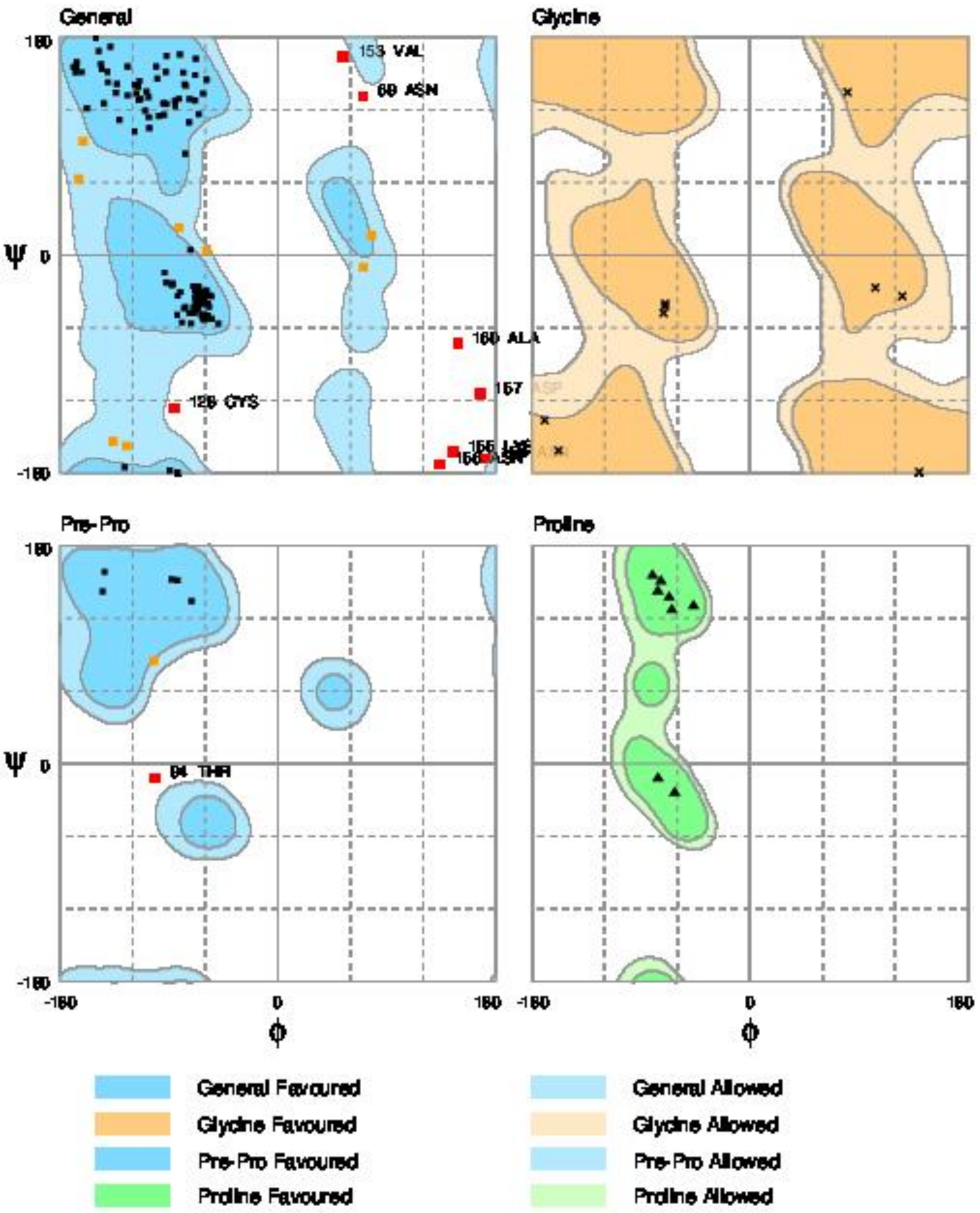

Evaluation of residues

```
Residue [    5 :ASP] (-136.17,-153.88) in Allowed region
Residue [   44 :ASN] (  70.68,   -9.99) in Allowed region
Residue [   84 :ARG] (  77.33,   16.67) in Allowed region
Residue [  118 :ASN] ( -81.54,   22.85) in Allowed region
Residue [  120 :ILE] (-102.58,   84.74) in Allowed region
Residue [  123 :LEU] ( -58.78,    4.06) in Allowed region
Residue [  130 :THR] (-164.66,   62.97) in Allowed region
Residue [  135 :SER] (-160.94,   93.95) in Allowed region
Residue [  158 :GLU] (-125.03,-157.52) in Allowed region
```

```
Residue [   69 :ASN] (  70.29, 131.38) in Outlier region
Residue [   94 :THR] (-101.33, -11.94) in Outlier region
Residue [  128 :CYS] ( -85.32,-125.93) in Outlier region
Residue [  153 :VAL] (  53.49, 163.80) in Outlier region
Residue [  154 :ASN] ( 172.16,-167.98) in Outlier region
Residue [  155 :LYS] ( 145.04,-162.64) in Outlier region
Residue [  156 :ASN] ( 133.84,-172.40) in Outlier region
Residue [  157 :ASP] ( 166.98,-114.52) in Outlier region
Residue [  160 :ALA] ( 148.40, -72.93) in Outlier region
Number of residues in favoured region    (~98.0% expected)  :  152 ( 89.4%)
Number of residues in allowed region     ( ~2.0% expected)  :    9 (  5.3%)
Number of residues in outlier region                        :    9 (  5.3%)
```
