# Supplementary material for: Genome-wide survey and phylogeny of S-Ribosylhomocysteinase (LuxS) enzyme in bacterial genomes
Source: BMC Genomics. 2016 Sep 20;17:742. doi: 10.1186/s12864-016-3002-x (PMC5029033; doi:10.1186/s12864-016-3002-x)
Supplement: Additional file 11: — Lesk-Hubbard plot of the multiple structural alignment of the homology models. (PDF 40 kb) [file 12864_2016_3002_MOESM11_ESM.pdf]

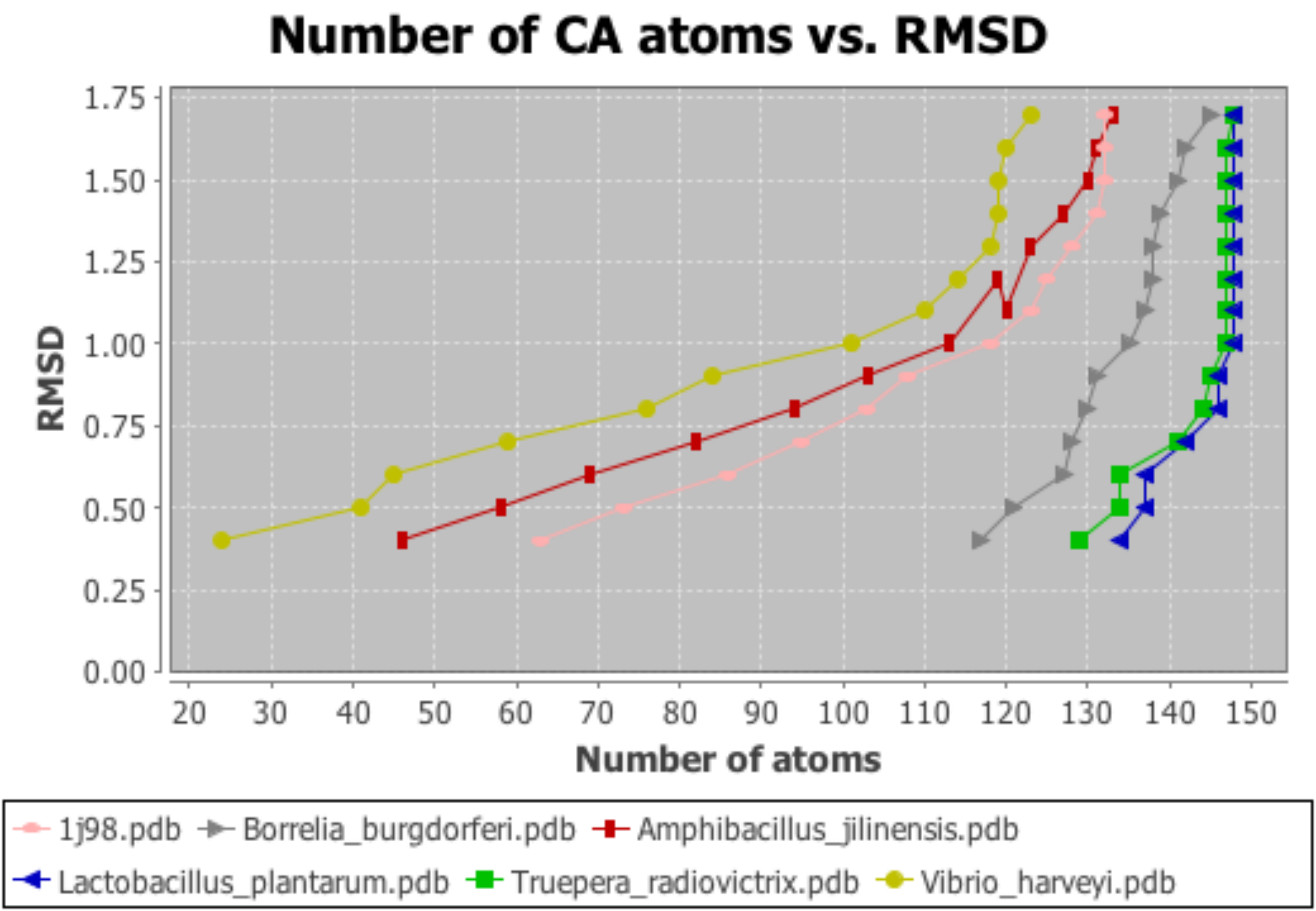

Number of CA atoms vs. RMSD
RMSD
1.75
1.50
1.25
1.00
0.75
0.50
0.25
0.00
20
30
40
50
60
70
80
90
100
110
120
130
140
150
Number of atoms
1j98.pdb
Borrelia_burgdorferi.pdb
Amphibacillus_jilinensis.pdb
Lactobacillus_plantarum.pdb
Truepera_radiovictrix.pdb
Vibrio_harveyi.pdb
