# Supplementary material for: Genome-wide survey and phylogeny of S-Ribosylhomocysteinase (LuxS) enzyme in bacterial genomes
Source: BMC Genomics. 2016 Sep 20;17:742. doi: 10.1186/s12864-016-3002-x (PMC5029033; doi:10.1186/s12864-016-3002-x)
Supplement: Additional file 12: — Multiple structure-based sequence alignment of homology models of LuxS protein sequences, showing surface electrostatic potentials. Residues labelled in red are regions with negative surface potentials; residues labelled in blue are regions having positive surface electrostatic potentials. (PDF 157 kb) [file 12864_2016_3002_MOESM12_ESM.pdf]

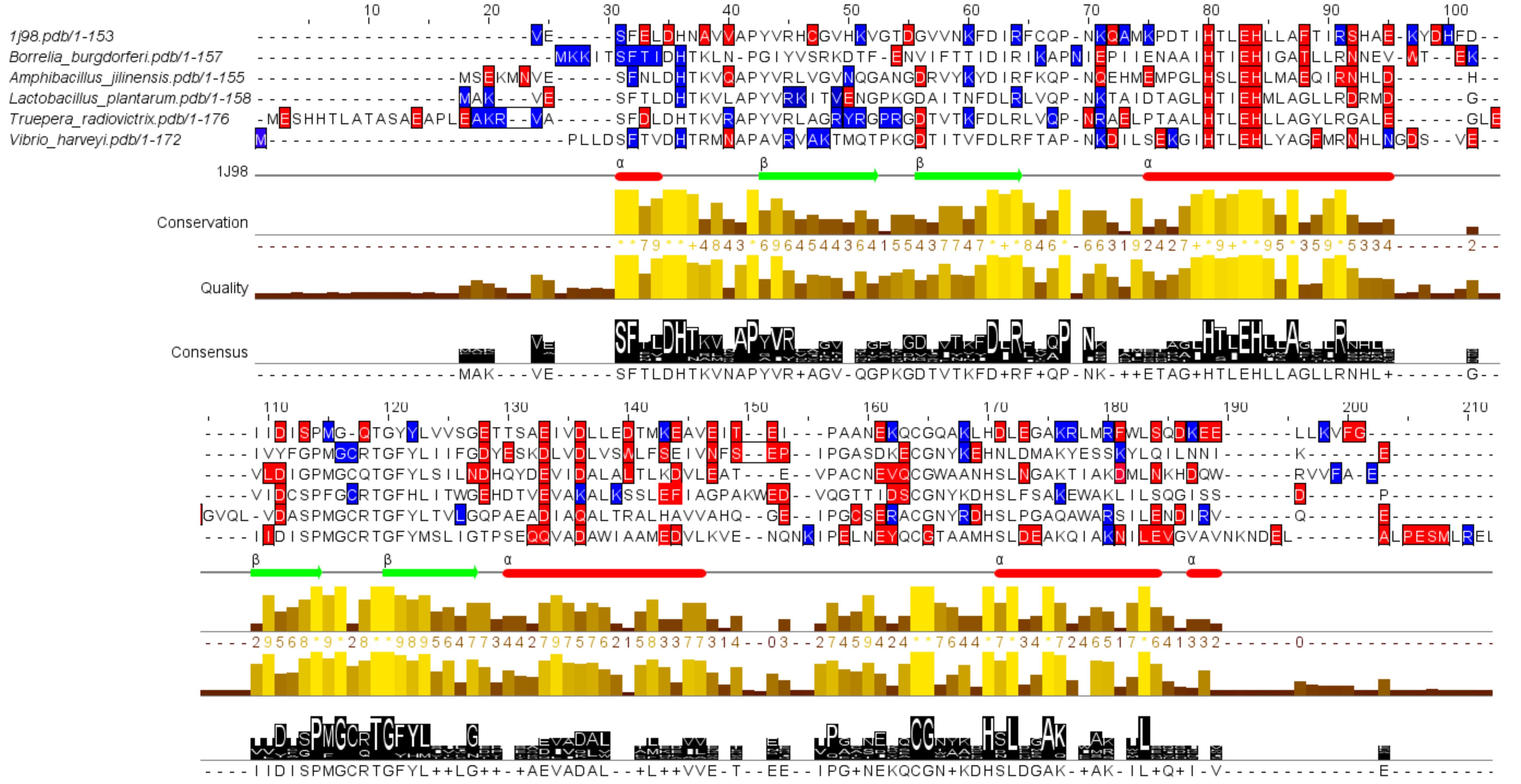

1j98.pdb/1-153
Borrelia_burgdorferi.pdb/1-157
Amphibacillus_jilinensis.pdb/1-155
Lactobacillus_plantarum.pdb/1-158
Truepera_radiovictrix.pdb/1-176
Vibrio_harveyi.pdb/1-172
1J98
Conservation
Quality
Consensus
