# Supplementary material for: Genome-wide survey and phylogeny of S-Ribosylhomocysteinase (LuxS) enzyme in bacterial genomes
Source: BMC Genomics. 2016 Sep 20;17:742. doi: 10.1186/s12864-016-3002-x (PMC5029033; doi:10.1186/s12864-016-3002-x)
Supplement: Additional file 5: — Phylogenetic tree of LuxS sequences showing habitat distribution of sequences. Colour codes: Pink: Gut/Faecal microorganisms; Purple: Animal and Human pathogens; Brown: Soil-dwelling bacteria; Yellow-green: Food-associated bacteria; Forest-green: Plant-associated bacteria; Sky blue: Halophiles; Navy blue: Alkaliphiles; Cyan: Psychrophiles; Orange: Thermophiles; Grey: Radiation-resistant bacteria; Yellow: Hydrothermal vent inhabiting bacteria; Lignt blue: Marine bacteria; Blue-green: Plant pathogens; Black: Habitat information not available (PDF 108 kb) [file 12864_2016_3002_MOESM5_ESM.pdf]

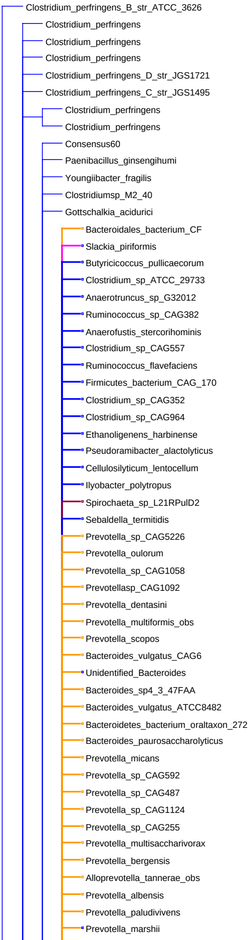
Clostridium_perfringens_B_str_ATCC_3626
Clostridium_perfringens
Clostridium_perfringens
Clostridium_perfringens
Clostridium_perfringens_D_str_JGS1721
Clostridium_perfringens_C_str_JGS1495
Clostridium_perfringens
Clostridium_perfringens
Consensus60
Paenibacillus_ginsengihumi
Youngiibacter_fragilis
Clostridiumsp_M2_40
Gottschalkia_acidurici
Bacteroidales_bacterium_CF
Slackia_piriformis
Butyricicoccus_pullicaecorum
Clostridium_sp_ATCC_29733
Anaerotruncus_sp_G32012
Ruminococcus_sp_CAG382
Anaerofustis_stercorihominis
Clostridium_sp_CAG557
Ruminococcus_flavefaciens
Firmicutes_bacterium_CAG_170
Clostridium_sp_CAG352
Clostridium_sp_CAG964
Ethanoligenens_harbinense
Pseudoramibacter_alactolyticus
Cellulosilyticum_lentocellum
Ilyobacter_polytropus
Spirochaeta_sp_L21RPulD2
Sebaldella_termitidis
Prevotella_sp_CAG5226
Prevotella_oulorum
Prevotella_sp_CAG1058
Prevotellasp_CAG1092
Prevotella_dentasini
Prevotella_multiformis_obs
Prevotella_scopos
Bacteroides_vulgatus_CAG6
Unidentified_Bacteroides
Bacteroides_sp4_3_47FAA
Bacteroides_vulgatus_ATCC8482
Bacteroidetes_bacterium_oraltaxon_272
Bacteroides_paurosaccharolyticus
Prevotella_micans
Prevotella_sp_CAG592
Prevotella_sp_CAG487
Prevotella_sp_CAG1124
Prevotella_sp_CAG255
Prevotella_multisaccharivorax
Prevotella_bergensis
Alloprevotella_tannerae_obs
Prevotella_albensis
Prevotella_paludivivens
Prevotella_marshii

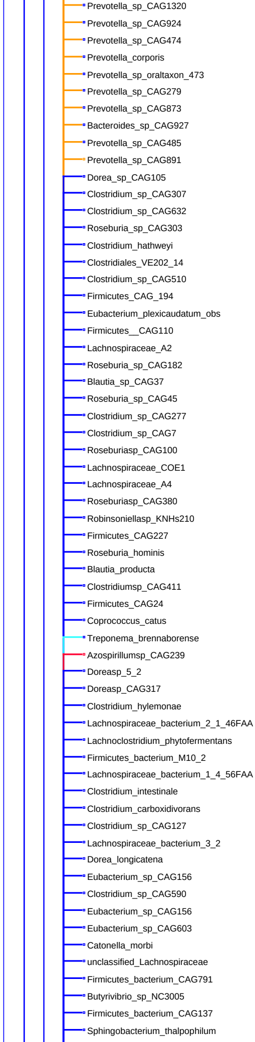
Prevotella_sp_CAG1320
Prevotella_sp_CAG924
Prevotella_sp_CAG474
Prevotella_corporis
Prevotella_sp_oraltaxon_473
Prevotella_sp_CAG279
Prevotella_sp_CAG873
Bacteroides_sp_CAG927
Prevotella_sp_CAG485
Prevotella_sp_CAG891
Dorea_sp_CAG105
Clostridium_sp_CAG307
Clostridium_sp_CAG632
Roseburia_sp_CAG303
Clostridium_hathweyi
Clostridiales_VE202_14
Clostridium_sp_CAG510
Firmicutes_CAG_194
Eubacterium_plexicaudatum_obs
Firmicutes__CAG110
Lachnospiraceae_A2
Roseburia_sp_CAG182
Blautia_sp_CAG37
Roseburia_sp_CAG45
Clostridium_sp_CAG277
Clostridium_sp_CAG7
Roseburiasp_CAG100
Lachnospiraceae_COE1
Lachnospiraceae_A4
Roseburiasp_CAG380
Robinsoniellasp_KNHs210
Firmicutes_CAG227
Roseburia_hominis
Blautia_producta
Clostridiumsp_CAG411
Firmicutes_CAG24
Coprococcus_catus
Treponema_brennaborense
Azospirillumsp_CAG239
Doreasp_5_2
Doreasp_CAG317
Clostridium_hylemonae
Lachnospiraceae_bacterium_2_1_46FAA
Lachnoclostridium_phytofermentans
Firmicutes_bacterium_M10_2
Lachnospiraceae_bacterium_1_4_56FAA
Clostridium_intestinale
Clostridium_carboxidivorans
Clostridium_sp_CAG127
Lachnospiraceae_bacterium_3_2
Dorea_longicatena
Eubacterium_sp_CAG156
Clostridium_sp_CAG590
Eubacterium_sp_CAG156
Eubacterium_sp_CAG603
Catonella_morbi
unclassified_Lachnospiraceae
Firmicutes_bacterium_CAG791
Butyrivibrio_sp_NC3005
Firmicutes_bacterium_CAG137
Sphingobacterium_thalpophilum

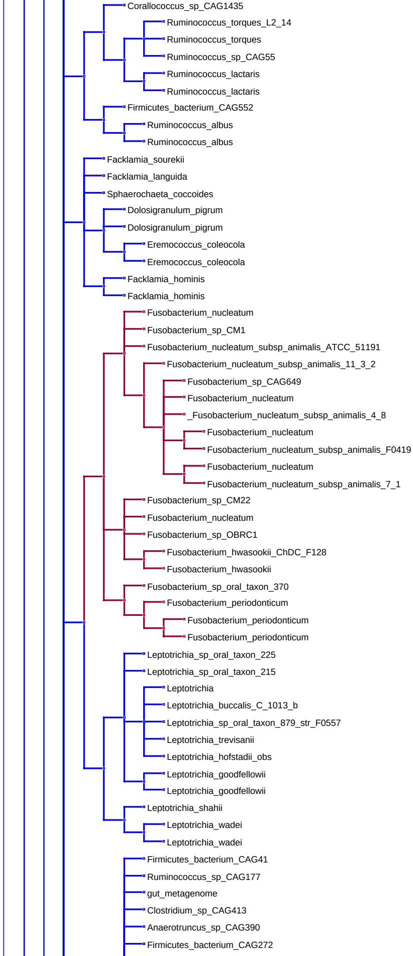
Corallococcus_sp_CAG1435
Ruminococcus_torques_L2_14
Ruminococcus_torques
Ruminococcus_sp_CAG55
Ruminococcus_lactaris
Ruminococcus_lactaris
Firmicutes_bacterium_CAG552
Ruminococcus_albus
Ruminococcus_albus
Facklamia_sourekii
Facklamia_languida
Sphaerochaeta_coccoides
Dolosigranulum_pigrum
Dolosigranulum_pigrum
Eremococcus_coleocola
Eremococcus_coleocola
Facklamia_hominis
Facklamia_hominis
Fusobacterium_nucleatum
Fusobacterium_sp_CM1
Fusobacterium_nucleatum_subsp_animalis_ATCC_51191
Fusobacterium_nucleatum_subsp_animalis_11_3_2
Fusobacterium_sp_CAG649
Fusobacterium_nucleatum
_Fusobacterium_nucleatum_subsp_animalis_4_8
Fusobacterium_nucleatum
Fusobacterium_nucleatum_subsp_animalis_F0419
Fusobacterium_nucleatum
Fusobacterium_nucleatum_subsp_animalis_7_1
Fusobacterium_sp_CM22
Fusobacterium_nucleatum
Fusobacterium_sp_OBRC1
Fusobacterium_hwasookii_ChDC_F128
Fusobacterium_hwasookii
Fusobacterium_sp_oral_taxon_370
Fusobacterium_periodonticum
Fusobacterium_periodonticum
Fusobacterium_periodonticum
Leptotrichia_sp_oral_taxon_225
Leptotrichia_sp_oral_taxon_215
Leptotrichia
Leptotrichia_buccalis_C_1013_b
Leptotrichia_sp_oral_taxon_879_str_F0557
Leptotrichia_trevisanii
Leptotrichia_hofstadii_obs
Leptotrichia_goodfellowii
Leptotrichia_goodfellowii
Leptotrichia_shahii
Leptotrichia_wadei
Leptotrichia_wadei
Firmicutes_bacterium_CAG41
Ruminococcus_sp_CAG177
gut_metagenome
Clostridium_sp_CAG413
Anaerotruncus_sp_CAG390
Firmicutes_bacterium_CAG272

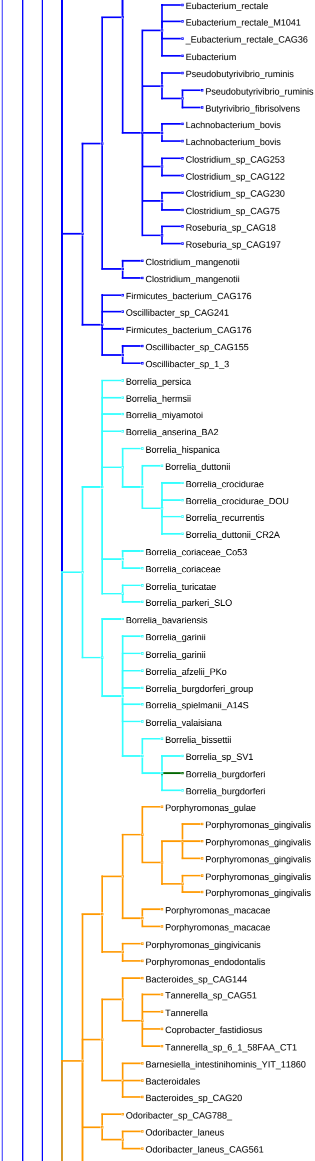

Eubacterium_rectale
Eubacterium_rectale_M1041
_Eubacterium_rectale_CAG36
Eubacterium
Pseudobutyrivibrio_ruminis
Pseudobutyrivibrio_ruminis
Butyrivibrio_fibrisolvens
Lachnobacterium_bovis
Lachnobacterium_bovis
Clostridium_sp_CAG253
Clostridium_sp_CAG122
Clostridium_sp_CAG230
Clostridium_sp_CAG75
Roseburia_sp_CAG18
Roseburia_sp_CAG197
Clostridium_mangenotii
Clostridium_mangenotii
Firmicutes_bacterium_CAG176
Oscillibacter_sp_CAG241
Firmicutes_bacterium_CAG176
Oscillibacter_sp_CAG155
Oscillibacter_sp_1_3
Borrelia_persica
Borrelia_hermsii
Borrelia_miyamotoi
Borrelia_anserina_BA2
Borrelia_hispanica
Borrelia_duttonii
Borrelia_crocidurae
Borrelia_crocidurae_DOU
Borrelia_recurrentis
Borrelia_duttonii_CR2A
Borrelia_coriaceae_Co53
Borrelia_coriaceae
Borrelia_turicatae
Borrelia_parkeri_SLO
Borrelia_bavariensis
Borrelia_garinii
Borrelia_garinii
Borrelia_afzelii_PKo
Borrelia_burgdorferi_group
Borrelia_spielmanii_A14S
Borrelia_valaisiana
Borrelia_bissettii
Borrelia_sp_SV1
Borrelia_burgdorferi
Borrelia_burgdorferi
Porphyromonas_gulae
Porphyromonas_gingivalis
Porphyromonas_gingivalis
Porphyromonas_gingivalis
Porphyromonas_gingivalis
Porphyromonas_gingivalis
Porphyromonas_macacae
Porphyromonas_macacae
Porphyromonas_gingivicanis
Porphyromonas_endodontalis
Bacteroides_sp_CAG144
Tannerella_sp_CAG51
Tannerella
Coprobacter_fastidiosus
Tannerella_sp_6_1_58FAA_CT1
Barnesiella_intestinihominis_YIT_11860
Bacteroidales
Bacteroides_sp_CAG20
Odoribacter_sp_CAG788_
Odoribacter_laneus
Odoribacter_laneus_CAG561

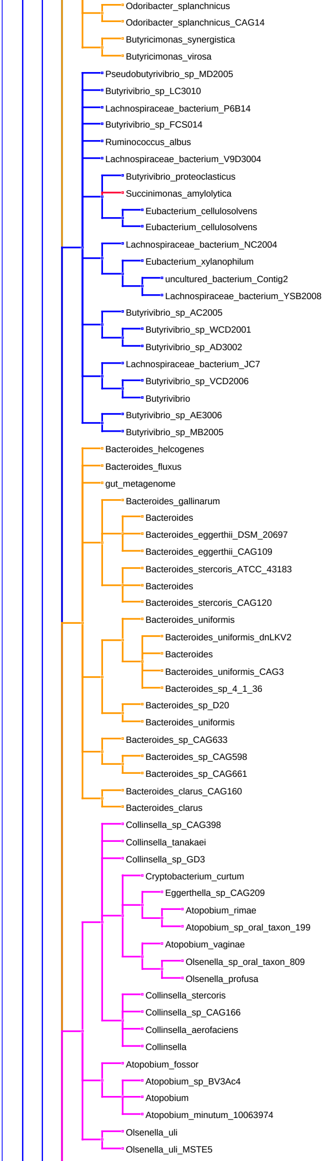

Odoribacter_splanchnicus
Odoribacter_splanchnicus_CAG14
Butyricimonas_synergistica
Butyricimonas_virosa
Pseudobutyrivibrio_sp_MD2005
Butyrivibrio_sp_LC3010
Lachnospiraceae_bacterium_P6B14
Butyrivibrio_sp_FCS014
Ruminococcus_albus
Lachnospiraceae_bacterium_V9D3004
Butyrivibrio_proteoclasticus
Succinimonas_amylolytica
Eubacterium_cellulosolvens
Eubacterium_cellulosolvens
Lachnospiraceae_bacterium_NC2004
Eubacterium_xylanophilum
uncultured_bacterium_Contig2
Lachnospiraceae_bacterium_YSB2008
Butyrivibrio_sp_AC2005
Butyrivibrio_sp_WCD2001
Butyrivibrio_sp_AD3002
Lachnospiraceae_bacterium_JC7
Butyrivibrio_sp_VCD2006
Butyrivibrio
Butyrivibrio_sp_AE3006
Butyrivibrio_sp_MB2005
Bacteroides_helcogenes
Bacteroides_fluxus
gut_metagenome
Bacteroides_gallinarum
Bacteroides
Bacteroides_eggerthii_DSM_20697
Bacteroides_eggerthii_CAG109
Bacteroides_stercoris_ATCC_43183
Bacteroides
Bacteroides_stercoris_CAG120
Bacteroides_uniformis
Bacteroides_uniformis_dnLKV2
Bacteroides
Bacteroides_uniformis_CAG3
Bacteroides_sp_4_1_36
Bacteroides_sp_D20
Bacteroides_uniformis
Bacteroides_sp_CAG633
Bacteroides_sp_CAG598
Bacteroides_sp_CAG661
Bacteroides_clarus_CAG160
Bacteroides_clarus
Collinsella_sp_CAG398
Collinsella_tanakaei
Collinsella_sp_GD3
Cryptobacterium_curtum
Eggerthella_sp_CAG209
Atopobium_rimae
Atopobium_sp_oral_taxon_199
Atopobium_vaginae
Olsenella_sp_oral_taxon_809
Olsenella_profusa
Collinsella_stercoris
Collinsella_sp_CAG166
Collinsella_aerofaciens
Collinsella
Atopobium_fossor
Atopobium_sp_BV3Ac4
Atopobium
Atopobium_minutum_10063974
Olsenella_uli
Olsenella_uli_MSTE5

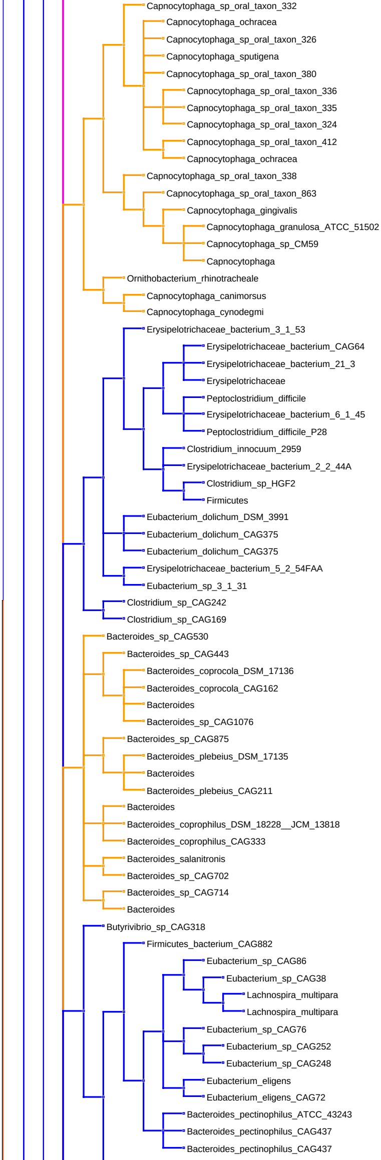
Capnocytophaga_sp_oral_taxon_332
Capnocytophaga_ochracea
Capnocytophaga_sp_oral_taxon_326
Capnocytophaga_sputigena
Capnocytophaga_sp_oral_taxon_380
Capnocytophaga_sp_oral_taxon_336
Capnocytophaga_sp_oral_taxon_335
Capnocytophaga_sp_oral_taxon_324
Capnocytophaga_sp_oral_taxon_412
Capnocytophaga_ochracea
Capnocytophaga_sp_oral_taxon_338
Capnocytophaga_sp_oral_taxon_863
Capnocytophaga_gingivalis
Capnocytophaga_granulosa_ATCC_51502
Capnocytophaga_sp_CM59
Capnocytophaga
Ornithobacterium_rhinotracheale
Capnocytophaga_canimorsus
Capnocytophaga_cynodegmi
Erysipelotrichaceae_bacterium_3_1_53
Erysipelotrichaceae_bacterium_CAG64
Erysipelotrichaceae_bacterium_21_3
Erysipelotrichaceae
Peptoclostridium_difficile
Erysipelotrichaceae_bacterium_6_1_45
Peptoclostridium_difficile_P28
Clostridium_innocuum_2959
Erysipelotrichaceae_bacterium_2_2_44A
Clostridium_sp_HGF2
Firmicutes
Eubacterium_dolichum_DSM_3991
Eubacterium_dolichum_CAG375
Eubacterium_dolichum_CAG375
Erysipelotrichaceae_bacterium_5_2_54FAA
Eubacterium_sp_3_1_31
Clostridium_sp_CAG242
Clostridium_sp_CAG169
Bacteroides_sp_CAG530
Bacteroides_sp_CAG443
Bacteroides_coprocola_DSM_17136
Bacteroides_coprocola_CAG162
Bacteroides
Bacteroides_sp_CAG1076
Bacteroides_sp_CAG875
Bacteroides_plebeius_DSM_17135
Bacteroides
Bacteroides_plebeius_CAG211
Bacteroides
Bacteroides_coprophilus_DSM_18228__JCM_13818
Bacteroides_coprophilus_CAG333
Bacteroides_salanitronis
Bacteroides_sp_CAG702
Bacteroides_sp_CAG714
Bacteroides
Butyrivibrio_sp_CAG318
Firmicutes_bacterium_CAG882
Eubacterium_sp_CAG86
Eubacterium_sp_CAG38
Lachnospira_multipara
Lachnospira_multipara
Eubacterium_sp_CAG76
Eubacterium_sp_CAG252
Eubacterium_sp_CAG248
Eubacterium_eligens
Eubacterium_eligens_CAG72
Bacteroides_pectinophilus_ATCC_43243
Bacteroides_pectinophilus_CAG437
Bacteroides_pectinophilus_CAG437

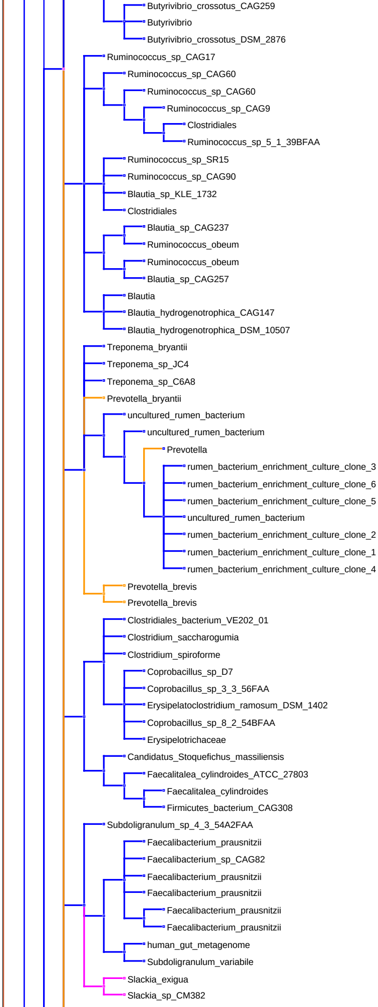
Butyrivibrio_crossotus_CAG259
Butyrivibrio
Butyrivibrio_crossotus_DSM_2876
Ruminococcus_sp_CAG17
Ruminococcus_sp_CAG60
Ruminococcus_sp_CAG60
Ruminococcus_sp_CAG9
Clostridiales
Ruminococcus_sp_5_1_39BFAA
Ruminococcus_sp_SR15
Ruminococcus_sp_CAG90
Blautia_sp_KLE_1732
Clostridiales
Blautia_sp_CAG237
Ruminococcus_obeum
Ruminococcus_obeum
Blautia_sp_CAG257
Blautia
Blautia_hydrogenotrophica_CAG147
Blautia_hydrogenotrophica_DSM_10507
Treponema_bryantii
Treponema_sp_JC4
Treponema_sp_C6A8
Prevotella_bryantii
uncultured_rumen_bacterium
uncultured_rumen_bacterium
Prevotella
rumen_bacterium_enrichment_culture_clone_3
rumen_bacterium_enrichment_culture_clone_6
rumen_bacterium_enrichment_culture_clone_5
uncultured_rumen_bacterium
rumen_bacterium_enrichment_culture_clone_2
rumen_bacterium_enrichment_culture_clone_1
rumen_bacterium_enrichment_culture_clone_4
Prevotella_brevis
Prevotella_brevis
Clostridiales_bacterium_VE202_01
Clostridium_saccharogumia
Clostridium_spiroforme
Coprobacillus_sp_D7
Coprobacillus_sp_3_3_56FAA
Erysipelatoclostridium_ramosum_DSM_1402
Coprobacillus_sp_8_2_54BFAA
Erysipelotrichaceae
Candidatus_Stoquefichus_massiliensis
Faecalitalea_cylindroides_ATCC_27803
Faecalitalea_cylindroides
Firmicutes_bacterium_CAG308
Subdoligranulum_sp_4_3_54A2FAA
Faecalibacterium_prausnitzii
Faecalibacterium_sp_CAG82
Faecalibacterium_prausnitzii
Faecalibacterium_prausnitzii
Faecalibacterium_prausnitzii
Faecalibacterium_prausnitzii
human_gut_metagenome
Subdoligranulum_variabile
Slackia_exigua
Slackia_sp_CM382

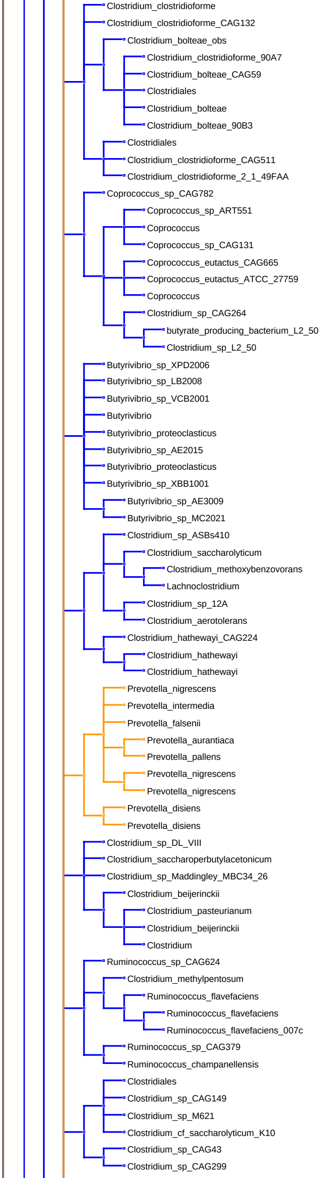
Clostridium_clostridioforme
Clostridium_clostridioforme_CAG132
Clostridium_bolteae_obs
Clostridium_clostridioforme_90A7
Clostridium_bolteae_CAG59
Clostridiales
Clostridium_bolteae
Clostridium_bolteae_90B3
Clostridiales
Clostridium_clostridioforme_CAG511
Clostridium_clostridioforme_2_1_49FAA
Coprococcus_sp_CAG782
Coprococcus_sp_ART551
Coprococcus
Coprococcus_sp_CAG131
Coprococcus_eutactus_CAG665
Coprococcus_eutactus_ATCC_27759
Coprococcus
Clostridium_sp_CAG264
butyrate_producing_bacterium_L2_50
Clostridium_sp_L2_50
Butyrivibrio_sp_XPD2006
Butyrivibrio_sp_LB2008
Butyrivibrio_sp_VCB2001
Butyrivibrio
Butyrivibrio_proteoclasticus
Butyrivibrio_sp_AE2015
Butyrivibrio_proteoclasticus
Butyrivibrio_sp_XBB1001
Butyrivibrio_sp_AE3009
Butyrivibrio_sp_MC2021
Clostridium_sp_ASBs410
Clostridium_saccharolyticum
Clostridium_methoxybenzovorans
Lachnoclostridium
Clostridium_sp_12A
Clostridium_aerotolerans
Clostridium_hathewayi_CAG224
Clostridium_hathewayi
Clostridium_hathewayi
Prevotella_nigrescens
Prevotella_intermedia
Prevotella_falsenii
Prevotella_aurantiaca
Prevotella_pallens
Prevotella_nigrescens
Prevotella_nigrescens
Prevotella_disiens
Prevotella_disiens
Clostridium_sp_DL_VIII
Clostridium_saccharoperbutylacetonicum
Clostridium_sp_Maddingley_MBC34_26
Clostridium_beijerinckii
Clostridium_pasteurianum
Clostridium_beijerinckii
Clostridium
Ruminococcus_sp_CAG624
Clostridium_methylpentosum
Ruminococcus_flavefaciens
Ruminococcus_flavefaciens
Ruminococcus_flavefaciens_007c
Ruminococcus_sp_CAG379
Ruminococcus_champanellensis
Clostridiales
Clostridium_sp_CAG149
Clostridium_sp_M621
Clostridium_cf_saccharolyticum_K10
Clostridium_sp_CAG43
Clostridium_sp_CAG299

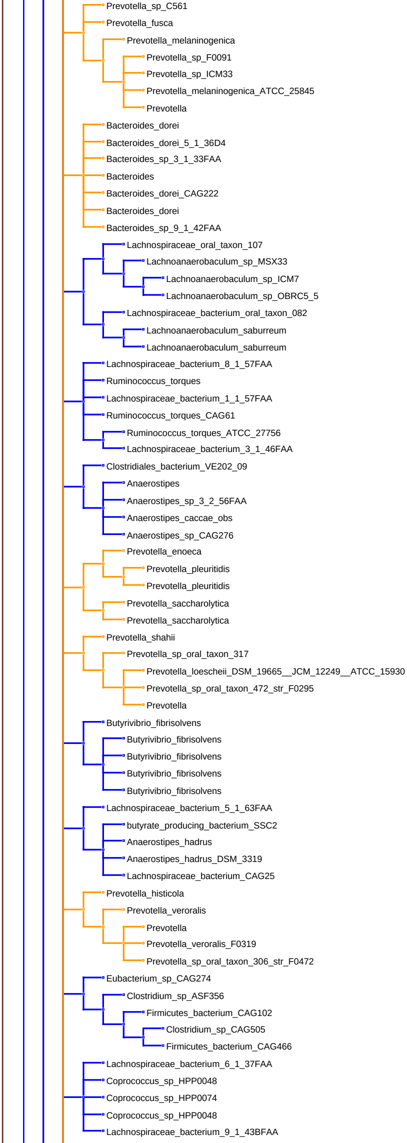
Prevotella_sp_C561
Prevotella_fusca
Prevotella_melaninogenica
Prevotella_sp_F0091
Prevotella_sp_ICM33
Prevotella_melaninogenica_ATCC_25845
Prevotella
Bacteroides_dorei
Bacteroides_dorei_5_1_36D4
Bacteroides_sp_3_1_33FAA
Bacteroides
Bacteroides_dorei_CAG222
Bacteroides_dorei
Bacteroides_sp_9_1_42FAA
Lachnospiraceae_oral_taxon_107
Lachnoanaerobaculum_sp_MSX33
Lachnoanaerobaculum_sp_ICM7
Lachnoanaerobaculum_sp_OBRC5_5
Lachnospiraceae_bacterium_oral_taxon_082
Lachnoanaerobaculum_saburreum
Lachnoanaerobaculum_saburreum
Lachnospiraceae_bacterium_8_1_57FAA
Ruminococcus_torques
Lachnospiraceae_bacterium_1_1_57FAA
Ruminococcus_torques_CAG61
Ruminococcus_torques_ATCC_27756
Lachnospiraceae_bacterium_3_1_46FAA
Clostridiales_bacterium_VE202_09
Anaerostipes
Anaerostipes_sp_3_2_56FAA
Anaerostipes_caccae_obs
Anaerostipes_sp_CAG276
Prevotella_enoeca
Prevotella_pleuritidis
Prevotella_pleuritidis
Prevotella_saccharolytica
Prevotella_saccharolytica
Prevotella_shahii
Prevotella_sp_oral_taxon_317
Prevotella_loescheii_DSM_19665__JCM_12249__ATCC_15930
Prevotella_sp_oral_taxon_472_str_F0295
Prevotella
Butyrivibrio_fibrisolvens
Butyrivibrio_fibrisolvens
Butyrivibrio_fibrisolvens
Butyrivibrio_fibrisolvens
Butyrivibrio_fibrisolvens
Lachnospiraceae_bacterium_5_1_63FAA
butyrate_producing_bacterium_SSC2
Anaerostipes_hadrus
Anaerostipes_hadrus_DSM_3319
Lachnospiraceae_bacterium_CAG25
Prevotella_histicola
Prevotella_veroralis
Prevotella
Prevotella_veroralis_F0319
Prevotella_sp_oral_taxon_306_str_F0472
Eubacterium_sp_CAG274
Clostridium_sp_ASF356
Firmicutes_bacterium_CAG102
Clostridium_sp_CAG505
Firmicutes_bacterium_CAG466
Lachnospiraceae_bacterium_6_1_37FAA
Coprococcus_sp_HPP0048
Coprococcus_sp_HPP0074
Coprococcus_sp_HPP0048
Lachnospiraceae_bacterium_9_1_43BFAA

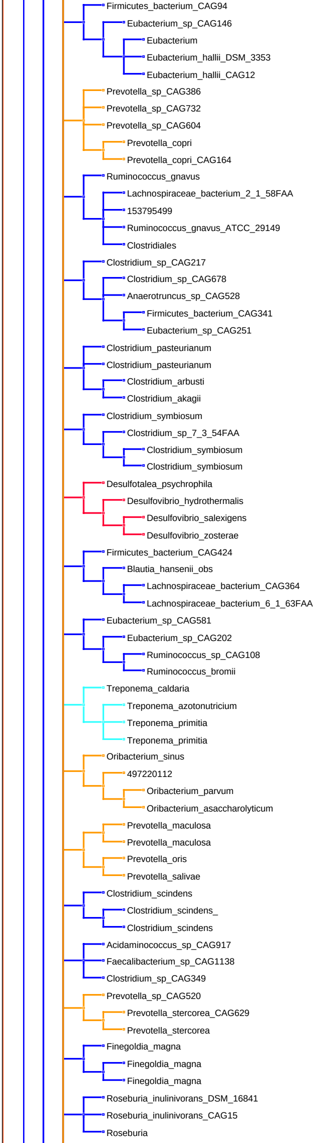
Firmicutes_bacterium_CAG94
Eubacterium_sp_CAG146
Eubacterium
Eubacterium_hallii_DSM_3353
Eubacterium_hallii_CAG12
Prevotella_sp_CAG386
Prevotella_sp_CAG732
Prevotella_sp_CAG604
Prevotella_copri
Prevotella_copri_CAG164
Ruminococcus_gnavus
Lachnospiraceae_bacterium_2_1_58FAA
153795499
Ruminococcus_gnavus_ATCC_29149
Clostridiales
Clostridium_sp_CAG217
Clostridium_sp_CAG678
Anaerotruncus_sp_CAG528
Firmicutes_bacterium_CAG341
Eubacterium_sp_CAG251
Clostridium_pasteurianum
Clostridium_pasteurianum
Clostridium_arbusti
Clostridium_akagii
Clostridium_symbiosum
Clostridium_sp_7_3_54FAA
Clostridium_symbiosum
Clostridium_symbiosum
Desulfotalea_psychrophila
Desulfovibrio_hydrothermalis
Desulfovibrio_salexigens
Desulfovibrio_zosterae
Firmicutes_bacterium_CAG424
Blautia_hansenii_obs
Lachnospiraceae_bacterium_CAG364
Lachnospiraceae_bacterium_6_1_63FAA
Eubacterium_sp_CAG581
Eubacterium_sp_CAG202
Ruminococcus_sp_CAG108
Ruminococcus_bromii
Treponema_caldaria
Treponema_azotonutricium
Treponema_primitia
Treponema_primitia
Oribacterium_sinus
497220112
Oribacterium_parvum
Oribacterium_asaccharolyticum
Prevotella_maculosa
Prevotella_maculosa
Prevotella_oris
Prevotella_salivae
Clostridium_scindens
Clostridium_scindens_
Clostridium_scindens
Acidaminococcus_sp_CAG917
Faecalibacterium_sp_CAG1138
Clostridium_sp_CAG349
Prevotella_sp_CAG520
Prevotella_stercorea_CAG629
Prevotella_stercorea
Finegoldia_magna
Finegoldia_magna
Finegoldia_magna
Roseburia_inulinivorans_DSM_16841
Roseburia_inulinivorans_CAG15
Roseburia

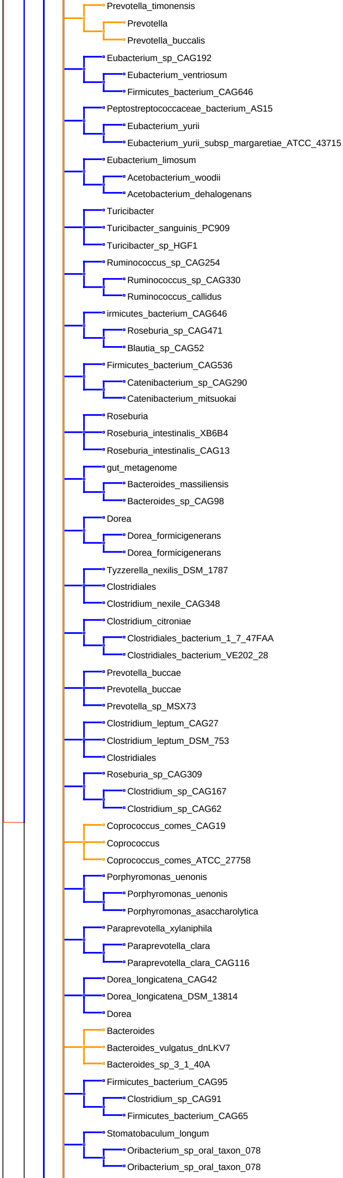
Prevotella_timonensis
Prevotella
Prevotella_buccalis
Eubacterium_sp_CAG192
Eubacterium_ventriosum
Firmicutes_bacterium_CAG646
Peptostreptococcaceae_bacterium_AS15
Eubacterium_yurii
Eubacterium_yurii_subsp_margaretiae_ATCC_43715
Eubacterium_limosum
Acetobacterium_woodii
Acetobacterium_dehalogenans
Turicibacter
Turicibacter_sanguinis_PC909
Turicibacter_sp_HGF1
Ruminococcus_sp_CAG254
Ruminococcus_sp_CAG330
Ruminococcus_callidus
irmicutes_bacterium_CAG646
Roseburia_sp_CAG471
Blautia_sp_CAG52
Firmicutes_bacterium_CAG536
Catenibacterium_sp_CAG290
Catenibacterium_mitsuokai
Roseburia
Roseburia_intestinalis_XB6B4
Roseburia_intestinalis_CAG13
gut_metagenome
Bacteroides_massiliensis
Bacteroides_sp_CAG98
Dorea
Dorea_formicigenerans
Dorea_formicigenerans
Tyzzerella_nexilis_DSM_1787
Clostridiales
Clostridium_nexile_CAG348
Clostridium_citroniae
Clostridiales_bacterium_1_7_47FAA
Clostridiales_bacterium_VE202_28
Prevotella_buccae
Prevotella_buccae
Prevotella_sp_MSX73
Clostridium_leptum_CAG27
Clostridium_leptum_DSM_753
Clostridiales
Roseburia_sp_CAG309
Clostridium_sp_CAG167
Clostridium_sp_CAG62
Coprococcus_comes_CAG19
Coprococcus
Coprococcus_comes_ATCC_27758
Porphyromonas_uenonis
Porphyromonas_uenonis
Porphyromonas_asaccharolytica
Paraprevotella_xylaniphila
Paraprevotella_clara
Paraprevotella_clara_CAG116
Dorea_longicatena_CAG42
Dorea_longicatena_DSM_13814
Dorea
Bacteroides
Bacteroides_vulgatus_dnLKV7
Bacteroides_sp_3_1_40A
Firmicutes_bacterium_CAG95
Clostridium_sp_CAG91
Firmicutes_bacterium_CAG65
Stomatobaculum_longum
Oribacterium_sp_oral_taxon_078
Oribacterium_sp_oral_taxon_078

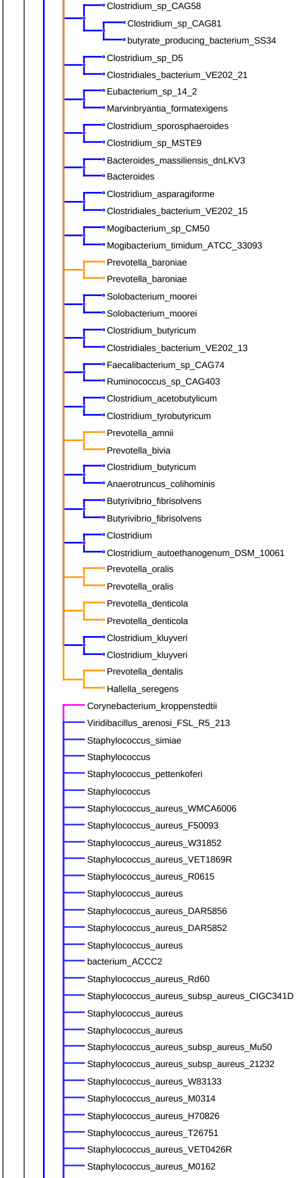
Clostridium_sp_CAG58
Clostridium_sp_CAG81
butyrate_producing_bacterium_SS34
Clostridium_sp_D5
Clostridiales_bacterium_VE202_21
Eubacterium_sp_14_2
Marvinbryantia_formatexigens
Clostridium_sporosphaeroides
Clostridium_sp_MSTE9
Bacteroides_massiliensis_dnLKV3
Bacteroides
Clostridium_asparagiforme
Clostridiales_bacterium_VE202_15
Mogibacterium_sp_CM50
Mogibacterium_timidum_ATCC_33093
Prevotella_baroniae
Prevotella_baroniae
Solobacterium_moorei
Solobacterium_moorei
Clostridium_butyricum
Clostridiales_bacterium_VE202_13
Faecalibacterium_sp_CAG74
Ruminococcus_sp_CAG403
Clostridium_acetobutylicum
Clostridium_tyrobutyricum
Prevotella_amnii
Prevotella_bivia
Clostridium_butyricum
Anaerotruncus_colihominis
Butyrivibrio_fibrisolvens
Butyrivibrio_fibrisolvens
Clostridium
Clostridium_autoethanogenum_DSM_10061
Prevotella_oralis
Prevotella_oralis
Prevotella_denticola
Prevotella_denticola
Clostridium_kluyveri
Clostridium_kluyveri
Prevotella_dentalis
Hallella_seregens
Corynebacterium_kroppenstedtii
Viridibacillus_arenosi_FSL_R5_213
Staphylococcus_simiae
Staphylococcus
Staphylococcus_pettenkoferi
Staphylococcus
Staphylococcus_aureus_WMCA6006
Staphylococcus_aureus_F50093
Staphylococcus_aureus_W31852
Staphylococcus_aureus_VET1869R
Staphylococcus_aureus_R0615
Staphylococcus_aureus
Staphylococcus_aureus_DAR5856
Staphylococcus_aureus_DAR5852
Staphylococcus_aureus
bacterium_ACCC2
Staphylococcus_aureus_Rd60
Staphylococcus_aureus_subsp_aureus_CIGC341D
Staphylococcus_aureus
Staphylococcus_aureus
Staphylococcus_aureus_subsp_aureus_Mu50
Staphylococcus_aureus_subsp_aureus_21232
Staphylococcus_aureus_W83133
Staphylococcus_aureus_M0314
Staphylococcus_aureus_H70826
Staphylococcus_aureus_T26751
Staphylococcus_aureus_VET0426R
Staphylococcus_aureus_M0162

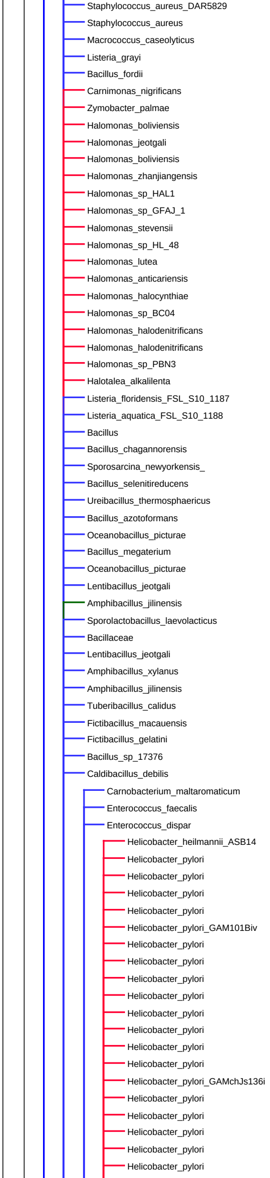
Staphylococcus_aureus_DAR5829
Staphylococcus_aureus
Macrococcus_caseolyticus
Listeria_grayi
Bacillus_fordii
Carnimonas_nigrificans
Zymobacter_palmae
Halomonas_boliviensis
Halomonas_jeotgali
Halomonas_boliviensis
Halomonas_zhanjiangensis
Halomonas_sp_HAL1
Halomonas_sp_GFAJ_1
Halomonas_stevensii
Halomonas_sp_HL_48
Halomonas_lutea
Halomonas_anticariensis
Halomonas_halocynthiae
Halomonas_sp_BC04
Halomonas_halodenitrificans
Halomonas_halodenitrificans
Halomonas_sp_PBN3
Halotalea_alkalilenta
Listeria_floridensis_FSL_S10_1187
Listeria_aquatica_FSL_S10_1188
Bacillus
Bacillus_chagannorensis
Sporosarcina_newyorkensis_
Bacillus_selenitireducens
Ureibacillus_thermosphaericus
Bacillus_azotoformans
Oceanobacillus_picturae
Bacillus_megaterium
Oceanobacillus_picturae
Lentibacillus_jeotgali
Amphibacillus_jilinensis
Sporolactobacillus_laevolacticus
Bacillaceae
Lentibacillus_jeotgali
Amphibacillus_xylanus
Amphibacillus_jilinensis
Tuberibacillus_calidus
Fictibacillus_macauensis
Fictibacillus_gelatini
Bacillus_sp_17376
Caldibacillus_debilis
Carnobacterium_maltaromaticum
Enterococcus_faecalis
Enterococcus_dispar
Helicobacter_heilmannii_ASB14
Helicobacter_pylori
Helicobacter_pylori
Helicobacter_pylori
Helicobacter_pylori
Helicobacter_pylori_GAM101Biv
Helicobacter_pylori
Helicobacter_pylori
Helicobacter_pylori
Helicobacter_pylori
Helicobacter_pylori
Helicobacter_pylori
Helicobacter_pylori
Helicobacter_pylori
Helicobacter_pylori_GAMchJs136i
Helicobacter_pylori
Helicobacter_pylori
Helicobacter_pylori
Helicobacter_pylori
Helicobacter_pylori

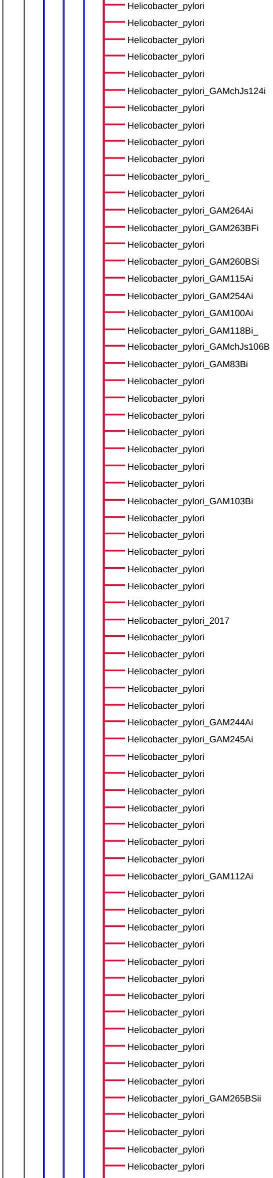

Helicobacter_pylori
Helicobacter_pylori
Helicobacter_pylori
Helicobacter_pylori
Helicobacter_pylori
Helicobacter_pylori_GAMchJs124i
Helicobacter_pylori
Helicobacter_pylori
Helicobacter_pylori
Helicobacter_pylori
Helicobacter_pylori_
Helicobacter_pylori
Helicobacter_pylori_GAM264Ai
Helicobacter_pylori_GAM263BFi
Helicobacter_pylori
Helicobacter_pylori_GAM260BSi
Helicobacter_pylori_GAM115Ai
Helicobacter_pylori_GAM254Ai
Helicobacter_pylori_GAM100Ai
Helicobacter_pylori_GAM118Bi_
Helicobacter_pylori_GAMchJs106B
Helicobacter_pylori_GAM83Bi
Helicobacter_pylori
Helicobacter_pylori
Helicobacter_pylori
Helicobacter_pylori
Helicobacter_pylori
Helicobacter_pylori
Helicobacter_pylori
Helicobacter_pylori_GAM103Bi
Helicobacter_pylori
Helicobacter_pylori
Helicobacter_pylori
Helicobacter_pylori
Helicobacter_pylori
Helicobacter_pylori
Helicobacter_pylori_2017
Helicobacter_pylori
Helicobacter_pylori
Helicobacter_pylori
Helicobacter_pylori
Helicobacter_pylori
Helicobacter_pylori_GAM244Ai
Helicobacter_pylori_GAM245Ai
Helicobacter_pylori
Helicobacter_pylori
Helicobacter_pylori
Helicobacter_pylori
Helicobacter_pylori
Helicobacter_pylori
Helicobacter_pylori
Helicobacter_pylori_GAM112Ai
Helicobacter_pylori
Helicobacter_pylori
Helicobacter_pylori
Helicobacter_pylori
Helicobacter_pylori
Helicobacter_pylori
Helicobacter_pylori
Helicobacter_pylori
Helicobacter_pylori
Helicobacter_pylori
Helicobacter_pylori
Helicobacter_pylori
Helicobacter_pylori_GAM265BSii
Helicobacter_pylori
Helicobacter_pylori
Helicobacter_pylori
Helicobacter_pylori

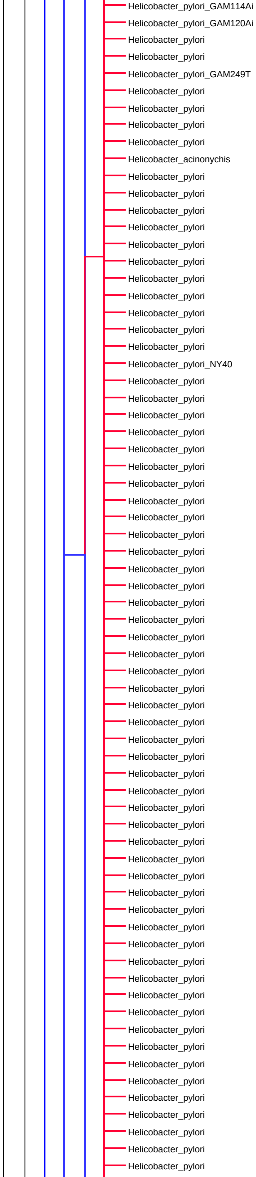

Helicobacter_pylori_GAM114Ai
Helicobacter_pylori_GAM120Ai
Helicobacter_pylori
Helicobacter_pylori
Helicobacter_pylori_GAM249T
Helicobacter_pylori
Helicobacter_pylori
Helicobacter_pylori
Helicobacter_pylori
Helicobacter_acinonychis
Helicobacter_pylori
Helicobacter_pylori
Helicobacter_pylori
Helicobacter_pylori
Helicobacter_pylori
Helicobacter_pylori
Helicobacter_pylori
Helicobacter_pylori
Helicobacter_pylori
Helicobacter_pylori
Helicobacter_pylori
Helicobacter_pylori_NY40
Helicobacter_pylori
Helicobacter_pylori
Helicobacter_pylori
Helicobacter_pylori
Helicobacter_pylori
Helicobacter_pylori
Helicobacter_pylori
Helicobacter_pylori
Helicobacter_pylori
Helicobacter_pylori
Helicobacter_pylori
Helicobacter_pylori
Helicobacter_pylori
Helicobacter_pylori
Helicobacter_pylori
Helicobacter_pylori
Helicobacter_pylori
Helicobacter_pylori
Helicobacter_pylori
Helicobacter_pylori
Helicobacter_pylori
Helicobacter_pylori
Helicobacter_pylori
Helicobacter_pylori
Helicobacter_pylori
Helicobacter_pylori
Helicobacter_pylori
Helicobacter_pylori
Helicobacter_pylori
Helicobacter_pylori
Helicobacter_pylori
Helicobacter_pylori
Helicobacter_pylori
Helicobacter_pylori
Helicobacter_pylori
Helicobacter_pylori
Helicobacter_pylori
Helicobacter_pylori
Helicobacter_pylori
Helicobacter_pylori
Helicobacter_pylori
Helicobacter_pylori
Helicobacter_pylori
Helicobacter_pylori
Helicobacter_pylori
Helicobacter_pylori
Helicobacter_pylori

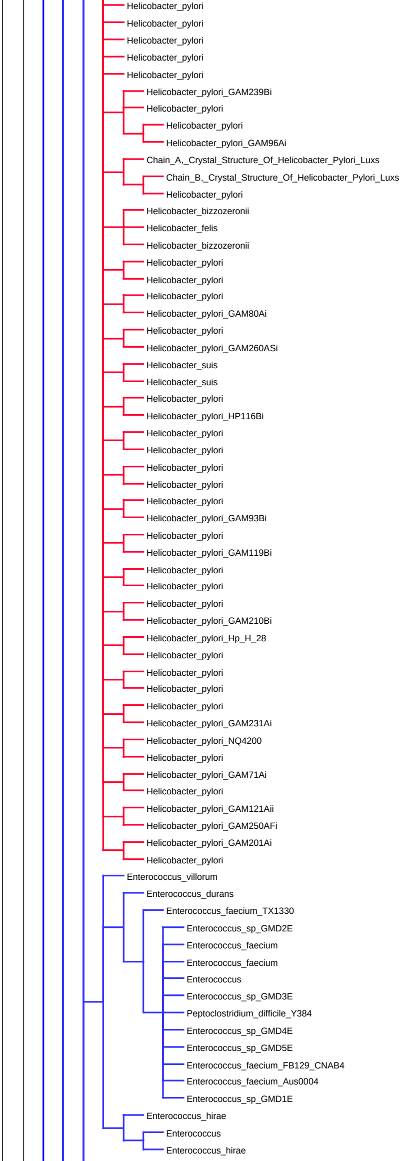
Helicobacter_pylori
Helicobacter_pylori
Helicobacter_pylori
Helicobacter_pylori
Helicobacter_pylori
Helicobacter_pylori_GAM239Bi
Helicobacter_pylori
Helicobacter_pylori
Helicobacter_pylori_GAM96Ai
Chain_A,_Crystal_Structure_Of_Helicobacter_Pylori_Luxs
Chain_B,_Crystal_Structure_Of_Helicobacter_Pylori_Luxs
Helicobacter_pylori
Helicobacter_bizzozeronii
Helicobacter_felis
Helicobacter_bizzozeronii
Helicobacter_pylori
Helicobacter_pylori
Helicobacter_pylori
Helicobacter_pylori_GAM80Ai
Helicobacter_pylori
Helicobacter_pylori_GAM260ASi
Helicobacter_suis
Helicobacter_suis
Helicobacter_pylori
Helicobacter_pylori_HP116Bi
Helicobacter_pylori
Helicobacter_pylori
Helicobacter_pylori
Helicobacter_pylori
Helicobacter_pylori
Helicobacter_pylori_GAM93Bi
Helicobacter_pylori
Helicobacter_pylori_GAM119Bi
Helicobacter_pylori
Helicobacter_pylori
Helicobacter_pylori
Helicobacter_pylori_GAM210Bi
Helicobacter_pylori_Hp_H_28
Helicobacter_pylori
Helicobacter_pylori
Helicobacter_pylori
Helicobacter_pylori
Helicobacter_pylori_GAM231Ai
Helicobacter_pylori_NQ4200
Helicobacter_pylori
Helicobacter_pylori_GAM71Ai
Helicobacter_pylori
Helicobacter_pylori_GAM121Aii
Helicobacter_pylori_GAM250AFi
Helicobacter_pylori_GAM201Ai
Helicobacter_pylori
Enterococcus_villorum
Enterococcus_durans
Enterococcus_faecium_TX1330
Enterococcus_sp_GMD2E
Enterococcus_faecium
Enterococcus_faecium
Enterococcus
Enterococcus_sp_GMD3E
Peptoclostridium_difficile_Y384
Enterococcus_sp_GMD4E
Enterococcus_sp_GMD5E
Enterococcus_faecium_FB129_CNAB4
Enterococcus_faecium_Aus0004
Enterococcus_sp_GMD1E
Enterococcus_hirae
Enterococcus
Enterococcus_hirae

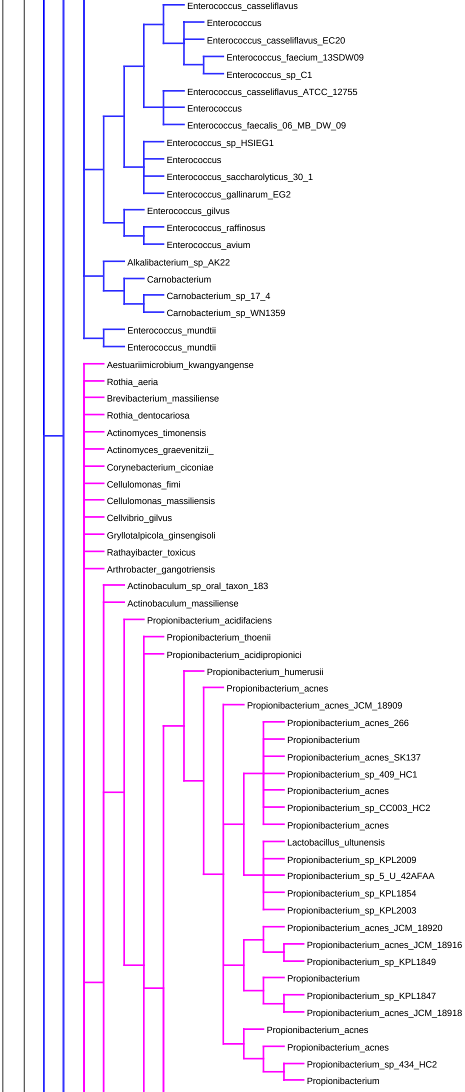
Enterococcus_casseliflavus
Enterococcus
Enterococcus_casseliflavus_EC20
Enterococcus_faecium_13SDW09
Enterococcus_sp_C1
Enterococcus_casseliflavus_ATCC_12755
Enterococcus
Enterococcus_faecalis_06_MB_DW_09
Enterococcus_sp_HSIEG1
Enterococcus
Enterococcus_saccharolyticus_30_1
Enterococcus_gallinarum_EG2
Enterococcus_gilvus
Enterococcus_raffinosus
Enterococcus_avium
Alkalibacterium_sp_AK22
Carnobacterium
Carnobacterium_sp_17_4
Carnobacterium_sp_WN1359
Enterococcus_mundtii
Enterococcus_mundtii
Aestuariimicrobium_kwangyangense
Rothia_aeria
Brevibacterium_massiliense
Rothia_dentocariosa
Actinomyces_timonensis
Actinomyces_graevenitzii_
Corynebacterium_ciconiae
Cellulomonas_fimi
Cellulomonas_massiliensis
Cellvibrio_gilvus
Gryllotalpicola_ginsengisoli
Rathayibacter_toxicus
Arthrobacter_gangotriensis
Actinobaculum_sp_oral_taxon_183
Actinobaculum_massiliense
Propionibacterium_acidifaciens
Propionibacterium_thoenii
Propionibacterium_acidipropionici
Propionibacterium_humerusii
Propionibacterium_acnes
Propionibacterium_acnes_JCM_18909
Propionibacterium_acnes_266
Propionibacterium
Propionibacterium_acnes_SK137
Propionibacterium_sp_409_HC1
Propionibacterium_acnes
Propionibacterium_sp_CC003_HC2
Propionibacterium_acnes
Lactobacillus_ultunensis
Propionibacterium_sp_KPL2009
Propionibacterium_sp_5_U_42AFAA
Propionibacterium_sp_KPL1854
Propionibacterium_sp_KPL2003
Propionibacterium_acnes_JCM_18920
Propionibacterium_acnes_JCM_18916
Propionibacterium_sp_KPL1849
Propionibacterium
Propionibacterium_sp_KPL1847
Propionibacterium_acnes_JCM_18918
Propionibacterium_acnes
Propionibacterium_acnes
Propionibacterium_sp_434_HC2
Propionibacterium

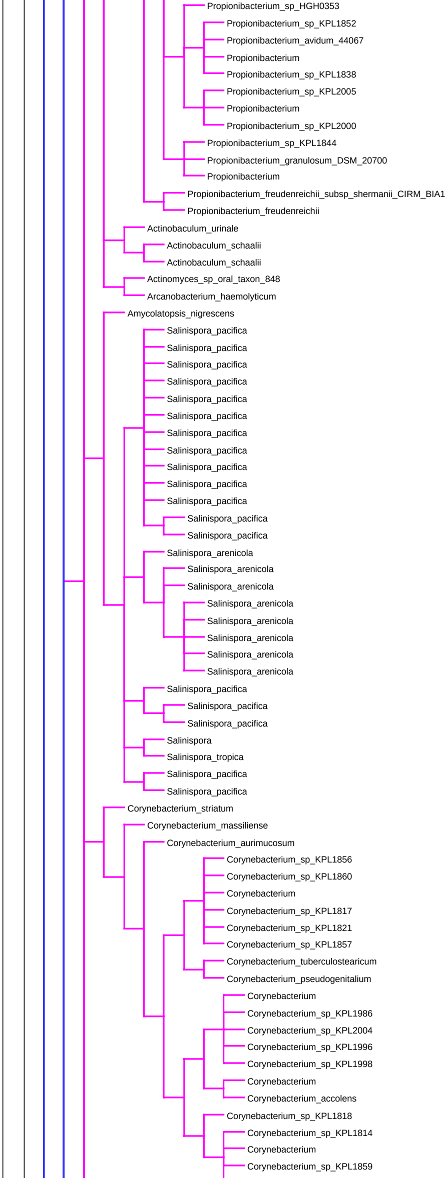
Propionibacterium_sp_HGH0353
Propionibacterium_sp_KPL1852
Propionibacterium_avidum_44067
Propionibacterium
Propionibacterium_sp_KPL1838
Propionibacterium_sp_KPL2005
Propionibacterium
Propionibacterium_sp_KPL2000
Propionibacterium_sp_KPL1844
Propionibacterium_granulosum_DSM_20700
Propionibacterium
Propionibacterium_freudenreichii_subsp_shermanii_CIRM_BIA1
Propionibacterium_freudenreichii
Actinobaculum_urinale
Actinobaculum_schaalii
Actinobaculum_schaalii
Actinomyces_sp_oral_taxon_848
Arcanobacterium_haemolyticum
Amycolatopsis_nigrescens
Salinispora_pacifica
Salinispora_pacifica
Salinispora_pacifica
Salinispora_pacifica
Salinispora_pacifica
Salinispora_pacifica
Salinispora_pacifica
Salinispora_pacifica
Salinispora_pacifica
Salinispora_pacifica
Salinispora_pacifica
Salinispora_pacifica
Salinispora_pacifica
Salinispora_arenicola
Salinispora_arenicola
Salinispora_arenicola
Salinispora_arenicola
Salinispora_arenicola
Salinispora_arenicola
Salinispora_arenicola
Salinispora_arenicola
Salinispora_pacifica
Salinispora_pacifica
Salinispora_pacifica
Salinispora
Salinispora_tropica
Salinispora_pacifica
Salinispora_pacifica
Corynebacterium_striatum
Corynebacterium_massiliense
Corynebacterium_aurimucosum
Corynebacterium_sp_KPL1856
Corynebacterium_sp_KPL1860
Corynebacterium
Corynebacterium_sp_KPL1817
Corynebacterium_sp_KPL1821
Corynebacterium_sp_KPL1857
Corynebacterium_tuberculostearicum
Corynebacterium_pseudogenitalium
Corynebacterium
Corynebacterium_sp_KPL1986
Corynebacterium_sp_KPL2004
Corynebacterium_sp_KPL1996
Corynebacterium_sp_KPL1998
Corynebacterium
Corynebacterium_accolens
Corynebacterium_sp_KPL1818
Corynebacterium_sp_KPL1814
Corynebacterium
Corynebacterium_sp_KPL1859

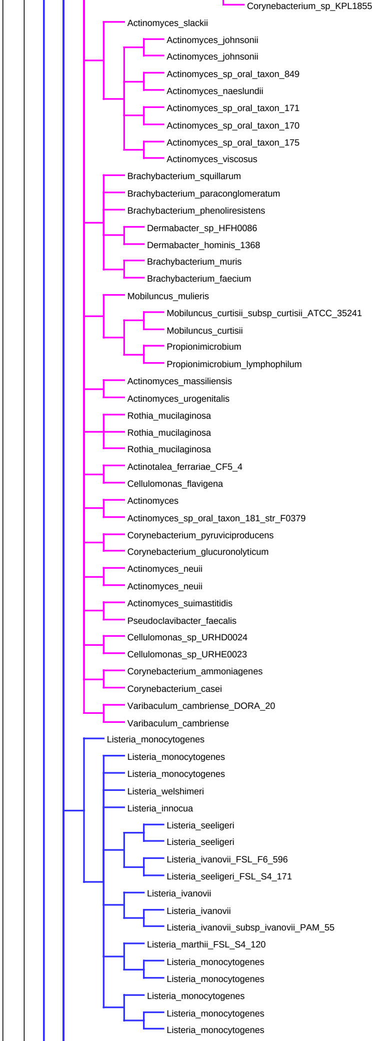
Corynebacterium_sp_KPL1855
Actinomyces_slackii
Actinomyces_johnsonii
Actinomyces_johnsonii
Actinomyces_sp_oral_taxon_849
Actinomyces_naeslundii
Actinomyces_sp_oral_taxon_171
Actinomyces_sp_oral_taxon_170
Actinomyces_sp_oral_taxon_175
Actinomyces_viscosus
Brachybacterium_squillarum
Brachybacterium_paraconglomeratum
Brachybacterium_phenoliresistens
Dermabacter_sp_HFH0086
Dermabacter_hominis_1368
Brachybacterium_muris
Brachybacterium_faecium
Mobiluncus_mulieris
Mobiluncus_curtisii_subsp_curtisii_ATCC_35241
Mobiluncus_curtisii
Propionimicrobium
Propionimicrobium_lymphophilum
Actinomyces_massiliensis
Actinomyces_urogenitalis
Rothia_mucilaginosa
Rothia_mucilaginosa
Rothia_mucilaginosa
Actinotalea_ferrariae_CF5_4
Cellulomonas_flavigena
Actinomyces
Actinomyces_sp_oral_taxon_181_str_F0379
Corynebacterium_pyruviciproducens
Corynebacterium_glucuronolyticum
Actinomyces_neuii
Actinomyces_neuii
Actinomyces_suimastitidis
Pseudoclavibacter_faecalis
Cellulomonas_sp_URHD0024
Cellulomonas_sp_URHE0023
Corynebacterium_ammoniagenes
Corynebacterium_casei
Varibaculum_cambriense_DORA_20
Varibaculum_cambriense
Listeria_monocytogenes
Listeria_monocytogenes
Listeria_monocytogenes
Listeria_welshimeri
Listeria_innocua
Listeria_seeligeri
Listeria_seeligeri
Listeria_ivanovii_FSL_F6_596
Listeria_seeligeri_FSL_S4_171
Listeria_ivanovii
Listeria_ivanovii
Listeria_ivanovii_subsp_ivanovii_PAM_55
Listeria_marthii_FSL_S4_120
Listeria_monocytogenes
Listeria_monocytogenes
Listeria_monocytogenes
Listeria_monocytogenes
Listeria_monocytogenes

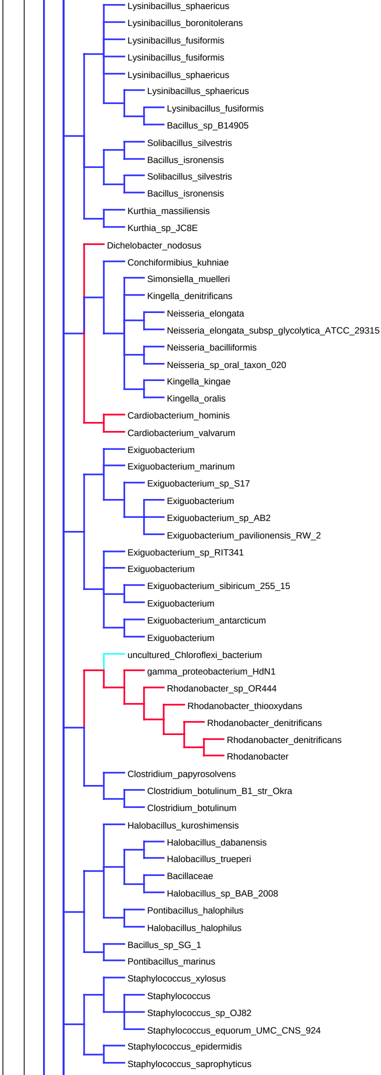
Lysinibacillus_sphaericus
Lysinibacillus_boronitolerans
Lysinibacillus_fusiformis
Lysinibacillus_fusiformis
Lysinibacillus_sphaericus
Lysinibacillus_sphaericus
Lysinibacillus_fusiformis
Bacillus_sp_B14905
Solibacillus_silvestris
Bacillus_isronensis
Solibacillus_silvestris
Bacillus_isronensis
Kurthia_massiliensis
Kurthia_sp_JC8E
Dichelobacter_nodosus
Conchiformibius_kuhniae
Simonsiella_muelleri
Kingella_denitrificans
Neisseria_elongata
Neisseria_elongata_subsp_glycolytica_ATCC_29315
Neisseria_bacilliformis
Neisseria_sp_oral_taxon_020
Kingella_kingae
Kingella_oralis
Cardiobacterium_hominis
Cardiobacterium_valvarum
Exiguobacterium
Exiguobacterium_marinum
Exiguobacterium_sp_S17
Exiguobacterium
Exiguobacterium_sp_AB2
Exiguobacterium_pavilionensis_RW_2
Exiguobacterium_sp_RIT341
Exiguobacterium
Exiguobacterium_sibiricum_255_15
Exiguobacterium
Exiguobacterium_antarcticum
Exiguobacterium
uncultured_Chloroflexi_bacterium
gamma_proteobacterium_HdN1
Rhodanobacter_sp_OR444
Rhodanobacter_thiooxydans
Rhodanobacter_denitrificans
Rhodanobacter_denitrificans
Rhodanobacter
Clostridium_papyrosolvens
Clostridium_botulinum_B1_str_Okra
Clostridium_botulinum
Halobacillus_kuroshimensis
Halobacillus_dabanensis
Halobacillus_trueperi
Bacillaceae
Halobacillus_sp_BAB_2008
Pontibacillus_halophilus
Halobacillus_halophilus
Bacillus_sp_SG_1
Pontibacillus_marinus
Staphylococcus_xylosus
Staphylococcus
Staphylococcus_sp_OJ82
Staphylococcus_equorum_UMC_CNS_924
Staphylococcus_epidermidis
Staphylococcus_saprophyticus

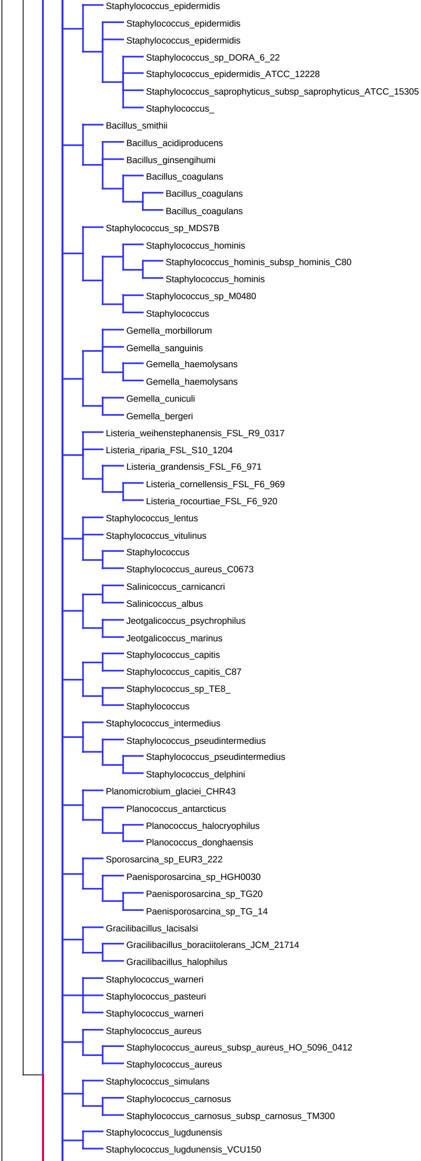
Staphylococcus_epidermidis
Staphylococcus_epidermidis
Staphylococcus_epidermidis
Staphylococcus_sp_DORA_6_22
Staphylococcus_epidermidis_ATCC_12228
Staphylococcus_saprophyticus_subsp_saprophyticus_ATCC_15305
Staphylococcus_
Bacillus_smithii
Bacillus_acidiproducens
Bacillus_ginsengihumi
Bacillus_coagulans
Bacillus_coagulans
Bacillus_coagulans
Staphylococcus_sp_MDS7B
Staphylococcus_hominis
Staphylococcus_hominis_subsp_hominis_C80
Staphylococcus_hominis
Staphylococcus_sp_M0480
Staphylococcus
Gemella_morbillorum
Gemella_sanguinis
Gemella_haemolysans
Gemella_haemolysans
Gemella_cuniculi
Gemella_bergeri
Listeria_weihenstephanensis_FSL_R9_0317
Listeria_riparia_FSL_S10_1204
Listeria_grandensis_FSL_F6_971
Listeria_cornellensis_FSL_F6_969
Listeria_rocourtiae_FSL_F6_920
Staphylococcus_lentus
Staphylococcus_vitulinus
Staphylococcus
Staphylococcus_aureus_C0673
Salinicoccus_carnicancri
Salinicoccus_albus
Jeotgalicoccus_psychrophilus
Jeotgalicoccus_marinus
Staphylococcus_capitis
Staphylococcus_capitis_C87
Staphylococcus_sp_TE8_
Staphylococcus
Staphylococcus_intermedius
Staphylococcus_pseudintermedius
Staphylococcus_pseudintermedius
Staphylococcus_delphini
Planomicrobium_glaciei_CHR43
Planococcus_antarcticus
Planococcus_halocryophilus
Planococcus_donghaensis
Sporosarcina_sp_EUR3_222
Paenisporosarcina_sp_HGH0030
Paenisporosarcina_sp_TG20
Paenisporosarcina_sp_TG_14
Gracilibacillus_lacisalsi
Gracilibacillus_boraciitolerans_JCM_21714
Gracilibacillus_halophilus
Staphylococcus_warneri
Staphylococcus_pasteuri
Staphylococcus_warneri
Staphylococcus_aureus
Staphylococcus_aureus_subsp_aureus_HO_5096_0412
Staphylococcus_aureus
Staphylococcus_simulans
Staphylococcus_carnosus
Staphylococcus_carnosus_subsp_carnosus_TM300
Staphylococcus_lugdunensis
Staphylococcus_lugdunensis_VCU150

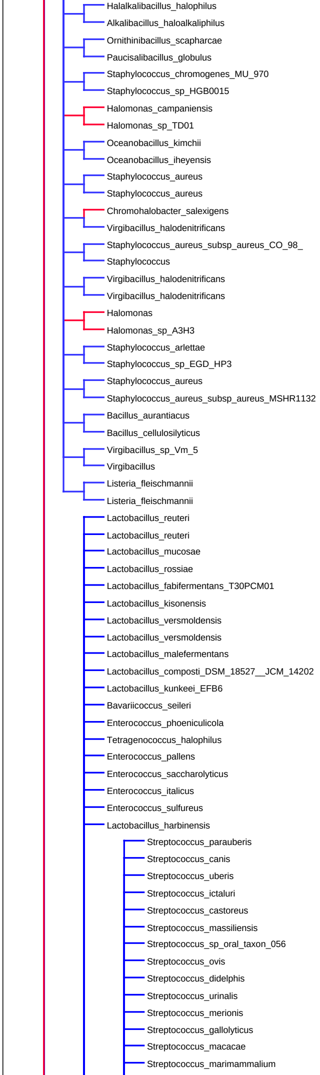
Halalkalibacillus_halophilus
Alkalibacillus_haloalkaliphilus
Ornithinibacillus_scapharcae
Paucisalibacillus_globulus
Staphylococcus_chromogenes_MU_970
Staphylococcus_sp_HGB0015
Halomonas_campaniensis
Halomonas_sp_TD01
Oceanobacillus_kimchii
Oceanobacillus_iheyensis
Staphylococcus_aureus
Staphylococcus_aureus
Chromohalobacter_salexigens
Virgibacillus_halodenitrificans
Staphylococcus_aureus_subsp_aureus_CO_98_
Staphylococcus
Virgibacillus_halodenitrificans
Virgibacillus_halodenitrificans
Halomonas
Halomonas_sp_A3H3
Staphylococcus_arlettae
Staphylococcus_sp_EGD_HP3
Staphylococcus_aureus
Staphylococcus_aureus_subsp_aureus_MSHR1132
Bacillus_aurantiacus
Bacillus_cellulosilyticus
Virgibacillus_sp_Vm_5
Virgibacillus
Listeria_fleischmannii
Listeria_fleischmannii
Lactobacillus_reuteri
Lactobacillus_reuteri
Lactobacillus_mucosae
Lactobacillus_rossiae
Lactobacillus_fabifermentans_T30PCM01
Lactobacillus_kisonensis
Lactobacillus_versmoldensis
Lactobacillus_versmoldensis
Lactobacillus_malefermentans
Lactobacillus_composti_DSM_18527__JCM_14202
Lactobacillus_kunkeei_EFB6
Bavariicoccus_seileri
Enterococcus_phoeniculicola
Tetragenococcus_halophilus
Enterococcus_pallens
Enterococcus_saccharolyticus
Enterococcus_italicus
Enterococcus_sulfureus
Lactobacillus_harbinensis
Streptococcus_parauberis
Streptococcus_canis
Streptococcus_uberis
Streptococcus_ictaluri
Streptococcus_castoreus
Streptococcus_massiliensis
Streptococcus_sp_oral_taxon_056
Streptococcus_ovis
Streptococcus_didelphis
Streptococcus_urinalis
Streptococcus_merionis
Streptococcus_gallolyticus
Streptococcus_macacae
Streptococcus_marimammalium

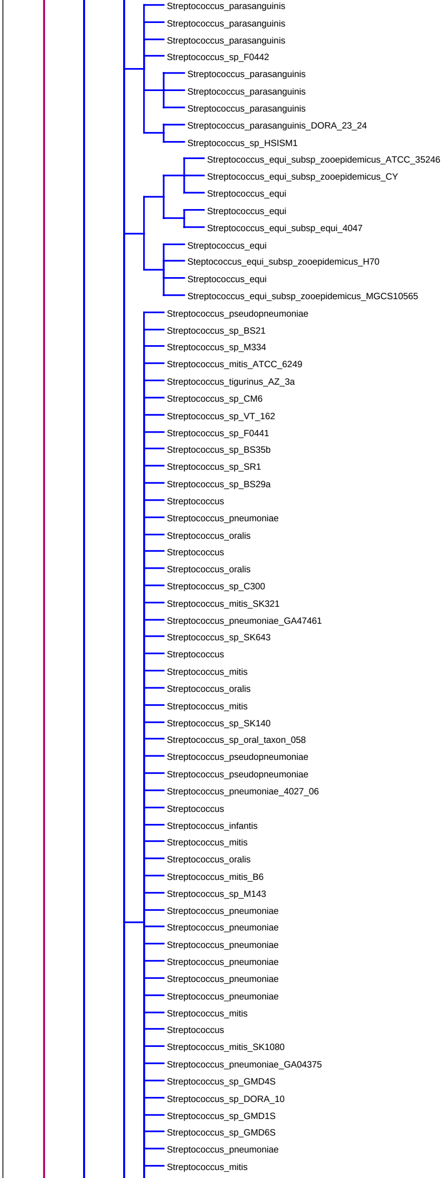
Streptococcus_parasanguinis
Streptococcus_parasanguinis
Streptococcus_parasanguinis
Streptococcus_sp_F0442
Streptococcus_parasanguinis
Streptococcus_parasanguinis
Streptococcus_parasanguinis
Streptococcus_parasanguinis_DORA_23_24
Streptococcus_sp_HSISM1
Streptococcus_equi_subsp_zooepidemicus_ATCC_35246
Streptococcus_equi_subsp_zooepidemicus_CY
Streptococcus_equi
Streptococcus_equi
Streptococcus_equi_subsp_equi_4047
Streptococcus_equi
Steptococcus_equi_subsp_zooepidemicus_H70
Streptococcus_equi
Streptococcus_equi_subsp_zooepidemicus_MGCS10565
Streptococcus_pseudopneumoniae
Streptococcus_sp_BS21
Streptococcus_sp_M334
Streptococcus_mitis_ATCC_6249
Streptococcus_tigurinus_AZ_3a
Streptococcus_sp_CM6
Streptococcus_sp_VT_162
Streptococcus_sp_F0441
Streptococcus_sp_BS35b
Streptococcus_sp_SR1
Streptococcus_sp_BS29a
Streptococcus
Streptococcus_pneumoniae
Streptococcus_oralis
Streptococcus
Streptococcus_oralis
Streptococcus_sp_C300
Streptococcus_mitis_SK321
Streptococcus_pneumoniae_GA47461
Streptococcus_sp_SK643
Streptococcus
Streptococcus_mitis
Streptococcus_oralis
Streptococcus_mitis
Streptococcus_sp_SK140
Streptococcus_sp_oral_taxon_058
Streptococcus_pseudopneumoniae
Streptococcus_pseudopneumoniae
Streptococcus_pneumoniae_4027_06
Streptococcus
Streptococcus_infantis
Streptococcus_mitis
Streptococcus_oralis
Streptococcus_mitis_B6
Streptococcus_sp_M143
Streptococcus_pneumoniae
Streptococcus_pneumoniae
Streptococcus_pneumoniae
Streptococcus_pneumoniae
Streptococcus_pneumoniae
Streptococcus_pneumoniae
Streptococcus_mitis
Streptococcus
Streptococcus_mitis_SK1080
Streptococcus_pneumoniae_GA04375
Streptococcus_sp_GMD4S
Streptococcus_sp_DORA_10
Streptococcus_sp_GMD1S
Streptococcus_sp_GMD6S
Streptococcus_pneumoniae
Streptococcus_mitis

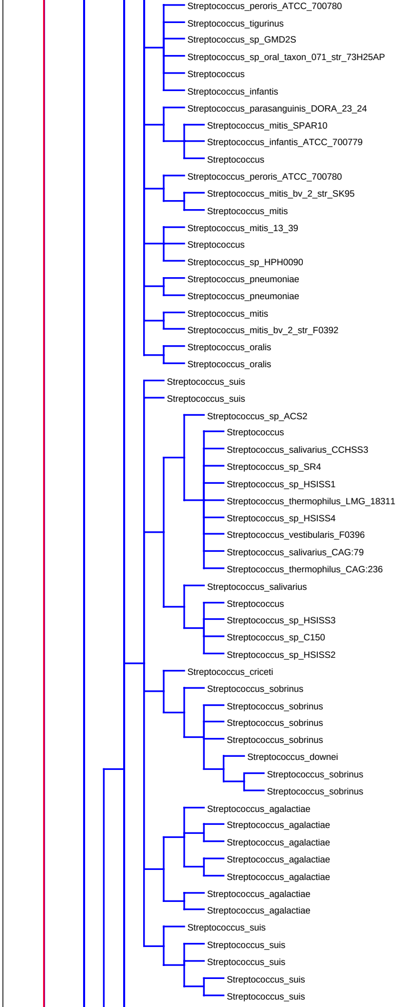
Streptococcus_peroris_ATCC_700780
Streptococcus_tigurinus
Streptococcus_sp_GMD2S
Streptococcus_sp_oral_taxon_071_str_73H25AP
Streptococcus
Streptococcus_infantis
Streptococcus_parasanguinis_DORA_23_24
Streptococcus_mitis_SPAR10
Streptococcus_infantis_ATCC_700779
Streptococcus
Streptococcus_peroris_ATCC_700780
Streptococcus_mitis_bv_2_str_SK95
Streptococcus_mitis
Streptococcus_mitis_13_39
Streptococcus
Streptococcus_sp_HPH0090
Streptococcus_pneumoniae
Streptococcus_pneumoniae
Streptococcus_mitis
Streptococcus_mitis_bv_2_str_F0392
Streptococcus_oralis
Streptococcus_oralis
Streptococcus_suis
Streptococcus_suis
Streptococcus_sp_ACS2
Streptococcus
Streptococcus_salivarius_CCHSS3
Streptococcus_sp_SR4
Streptococcus_sp_HSISS1
Streptococcus_thermophilus_LMG_18311
Streptococcus_sp_HSISS4
Streptococcus_vestibularis_F0396
Streptococcus_salivarius_CAG:79
Streptococcus_thermophilus_CAG:236
Streptococcus_salivarius
Streptococcus
Streptococcus_sp_HSISS3
Streptococcus_sp_C150
Streptococcus_sp_HSISS2
Streptococcus_criceti
Streptococcus_sobrinus
Streptococcus_sobrinus
Streptococcus_sobrinus
Streptococcus_sobrinus
Streptococcus_downei
Streptococcus_sobrinus
Streptococcus_sobrinus
Streptococcus_agalactiae
Streptococcus_agalactiae
Streptococcus_agalactiae
Streptococcus_agalactiae
Streptococcus_agalactiae
Streptococcus_agalactiae
Streptococcus_agalactiae
Streptococcus_suis
Streptococcus_suis
Streptococcus_suis
Streptococcus_suis
Streptococcus_suis

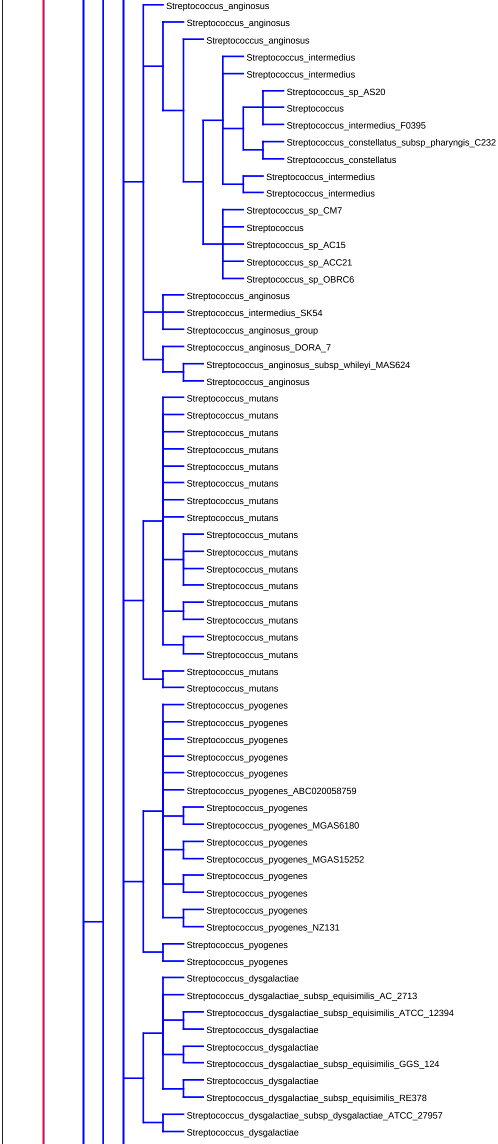
Streptococcus_anginosus
Streptococcus_anginosus
Streptococcus_anginosus
Streptococcus_intermedius
Streptococcus_intermedius
Streptococcus_sp_AS20
Streptococcus
Streptococcus_intermedius_F0395
Streptococcus_constellatus_subsp_pharyngis_C232
Streptococcus_constellatus
Streptococcus_intermedius
Streptococcus_intermedius
Streptococcus_sp_CM7
Streptococcus
Streptococcus_sp_AC15
Streptococcus_sp_ACC21
Streptococcus_sp_OBRC6
Streptococcus_anginosus
Streptococcus_intermedius_SK54
Streptococcus_anginosus_group
Streptococcus_anginosus_DORA_7
Streptococcus_anginosus_subsp_whileyi_MAS624
Streptococcus_anginosus
Streptococcus_mutans
Streptococcus_mutans
Streptococcus_mutans
Streptococcus_mutans
Streptococcus_mutans
Streptococcus_mutans
Streptococcus_mutans
Streptococcus_mutans
Streptococcus_mutans
Streptococcus_mutans
Streptococcus_mutans
Streptococcus_mutans
Streptococcus_mutans
Streptococcus_mutans
Streptococcus_mutans
Streptococcus_mutans
Streptococcus_mutans
Streptococcus_mutans
Streptococcus_pyogenes
Streptococcus_pyogenes
Streptococcus_pyogenes
Streptococcus_pyogenes
Streptococcus_pyogenes
Streptococcus_pyogenes_ABC020058759
Streptococcus_pyogenes
Streptococcus_pyogenes_MGAS6180
Streptococcus_pyogenes
Streptococcus_pyogenes_MGAS15252
Streptococcus_pyogenes
Streptococcus_pyogenes
Streptococcus_pyogenes
Streptococcus_pyogenes_NZ131
Streptococcus_pyogenes
Streptococcus_pyogenes
Streptococcus_dysgalactiae
Streptococcus_dysgalactiae_subsp_equisimilis_AC_2713
Streptococcus_dysgalactiae_subsp_equisimilis_ATCC_12394
Streptococcus_dysgalactiae
Streptococcus_dysgalactiae
Streptococcus_dysgalactiae_subsp_equisimilis_GGS_124
Streptococcus_dysgalactiae
Streptococcus_dysgalactiae_subsp_equisimilis_RE378
Streptococcus_dysgalactiae_subsp_dysgalactiae_ATCC_27957
Streptococcus_dysgalactiae

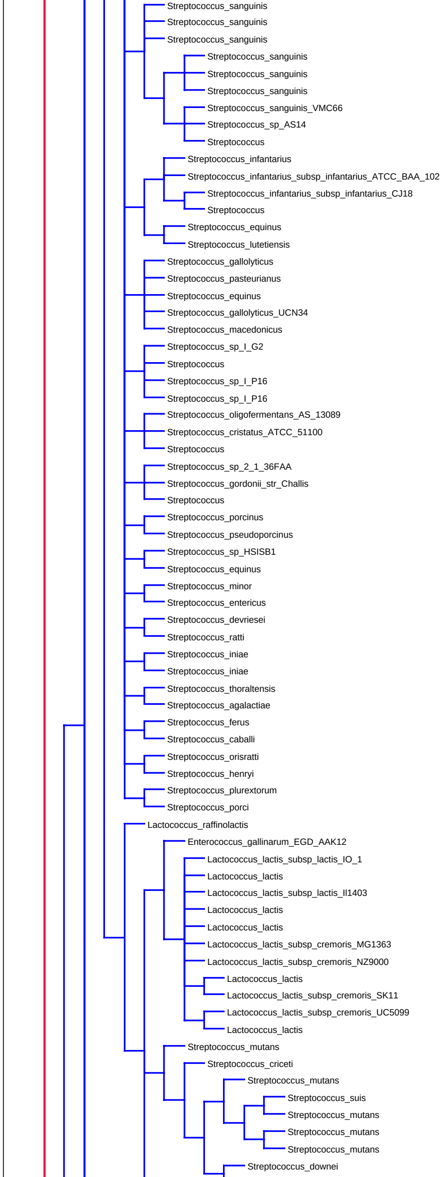
Streptococcus_sanguinis
Streptococcus_sanguinis
Streptococcus_sanguinis
Streptococcus_sanguinis
Streptococcus_sanguinis
Streptococcus_sanguinis
Streptococcus_sanguinis_VMC66
Streptococcus_sp_AS14
Streptococcus
Streptococcus_infantarius
Streptococcus_infantarius_subsp_infantarius_ATCC_BAA_102
Streptococcus_infantarius_subsp_infantarius_CJ18
Streptococcus
Streptococcus_equinus
Streptococcus_lutetiensis
Streptococcus_gallolyticus
Streptococcus_pasteurianus
Streptococcus_equinus
Streptococcus_gallolyticus_UCN34
Streptococcus_macedonicus
Streptococcus_sp_I_G2
Streptococcus
Streptococcus_sp_I_P16
Streptococcus_sp_I_P16
Streptococcus_oligofermentans_AS_13089
Streptococcus_cristatus_ATCC_51100
Streptococcus
Streptococcus_sp_2_1_36FAA
Streptococcus_gordonii_str_Challis
Streptococcus
Streptococcus_porcinus
Streptococcus_pseudoporcinus
Streptococcus_sp_HSISB1
Streptococcus_equinus
Streptococcus_minor
Streptococcus_entericus
Streptococcus_devriesei
Streptococcus_ratti
Streptococcus_iniae
Streptococcus_iniae
Streptococcus_thoraltensis
Streptococcus_agalactiae
Streptococcus_ferus
Streptococcus_caballi
Streptococcus_orisratti
Streptococcus_henryi
Streptococcus_plurextorum
Streptococcus_porci
Lactococcus_raffinolactis
Enterococcus_gallinarum_EGD_AAK12
Lactococcus_lactis_subsp_lactis_IO_1
Lactococcus_lactis
Lactococcus_lactis_subsp_lactis_Il1403
Lactococcus_lactis
Lactococcus_lactis
Lactococcus_lactis_subsp_cremoris_MG1363
Lactococcus_lactis_subsp_cremoris_NZ9000
Lactococcus_lactis
Lactococcus_lactis_subsp_cremoris_SK11
Lactococcus_lactis_subsp_cremoris_UC5099
Lactococcus_lactis
Streptococcus_mutans
Streptococcus_criceti
Streptococcus_mutans
Streptococcus_suis
Streptococcus_mutans
Streptococcus_mutans
Streptococcus_mutans
Streptococcus_downei

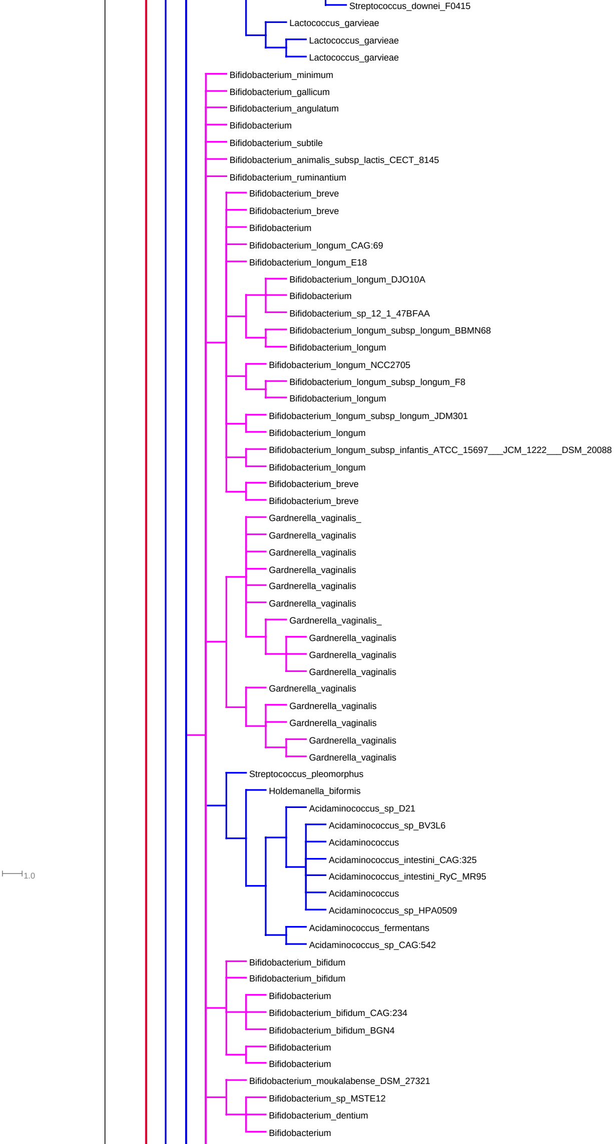
Streptococcus_downei_F0415
Lactococcus_garvieae
Lactococcus_garvieae
Lactococcus_garvieae
Bifidobacterium_minimum
Bifidobacterium_gallicum
Bifidobacterium_angulatum
Bifidobacterium
Bifidobacterium_subtile
Bifidobacterium_animalis_subsp_lactis_CECT_8145
Bifidobacterium_ruminantium
Bifidobacterium_breve
Bifidobacterium_breve
Bifidobacterium
Bifidobacterium_longum_CAG:69
Bifidobacterium_longum_E18
Bifidobacterium_longum_DJO10A
Bifidobacterium
Bifidobacterium_sp_12_1_47BFAA
Bifidobacterium_longum_subsp_longum_BBMN68
Bifidobacterium_longum
Bifidobacterium_longum_NCC2705
Bifidobacterium_longum_subsp_longum_F8
Bifidobacterium_longum
Bifidobacterium_longum_subsp_longum_JDM301
Bifidobacterium_longum
Bifidobacterium_longum_subsp_infantis_ATCC_15697___JCM_1222___DSM_20088
Bifidobacterium_longum
Bifidobacterium_breve
Bifidobacterium_breve
Gardnerella_vaginalis_
Gardnerella_vaginalis
Gardnerella_vaginalis
Gardnerella_vaginalis
Gardnerella_vaginalis
Gardnerella_vaginalis
Gardnerella_vaginalis_
Gardnerella_vaginalis
Gardnerella_vaginalis
Gardnerella_vaginalis
Gardnerella_vaginalis
Gardnerella_vaginalis
Gardnerella_vaginalis
Gardnerella_vaginalis
Gardnerella_vaginalis
Streptococcus_pleomorphus
Holdemanella_biformis
Acidaminococcus_sp_D21
Acidaminococcus_sp_BV3L6
Acidaminococcus
Acidaminococcus_intestini_CAG:325
Acidaminococcus_intestini_RyC_MR95
Acidaminococcus
Acidaminococcus_sp_HPA0509
Acidaminococcus_fermentans
Acidaminococcus_sp_CAG:542
Bifidobacterium_bifidum
Bifidobacterium_bifidum
Bifidobacterium
Bifidobacterium_bifidum_CAG:234
Bifidobacterium_bifidum_BGN4
Bifidobacterium
Bifidobacterium
Bifidobacterium_moukalabense_DSM_27321
Bifidobacterium_sp_MSTE12
Bifidobacterium_dentium
Bifidobacterium
1.0

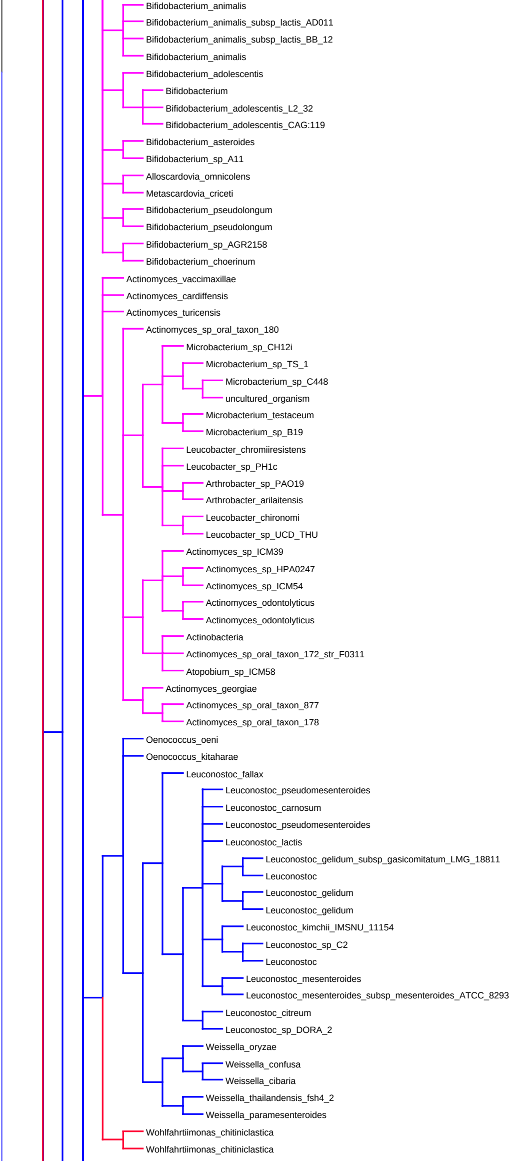
Bifidobacterium_animalis
Bifidobacterium_animalis_subsp_lactis_AD011
Bifidobacterium_animalis_subsp_lactis_BB_12
Bifidobacterium_animalis
Bifidobacterium_adolescentis
Bifidobacterium
Bifidobacterium_adolescentis_L2_32
Bifidobacterium_adolescentis_CAG:119
Bifidobacterium_asteroides
Bifidobacterium_sp_A11
Alloscardovia_omnicolens
Metascardovia_criceti
Bifidobacterium_pseudolongum
Bifidobacterium_pseudolongum
Bifidobacterium_sp_AGR2158
Bifidobacterium_choerinum
Actinomyces_vaccimaxillae
Actinomyces_cardiffensis
Actinomyces_turicensis
Actinomyces_sp_oral_taxon_180
Microbacterium_sp_CH12i
Microbacterium_sp_TS_1
Microbacterium_sp_C448
uncultured_organism
Microbacterium_testaceum
Microbacterium_sp_B19
Leucobacter_chromiiresistens
Leucobacter_sp_PH1c
Arthrobacter_sp_PAO19
Arthrobacter_arilaitensis
Leucobacter_chironomi
Leucobacter_sp_UCD_THU
Actinomyces_sp_ICM39
Actinomyces_sp_HPA0247
Actinomyces_sp_ICM54
Actinomyces_odontolyticus
Actinomyces_odontolyticus
Actinobacteria
Actinomyces_sp_oral_taxon_172_str_F0311
Atopobium_sp_ICM58
Actinomyces_georgiae
Actinomyces_sp_oral_taxon_877
Actinomyces_sp_oral_taxon_178
Oenococcus_oeni
Oenococcus_kitaharae
Leuconostoc_fallax
Leuconostoc_pseudomesenteroides
Leuconostoc_carnosum
Leuconostoc_pseudomesenteroides
Leuconostoc_lactis
Leuconostoc_gelidum_subsp_gasicomitatum_LMG_18811
Leuconostoc
Leuconostoc_gelidum
Leuconostoc_gelidum
Leuconostoc_kimchii_IMSNU_11154
Leuconostoc_sp_C2
Leuconostoc
Leuconostoc_mesenteroides
Leuconostoc_mesenteroides_subsp_mesenteroides_ATCC_8293
Leuconostoc_citreum
Leuconostoc_sp_DORA_2
Weissella_oryzae
Weissella_confusa
Weissella_cibaria
Weissella_thailandensis_fsh4_2
Weissella_paramesenteroides
Wohlfahrtiimonas_chitiniclastica
Wohlfahrtiimonas_chitiniclastica

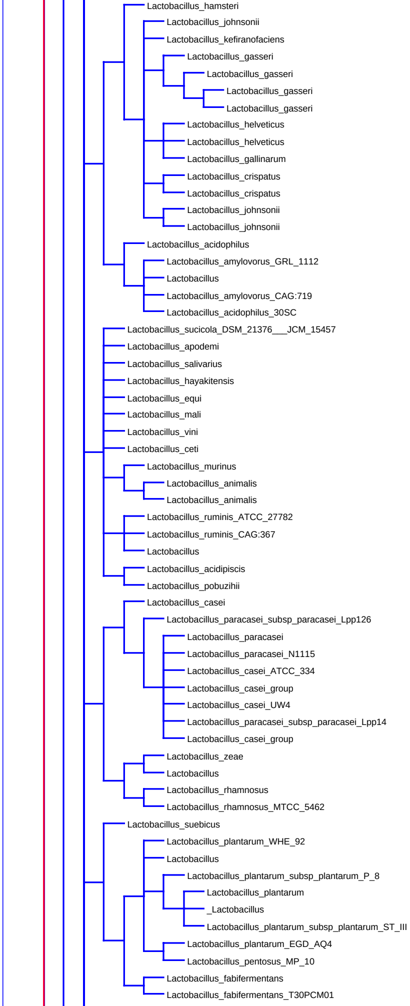
Lactobacillus_hamsteri
Lactobacillus_johnsonii
Lactobacillus_kefiranofaciens
Lactobacillus_gasseri
Lactobacillus_gasseri
Lactobacillus_gasseri
Lactobacillus_gasseri
Lactobacillus_helveticus
Lactobacillus_helveticus
Lactobacillus_gallinarum
Lactobacillus_crispatus
Lactobacillus_crispatus
Lactobacillus_johnsonii
Lactobacillus_johnsonii
Lactobacillus_acidophilus
Lactobacillus_amylovorus_GRL_1112
Lactobacillus
Lactobacillus_amylovorus_CAG:719
Lactobacillus_acidophilus_30SC
Lactobacillus_sucicola_DSM_21376___JCM_15457
Lactobacillus_apodemi
Lactobacillus_salivarius
Lactobacillus_hayakitensis
Lactobacillus_equi
Lactobacillus_mali
Lactobacillus_vini
Lactobacillus_ceti
Lactobacillus_murinus
Lactobacillus_animalis
Lactobacillus_animalis
Lactobacillus_ruminis_ATCC_27782
Lactobacillus_ruminis_CAG:367
Lactobacillus
Lactobacillus_acidipiscis
Lactobacillus_pobuzihii
Lactobacillus_casei
Lactobacillus_paracasei_subsp_paracasei_Lpp126
Lactobacillus_paracasei
Lactobacillus_paracasei_N1115
Lactobacillus_casei_ATCC_334
Lactobacillus_casei_group
Lactobacillus_casei_UW4
Lactobacillus_paracasei_subsp_paracasei_Lpp14
Lactobacillus_casei_group
Lactobacillus_zeae
Lactobacillus
Lactobacillus_rhamnosus
Lactobacillus_rhamnosus_MTCC_5462
Lactobacillus_suebicus
Lactobacillus_plantarum_WHE_92
Lactobacillus
Lactobacillus_plantarum_subsp_plantarum_P_8
Lactobacillus_plantarum
_Lactobacillus
Lactobacillus_plantarum_subsp_plantarum_ST_III
Lactobacillus_plantarum_EGD_AQ4
Lactobacillus_pentosus_MP_10
Lactobacillus_fabifermentans
Lactobacillus_fabifermentans_T30PCM01

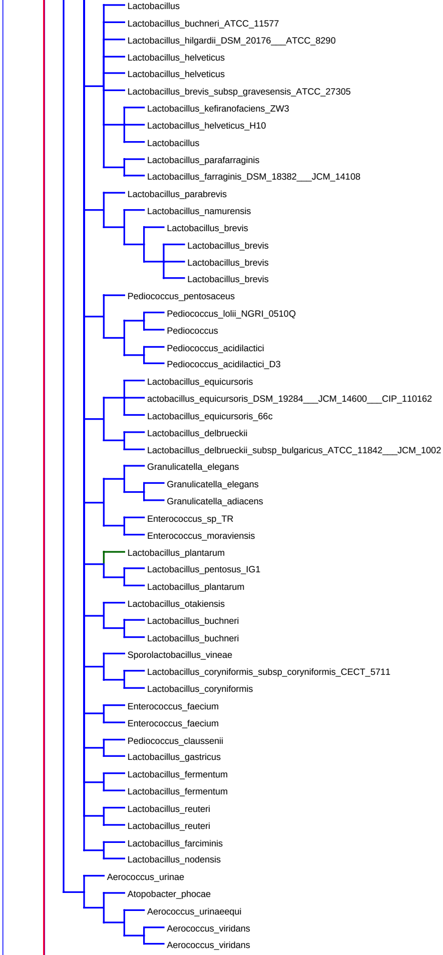
Lactobacillus
Lactobacillus_buchneri_ATCC_11577
Lactobacillus_hilgardii_DSM_20176___ATCC_8290
Lactobacillus_helveticus
Lactobacillus_helveticus
Lactobacillus_brevis_subsp_gravesensis_ATCC_27305
Lactobacillus_kefiranofaciens_ZW3
Lactobacillus_helveticus_H10
Lactobacillus
Lactobacillus_parafarraginis
Lactobacillus_farraginis_DSM_18382___JCM_14108
Lactobacillus_parabrevis
Lactobacillus_namurensis
Lactobacillus_brevis
Lactobacillus_brevis
Lactobacillus_brevis
Lactobacillus_brevis
Pediococcus_pentosaceus
Pediococcus_lolii_NGRI_0510Q
Pediococcus
Pediococcus_acidilactici
Pediococcus_acidilactici_D3
Lactobacillus_equicursoris
actobacillus_equicursoris_DSM_19284___JCM_14600___CIP_110162
Lactobacillus_equicursoris_66c
Lactobacillus_delbrueckii
Lactobacillus_delbrueckii_subsp_bulgaricus_ATCC_11842___JCM_1002
Granulicatella_elegans
Granulicatella_elegans
Granulicatella_adiacens
Enterococcus_sp_TR
Enterococcus_moraviensis
Lactobacillus_plantarum
Lactobacillus_pentosus_IG1
Lactobacillus_plantarum
Lactobacillus_otakiensis
Lactobacillus_buchneri
Lactobacillus_buchneri
Sporolactobacillus_vineae
Lactobacillus_coryniformis_subsp_coryniformis_CECT_5711
Lactobacillus_coryniformis
Enterococcus_faecium
Enterococcus_faecium
Pediococcus_claussenii
Lactobacillus_gastricus
Lactobacillus_fermentum
Lactobacillus_fermentum
Lactobacillus_reuteri
Lactobacillus_reuteri
Lactobacillus_farciminis
Lactobacillus_nodensis
Aerococcus_urinae
Atopobacter_phocae
Aerococcus_urinaeequi
Aerococcus_viridans
Aerococcus_viridans

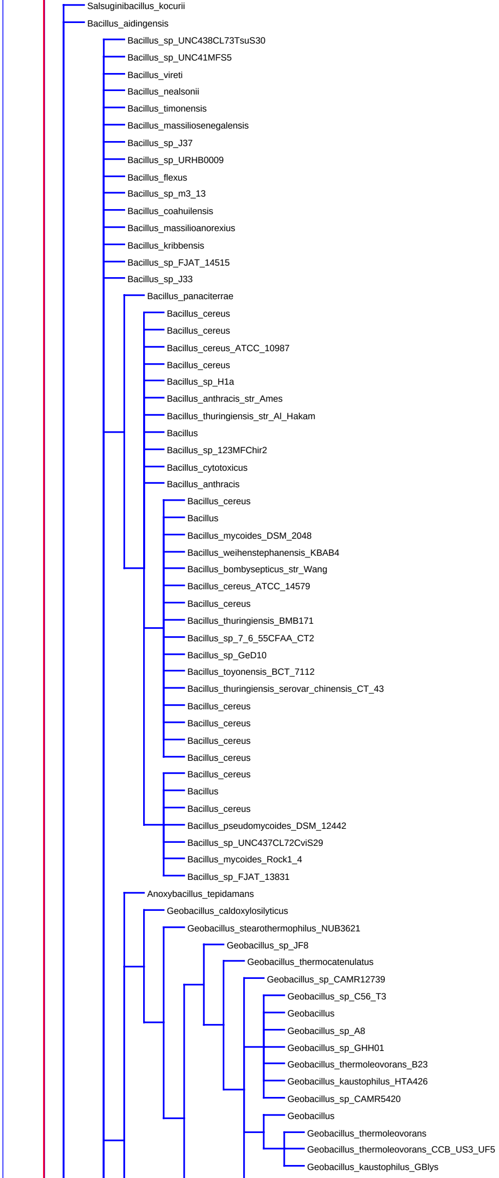
Salsuginibacillus_kocurii
Bacillus_aidingensis
Bacillus_sp_UNC438CL73TsuS30
Bacillus_sp_UNC41MFS5
Bacillus_vireti
Bacillus_nealsonii
Bacillus_timonensis
Bacillus_massiliosenegalensis
Bacillus_sp_J37
Bacillus_sp_URHB0009
Bacillus_flexus
Bacillus_sp_m3_13
Bacillus_coahuilensis
Bacillus_massilioanorexius
Bacillus_kribbensis
Bacillus_sp_FJAT_14515
Bacillus_sp_J33
Bacillus_panaciterrae
Bacillus_cereus
Bacillus_cereus
Bacillus_cereus_ATCC_10987
Bacillus_cereus
Bacillus_sp_H1a
Bacillus_anthracis_str_Ames
Bacillus_thuringiensis_str_Al_Hakam
Bacillus
Bacillus_sp_123MFChir2
Bacillus_cytotoxicus
Bacillus_anthracis
Bacillus_cereus
Bacillus
Bacillus_mycoides_DSM_2048
Bacillus_weihenstephanensis_KBAB4
Bacillus_bombysepticus_str_Wang
Bacillus_cereus_ATCC_14579
Bacillus_cereus
Bacillus_thuringiensis_BMB171
Bacillus_sp_7_6_55CFAA_CT2
Bacillus_sp_GeD10
Bacillus_toyonensis_BCT_7112
Bacillus_thuringiensis_serovar_chinensis_CT_43
Bacillus_cereus
Bacillus_cereus
Bacillus_cereus
Bacillus_cereus
Bacillus_cereus
Bacillus
Bacillus_cereus
Bacillus_pseudomycoides_DSM_12442
Bacillus_sp_UNC437CL72CviS29
Bacillus_mycoides_Rock1_4
Bacillus_sp_FJAT_13831
Anoxybacillus_tepidamans
Geobacillus_caldoxylosilyticus
Geobacillus_stearothermophilus_NUB3621
Geobacillus_sp_JF8
Geobacillus_thermocatenulatus
Geobacillus_sp_CAMR12739
Geobacillus_sp_C56_T3
Geobacillus
Geobacillus_sp_A8
Geobacillus_sp_GHH01
Geobacillus_thermoleovorans_B23
Geobacillus_kaustophilus_HTA426
Geobacillus_sp_CAMR5420
Geobacillus
Geobacillus_thermoleovorans
Geobacillus_thermoleovorans_CCB_US3_UF5
Geobacillus_kaustophilus_GBlys

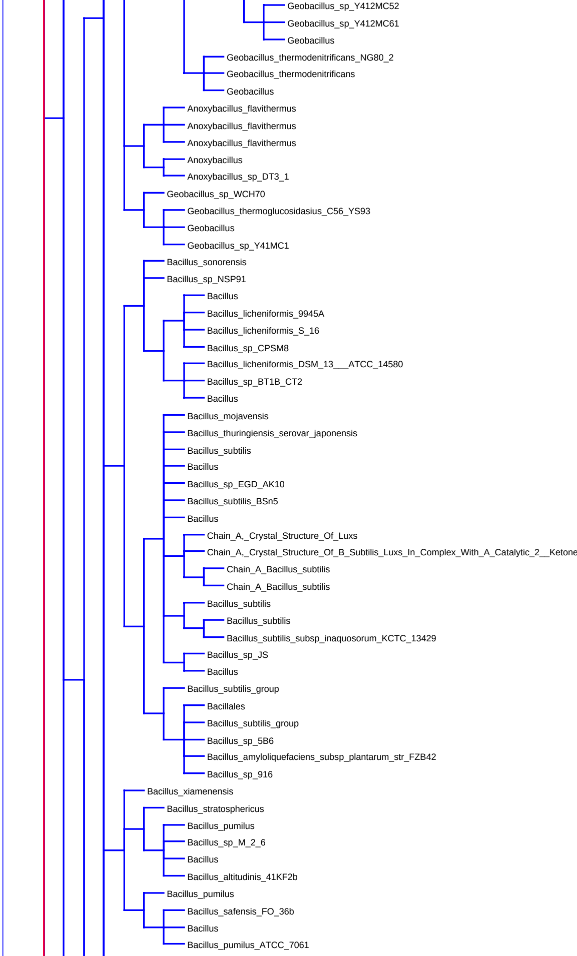
Geobacillus_sp_Y412MC52
Geobacillus_sp_Y412MC61
Geobacillus
Geobacillus_thermodenitrificans_NG80_2
Geobacillus_thermodenitrificans
Geobacillus
Anoxybacillus_flavithermus
Anoxybacillus_flavithermus
Anoxybacillus_flavithermus
Anoxybacillus
Anoxybacillus_sp_DT3_1
Geobacillus_sp_WCH70
Geobacillus_thermoglucosidasius_C56_YS93
Geobacillus
Geobacillus_sp_Y41MC1
Bacillus_sonorensis
Bacillus_sp_NSP91
Bacillus
Bacillus_licheniformis_9945A
Bacillus_licheniformis_S_16
Bacillus_sp_CPSM8
Bacillus_licheniformis_DSM_13___ATCC_14580
Bacillus_sp_BT1B_CT2
Bacillus
Bacillus_mojavensis
Bacillus_thuringiensis_serovar_japonensis
Bacillus_subtilis
Bacillus
Bacillus_sp_EGD_AK10
Bacillus_subtilis_BSn5
Bacillus
Chain_A,_Crystal_Structure_Of_Luxs
Chain_A,_Crystal_Structure_Of_B_Subtilis_Luxs_In_Complex_With_A_Catalytic_2__Ketone
Chain_A_Bacillus_subtilis
Chain_A_Bacillus_subtilis
Bacillus_subtilis
Bacillus_subtilis
Bacillus_subtilis_subsp_inaquosorum_KCTC_13429
Bacillus_sp_JS
Bacillus
Bacillus_subtilis_group
Bacillales
Bacillus_subtilis_group
Bacillus_sp_5B6
Bacillus_amyloliquefaciens_subsp_plantarum_str_FZB42
Bacillus_sp_916
Bacillus_xiamenensis
Bacillus_stratosphericus
Bacillus_pumilus
Bacillus_sp_M_2_6
Bacillus
Bacillus_altitudinis_41KF2b
Bacillus_pumilus
Bacillus_safensis_FO_36b
Bacillus
Bacillus_pumilus_ATCC_7061

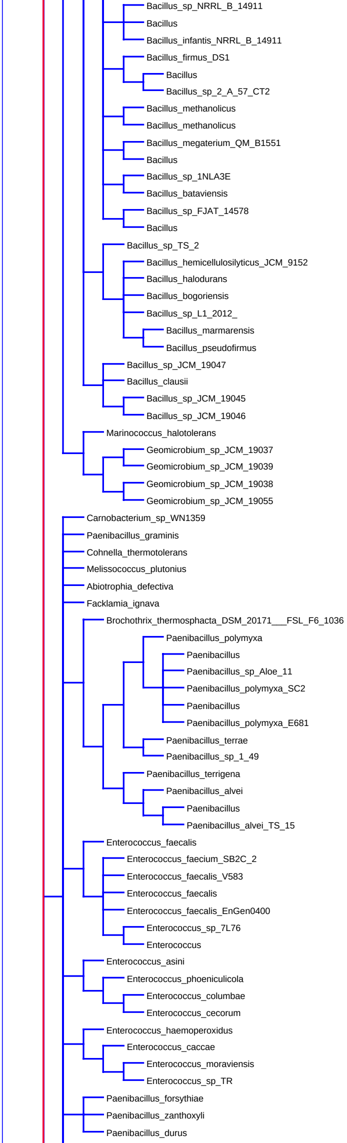
Bacillus_sp_NRRL_B_14911
Bacillus
Bacillus_infantis_NRRL_B_14911
Bacillus_firmus_DS1
Bacillus
Bacillus_sp_2_A_57_CT2
Bacillus_methanolicus
Bacillus_methanolicus
Bacillus_megaterium_QM_B1551
Bacillus
Bacillus_sp_1NLA3E
Bacillus_bataviensis
Bacillus_sp_FJAT_14578
Bacillus
Bacillus_sp_TS_2
Bacillus_hemicellulosilyticus_JCM_9152
Bacillus_halodurans
Bacillus_bogoriensis
Bacillus_sp_L1_2012_
Bacillus_marmarensis
Bacillus_pseudofirmus
Bacillus_sp_JCM_19047
Bacillus_clausii
Bacillus_sp_JCM_19045
Bacillus_sp_JCM_19046
Marinococcus_halotolerans
Geomicrobium_sp_JCM_19037
Geomicrobium_sp_JCM_19039
Geomicrobium_sp_JCM_19038
Geomicrobium_sp_JCM_19055
Carnobacterium_sp_WN1359
Paenibacillus_graminis
Cohnella_thermotolerans
Melissococcus_plutonius
Abiotrophia_defectiva
Facklamia_ignava
Brochothrix_thermosphacta_DSM_20171___FSL_F6_1036
Paenibacillus_polymyxa
Paenibacillus
Paenibacillus_sp_Aloe_11
Paenibacillus_polymyxa_SC2
Paenibacillus
Paenibacillus_polymyxa_E681
Paenibacillus_terrae
Paenibacillus_sp_1_49
Paenibacillus_terrigena
Paenibacillus_alvei
Paenibacillus
Paenibacillus_alvei_TS_15
Enterococcus_faecalis
Enterococcus_faecium_SB2C_2
Enterococcus_faecalis_V583
Enterococcus_faecalis
Enterococcus_faecalis_EnGen0400
Enterococcus_sp_7L76
Enterococcus
Enterococcus_asini
Enterococcus_phoeniculicola
Enterococcus_columbae
Enterococcus_cecorum
Enterococcus_haemoperoxidus
Enterococcus_caccae
Enterococcus_moraviensis
Enterococcus_sp_TR
Paenibacillus_forsythiae
Paenibacillus_zanthoxyli
Paenibacillus_durus

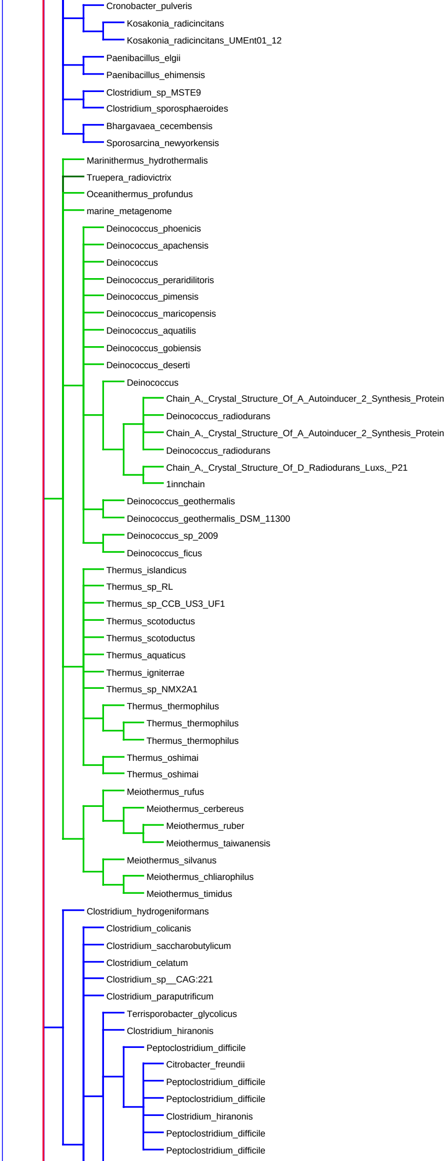
Cronobacter_pulveris
Kosakonia_radicincitans
Kosakonia_radicincitans_UMEnt01_12
Paenibacillus_elgii
Paenibacillus_ehimensis
Clostridium_sp_MSTE9
Clostridium_sporosphaeroides
Bhargavaea_cecembensis
Sporosarcina_newyorkensis
Marinithermus_hydrothermalis
Truepera_radiovictrix
Oceanithermus_profundus
marine_metagenome
Deinococcus_phoenicis
Deinococcus_apachensis
Deinococcus
Deinococcus_peraridilitoris
Deinococcus_pimensis
Deinococcus_maricopensis
Deinococcus_aquatilis
Deinococcus_gobiensis
Deinococcus_deserti
Deinococcus
Chain_A,_Crystal_Structure_Of_A_Autoinducer_2_Synthesis_Protein
Deinococcus_radiodurans
Chain_A,_Crystal_Structure_Of_A_Autoinducer_2_Synthesis_Protein
Deinococcus_radiodurans
Chain_A,_Crystal_Structure_Of_D_Radiodurans_Luxs,_P21
1innchain
Deinococcus_geothermalis
Deinococcus_geothermalis_DSM_11300
Deinococcus_sp_2009
Deinococcus_ficus
Thermus_islandicus
Thermus_sp_RL
Thermus_sp_CCB_US3_UF1
Thermus_scotoductus
Thermus_scotoductus
Thermus_aquaticus
Thermus_igniterrae
Thermus_sp_NMX2A1
Thermus_thermophilus
Thermus_thermophilus
Thermus_thermophilus
Thermus_oshimai
Thermus_oshimai
Meiothermus_rufus
Meiothermus_cerbereus
Meiothermus_ruber
Meiothermus_taiwanensis
Meiothermus_silvanus
Meiothermus_chliarophilus
Meiothermus_timidus
Clostridium_hydrogeniformans
Clostridium_colicanis
Clostridium_saccharobutylicum
Clostridium_celatum
Clostridium_sp__CAG:221
Clostridium_paraputrificum
Terrisporobacter_glycolicus
Clostridium_hiranonis
Peptoclostridium_difficile
Citrobacter_freundii
Peptoclostridium_difficile
Peptoclostridium_difficile
Clostridium_hiranonis
Peptoclostridium_difficile
Peptoclostridium_difficile

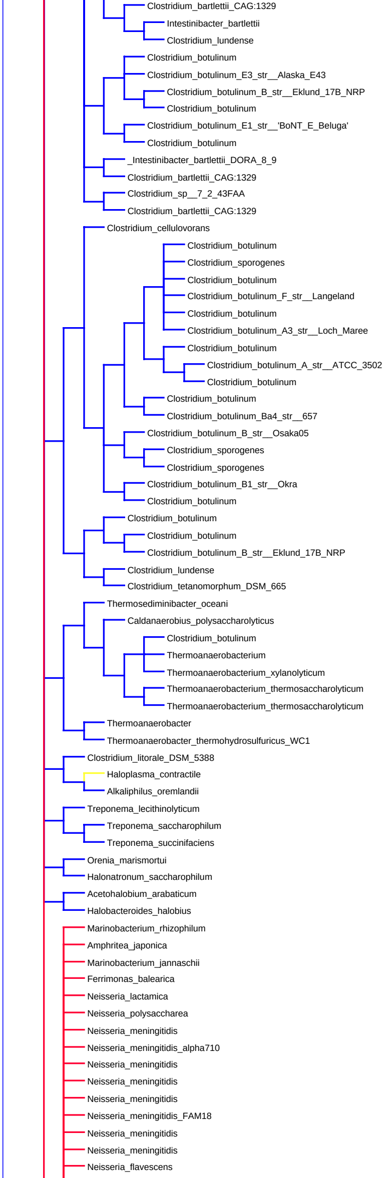
Clostridium_bartlettii_CAG:1329
Intestinibacter_bartlettii
Clostridium_lundense
Clostridium_botulinum
Clostridium_botulinum_E3_str__Alaska_E43
Clostridium_botulinum_B_str__Eklund_17B_NRP
Clostridium_botulinum
Clostridium_botulinum_E1_str__'BoNT_E_Beluga'
Clostridium_botulinum
_Intestinibacter_bartlettii_DORA_8_9
Clostridium_bartlettii_CAG:1329
Clostridium_sp__7_2_43FAA
Clostridium_bartlettii_CAG:1329
Clostridium_cellulovorans
Clostridium_botulinum
Clostridium_sporogenes
Clostridium_botulinum
Clostridium_botulinum_F_str__Langeland
Clostridium_botulinum
Clostridium_botulinum_A3_str__Loch_Maree
Clostridium_botulinum
Clostridium_botulinum_A_str__ATCC_3502
Clostridium_botulinum
Clostridium_botulinum
Clostridium_botulinum_Ba4_str__657
Clostridium_botulinum_B_str__Osaka05
Clostridium_sporogenes
Clostridium_sporogenes
Clostridium_botulinum_B1_str__Okra
Clostridium_botulinum
Clostridium_botulinum
Clostridium_botulinum
Clostridium_botulinum_B_str__Eklund_17B_NRP
Clostridium_lundense
Clostridium_tetanomorphum_DSM_665
Thermosediminibacter_oceani
Caldanaerobius_polysaccharolyticus
Clostridium_botulinum
Thermoanaerobacterium
Thermoanaerobacterium_xylanolyticum
Thermoanaerobacterium_thermosaccharolyticum
Thermoanaerobacterium_thermosaccharolyticum
Thermoanaerobacter
Thermoanaerobacter_thermohydrosulfuricus_WC1
Clostridium_litorale_DSM_5388
Haloplasma_contractile
Alkaliphilus_oremlandii
Treponema_lecithinolyticum
Treponema_saccharophilum
Treponema_succinifaciens
Orenia_marismortui
Halonatronum_saccharophilum
Acetohalobium_arabaticum
Halobacteroides_halobius
Marinobacterium_rhizophilum
Amphritea_japonica
Marinobacterium_jannaschii
Ferrimonas_balearica
Neisseria_lactamica
Neisseria_polysaccharea
Neisseria_meningitidis
Neisseria_meningitidis_alpha710
Neisseria_meningitidis
Neisseria_meningitidis
Neisseria_meningitidis
Neisseria_meningitidis_FAM18
Neisseria_meningitidis
Neisseria_meningitidis
Neisseria_flavescens

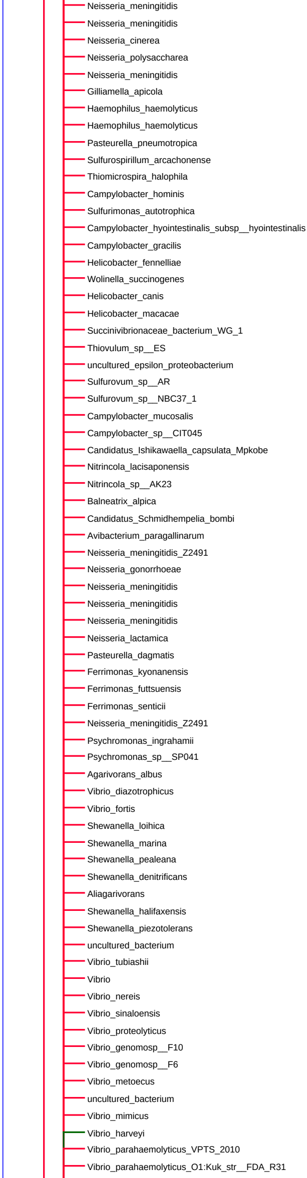
Neisseria_meningitidis
Neisseria_meningitidis
Neisseria_cinerea
Neisseria_polysaccharea
Neisseria_meningitidis
Gilliamella_apicola
Haemophilus_haemolyticus
Haemophilus_haemolyticus
Pasteurella_pneumotropica
Sulfurospirillum_arcachonense
Thiomicrospira_halophila
Campylobacter_hominis
Sulfurimonas_autotrophica
Campylobacter_hyointestinalis_subsp__hyointestinalis
Campylobacter_gracilis
Helicobacter_fennelliae
Wolinella_succinogenes
Helicobacter_canis
Helicobacter_macacae
Succinivibrionaceae_bacterium_WG_1
Thiovulum_sp__ES
uncultured_epsilon_proteobacterium
Sulfurovum_sp__AR
Sulfurovum_sp__NBC37_1
Campylobacter_mucosalis
Campylobacter_sp__CIT045
Candidatus_Ishikawaella_capsulata_Mpkobe
Nitrincola_lacisaponensis
Nitrincola_sp__AK23
Balneatrix_alpica
Candidatus_Schmidhempelia_bombi
Avibacterium_paragallinarum
Neisseria_meningitidis_Z2491
Neisseria_gonorrhoeae
Neisseria_meningitidis
Neisseria_meningitidis
Neisseria_meningitidis
Neisseria_lactamica
Pasteurella_dagmatis
Ferrimonas_kyonanensis
Ferrimonas_futtsuensis
Ferrimonas_senticii
Neisseria_meningitidis_Z2491
Psychromonas_ingrahamii
Psychromonas_sp__SP041
Agarivorans_albus
Vibrio_diazotrophicus
Vibrio_fortis
Shewanella_loihica
Shewanella_marina
Shewanella_pealeana
Shewanella_denitrificans
Aliagarivorans
Shewanella_halifaxensis
Shewanella_piezotolerans
uncultured_bacterium
Vibrio_tubiashii
Vibrio
Vibrio_nereis
Vibrio_sinaloensis
Vibrio_proteolyticus
Vibrio_genomosp__F10
Vibrio_genomosp__F6
Vibrio_metoecus
uncultured_bacterium
Vibrio_mimicus
Vibrio_harveyi
Vibrio_parahaemolyticus_VPTS_2010
Vibrio_parahaemolyticus_O1:Kuk_str__FDA_R31

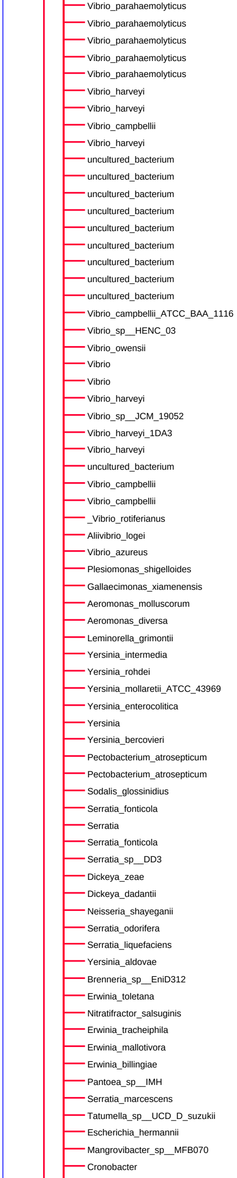
Vibrio_parahaemolyticus
Vibrio_parahaemolyticus
Vibrio_parahaemolyticus
Vibrio_parahaemolyticus
Vibrio_parahaemolyticus
Vibrio_harveyi
Vibrio_harveyi
Vibrio_campbellii
Vibrio_harveyi
uncultured_bacterium
uncultured_bacterium
uncultured_bacterium
uncultured_bacterium
uncultured_bacterium
uncultured_bacterium
uncultured_bacterium
uncultured_bacterium
uncultured_bacterium
Vibrio_campbellii_ATCC_BAA_1116
Vibrio_sp__HENC_03
Vibrio_owensii
Vibrio
Vibrio
Vibrio_harveyi
Vibrio_sp__JCM_19052
Vibrio_harveyi_1DA3
Vibrio_harveyi
uncultured_bacterium
Vibrio_campbellii
Vibrio_campbellii
_Vibrio_rotiferianus
Aliivibrio_logei
Vibrio_azureus
Plesiomonas_shigelloides
Gallaecimonas_xiamenensis
Aeromonas_molluscorum
Aeromonas_diversa
Leminorella_grimontii
Yersinia_intermedia
Yersinia_rohdei
Yersinia_mollaretii_ATCC_43969
Yersinia_enterocolitica
Yersinia
Yersinia_bercovieri
Pectobacterium_atrosepticum
Pectobacterium_atrosepticum
Sodalis_glossinidius
Serratia_fonticola
Serratia
Serratia_fonticola
Serratia_sp__DD3
Dickeya_zeae
Dickeya_dadantii
Neisseria_shayeganii
Serratia_odorifera
Serratia_liquefaciens
Yersinia_aldovae
Brenneria_sp__EniD312
Erwinia_toletana
Nitratifractor_salsuginis
Erwinia_tracheiphila
Erwinia_mallotivora
Erwinia_billingiae
Pantoea_sp__IMH
Serratia_marcescens
Tatumella_sp__UCD_D_suzukii
Escherichia_hermannii
Mangrovibacter_sp__MFB070
Cronobacter

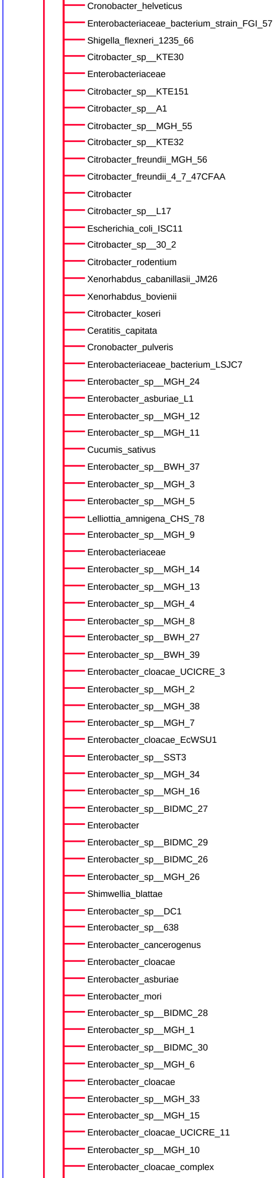
Cronobacter_helveticus
Enterobacteriaceae_bacterium_strain_FGI_57
Shigella_flexneri_1235_66
Citrobacter_sp__KTE30
Enterobacteriaceae
Citrobacter_sp__KTE151
Citrobacter_sp__A1
Citrobacter_sp__MGH_55
Citrobacter_sp__KTE32
Citrobacter_freundii_MGH_56
Citrobacter_freundii_4_7_47CFAA
Citrobacter
Citrobacter_sp__L17
Escherichia_coli_ISC11
Citrobacter_sp__30_2
Citrobacter_rodentium
Xenorhabdus_cabanillasii_JM26
Xenorhabdus_bovienii
Citrobacter_koseri
Ceratitis_capitata
Cronobacter_pulveris
Enterobacteriaceae_bacterium_LSJC7
Enterobacter_sp__MGH_24
Enterobacter_asburiae_L1
Enterobacter_sp__MGH_12
Enterobacter_sp__MGH_11
Cucumis_sativus
Enterobacter_sp__BWH_37
Enterobacter_sp__MGH_3
Enterobacter_sp__MGH_5
Lelliottia_amnigena_CHS_78
Enterobacter_sp__MGH_9
Enterobacteriaceae
Enterobacter_sp__MGH_14
Enterobacter_sp__MGH_13
Enterobacter_sp__MGH_4
Enterobacter_sp__MGH_8
Enterobacter_sp__BWH_27
Enterobacter_sp__BWH_39
Enterobacter_cloacae_UCICRE_3
Enterobacter_sp__MGH_2
Enterobacter_sp__MGH_38
Enterobacter_sp__MGH_7
Enterobacter_cloacae_EcWSU1
Enterobacter_sp__SST3
Enterobacter_sp__MGH_34
Enterobacter_sp__MGH_16
Enterobacter_sp__BIDMC_27
Enterobacter
Enterobacter_sp__BIDMC_29
Enterobacter_sp__BIDMC_26
Enterobacter_sp__MGH_26
Shimwellia_blattae
Enterobacter_sp__DC1
Enterobacter_sp__638
Enterobacter_cancerogenus
Enterobacter_cloacae
Enterobacter_asburiae
Enterobacter_mori
Enterobacter_sp__BIDMC_28
Enterobacter_sp__MGH_1
Enterobacter_sp__BIDMC_30
Enterobacter_sp__MGH_6
Enterobacter_cloacae
Enterobacter_sp__MGH_33
Enterobacter_sp__MGH_15
Enterobacter_cloacae_UCICRE_11
Enterobacter_sp__MGH_10
Enterobacter_cloacae_complex

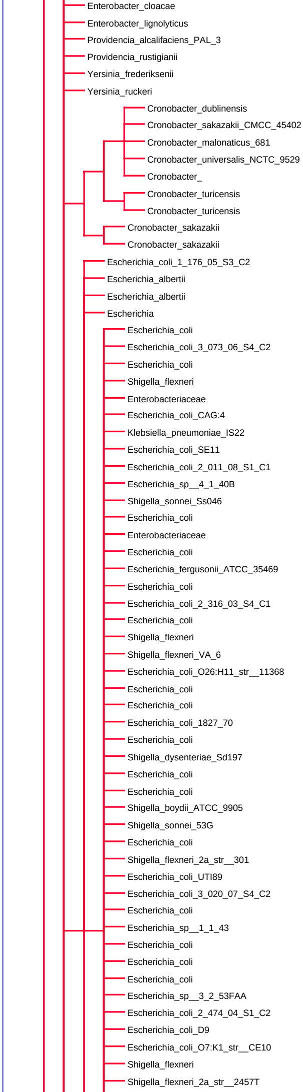
Enterobacter_cloacae
Enterobacter_lignolyticus
Providencia_alcalifaciens_PAL_3
Providencia_rustigianii
Yersinia_frederiksenii
Yersinia_ruckeri
Cronobacter_dublinensis
Cronobacter_sakazakii_CMCC_45402
Cronobacter_malonaticus_681
Cronobacter_universalis_NCTC_9529
Cronobacter_
Cronobacter_turicensis
Cronobacter_turicensis
Cronobacter_sakazakii
Cronobacter_sakazakii
Escherichia_coli_1_176_05_S3_C2
Escherichia_albertii
Escherichia_albertii
Escherichia
Escherichia_coli
Escherichia_coli_3_073_06_S4_C2
Escherichia_coli
Shigella_flexneri
Enterobacteriaceae
Escherichia_coli_CAG:4
Klebsiella_pneumoniae_IS22
Escherichia_coli_SE11
Escherichia_coli_2_011_08_S1_C1
Escherichia_sp__4_1_40B
Shigella_sonnei_Ss046
Escherichia_coli
Enterobacteriaceae
Escherichia_coli
Escherichia_fergusonii_ATCC_35469
Escherichia_coli
Escherichia_coli_2_316_03_S4_C1
Escherichia_coli
Shigella_flexneri
Shigella_flexneri_VA_6
Escherichia_coli_O26:H11_str__11368
Escherichia_coli
Escherichia_coli
Escherichia_coli_1827_70
Escherichia_coli
Shigella_dysenteriae_Sd197
Escherichia_coli
Escherichia_coli
Shigella_boydii_ATCC_9905
Shigella_sonnei_53G
Escherichia_coli
Shigella_flexneri_2a_str__301
Escherichia_coli_UTI89
Escherichia_coli_3_020_07_S4_C2
Escherichia_coli
Escherichia_sp__1_1_43
Escherichia_coli
Escherichia_coli
Escherichia_coli
Escherichia_sp__3_2_53FAA
Escherichia_coli_2_474_04_S1_C2
Escherichia_coli_D9
Escherichia_coli_O7:K1_str__CE10
Shigella_flexneri
Shigella_flexneri_2a_str__2457T

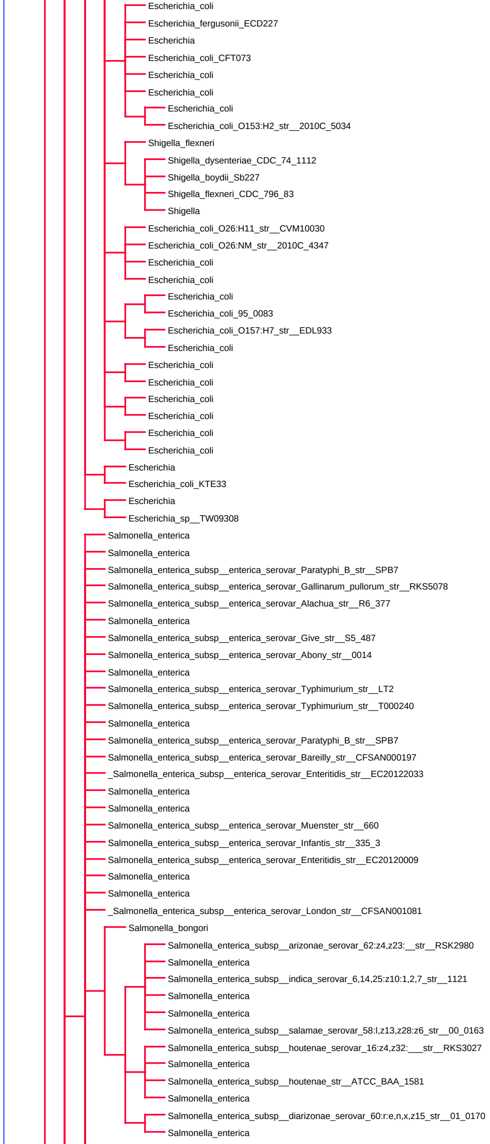
Escherichia_coli
Escherichia_fergusonii_ECD227
Escherichia
Escherichia_coli_CFT073
Escherichia_coli
Escherichia_coli
Escherichia_coli
Escherichia_coli_O153:H2_str__2010C_5034
Shigella_flexneri
Shigella_dysenteriae_CDC_74_1112
Shigella_boydii_Sb227
Shigella_flexneri_CDC_796_83
Shigella
Escherichia_coli_O26:H11_str__CVM10030
Escherichia_coli_O26:NM_str__2010C_4347
Escherichia_coli
Escherichia_coli
Escherichia_coli
Escherichia_coli_95_0083
Escherichia_coli_O157:H7_str__EDL933
Escherichia_coli
Escherichia_coli
Escherichia_coli
Escherichia_coli
Escherichia_coli
Escherichia_coli
Escherichia_coli
Escherichia
Escherichia_coli_KTE33
Escherichia
Escherichia_sp__TW09308
Salmonella_enterica
Salmonella_enterica
Salmonella_enterica_subsp__enterica_serovar_Paratyphi_B_str__SPB7
Salmonella_enterica_subsp__enterica_serovar_Gallinarum_pullorum_str__RKS5078
Salmonella_enterica_subsp__enterica_serovar_Alachua_str__R6_377
Salmonella_enterica
Salmonella_enterica_subsp__enterica_serovar_Give_str__S5_487
Salmonella_enterica_subsp__enterica_serovar_Abony_str__0014
Salmonella_enterica
Salmonella_enterica_subsp__enterica_serovar_Typhimurium_str__LT2
Salmonella_enterica_subsp__enterica_serovar_Typhimurium_str__T000240
Salmonella_enterica
Salmonella_enterica_subsp__enterica_serovar_Paratyphi_B_str__SPB7
Salmonella_enterica_subsp__enterica_serovar_Bareilly_str__CFSAN000197
_Salmonella_enterica_subsp__enterica_serovar_Enteritidis_str__EC20122033
Salmonella_enterica
Salmonella_enterica
Salmonella_enterica_subsp__enterica_serovar_Muenster_str__660
Salmonella_enterica_subsp__enterica_serovar_Infantis_str__335_3
Salmonella_enterica_subsp__enterica_serovar_Enteritidis_str__EC20120009
Salmonella_enterica
Salmonella_enterica
_Salmonella_enterica_subsp__enterica_serovar_London_str__CFSAN001081
Salmonella_bongori
Salmonella_enterica_subsp__arizonae_serovar_62:z4,z23:__str__RSK2980
Salmonella_enterica
Salmonella_enterica_subsp__indica_serovar_6,14,25:z10:1,2,7_str__1121
Salmonella_enterica
Salmonella_enterica
Salmonella_enterica_subsp__salamae_serovar_58:l,z13,z28:z6_str__00_0163
Salmonella_enterica_subsp__houtenae_serovar_16:z4,z32:___str__RKS3027
Salmonella_enterica
Salmonella_enterica_subsp__houtenae_str__ATCC_BAA_1581
Salmonella_enterica
Salmonella_enterica_subsp__diarizonae_serovar_60:r:e,n,x,z15_str__01_0170
Salmonella_enterica

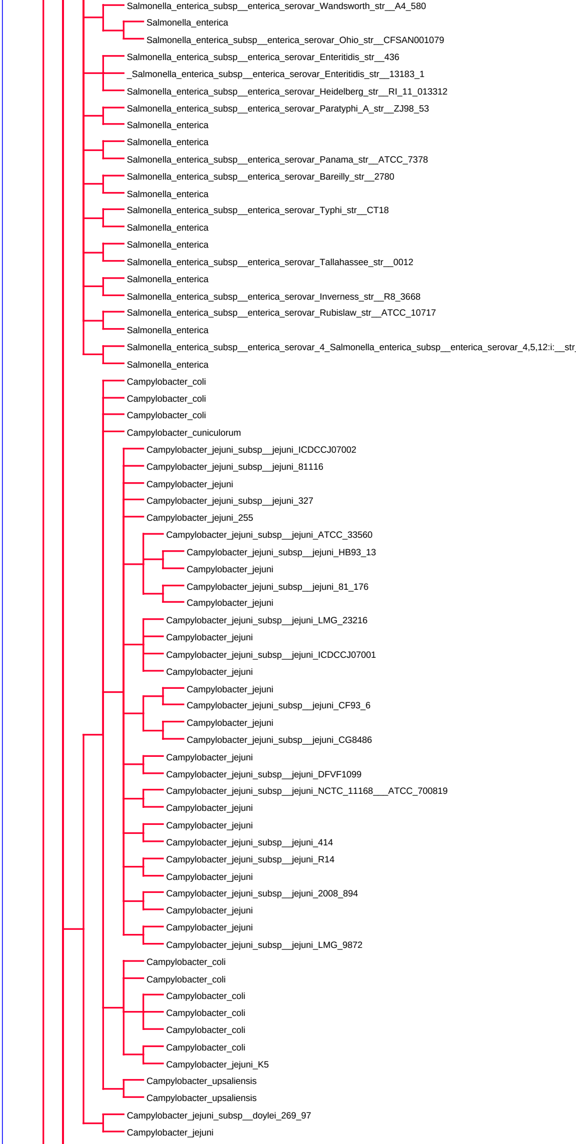
Salmonella_enterica_subsp__enterica_serovar_Wandsworth_str__A4_580
Salmonella_enterica
Salmonella_enterica_subsp__enterica_serovar_Ohio_str__CFSAN001079
Salmonella_enterica_subsp__enterica_serovar_Enteritidis_str__436
_Salmonella_enterica_subsp__enterica_serovar_Enteritidis_str__13183_1
Salmonella_enterica_subsp__enterica_serovar_Heidelberg_str__RI_11_013312
Salmonella_enterica_subsp__enterica_serovar_Paratyphi_A_str__ZJ98_53
Salmonella_enterica
Salmonella_enterica
Salmonella_enterica_subsp__enterica_serovar_Panama_str__ATCC_7378
Salmonella_enterica_subsp__enterica_serovar_Bareilly_str__2780
Salmonella_enterica
Salmonella_enterica_subsp__enterica_serovar_Typhi_str__CT18
Salmonella_enterica
Salmonella_enterica
Salmonella_enterica_subsp__enterica_serovar_Tallahassee_str__0012
Salmonella_enterica
Salmonella_enterica_subsp__enterica_serovar_Inverness_str__R8_3668
Salmonella_enterica_subsp__enterica_serovar_Rubislaw_str__ATCC_10717
Salmonella_enterica
Salmonella_enterica_subsp__enterica_serovar_4_Salmonella_enterica_subsp__enterica_serovar_4,5,12:i:__str
Salmonella_enterica
Campylobacter_coli
Campylobacter_coli
Campylobacter_coli
Campylobacter_cuniculorum
Campylobacter_jejuni_subsp__jejuni_ICDCCJ07002
Campylobacter_jejuni_subsp__jejuni_81116
Campylobacter_jejuni
Campylobacter_jejuni_subsp__jejuni_327
Campylobacter_jejuni_255
Campylobacter_jejuni_subsp__jejuni_ATCC_33560
Campylobacter_jejuni_subsp__jejuni_HB93_13
Campylobacter_jejuni
Campylobacter_jejuni_subsp__jejuni_81_176
Campylobacter_jejuni
Campylobacter_jejuni_subsp__jejuni_LMG_23216
Campylobacter_jejuni
Campylobacter_jejuni_subsp__jejuni_ICDCCJ07001
Campylobacter_jejuni
Campylobacter_jejuni
Campylobacter_jejuni_subsp__jejuni_CF93_6
Campylobacter_jejuni
Campylobacter_jejuni_subsp__jejuni_CG8486
Campylobacter_jejuni
Campylobacter_jejuni_subsp__jejuni_DFVF1099
Campylobacter_jejuni_subsp__jejuni_NCTC_11168___ATCC_700819
Campylobacter_jejuni
Campylobacter_jejuni
Campylobacter_jejuni_subsp__jejuni_414
Campylobacter_jejuni_subsp__jejuni_R14
Campylobacter_jejuni
Campylobacter_jejuni_subsp__jejuni_2008_894
Campylobacter_jejuni
Campylobacter_jejuni
Campylobacter_jejuni_subsp__jejuni_LMG_9872
Campylobacter_coli
Campylobacter_coli
Campylobacter_coli
Campylobacter_coli
Campylobacter_coli
Campylobacter_coli
Campylobacter_jejuni_K5
Campylobacter_upsaliensis
Campylobacter_upsaliensis
Campylobacter_jejuni_subsp__doylei_269_97
Campylobacter_jejuni

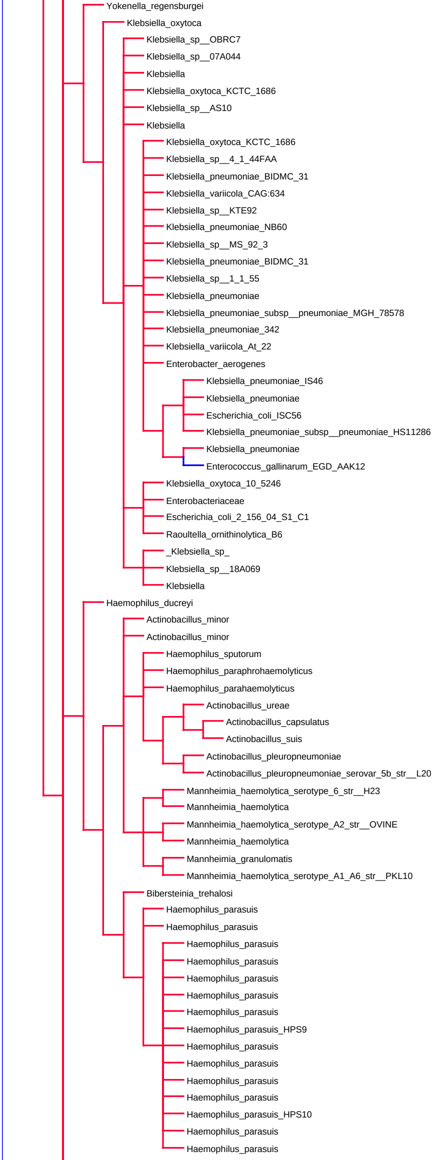
Yokenella_regensburgei
Klebsiella_oxytoca
Klebsiella_sp__OBRC7
Klebsiella_sp__07A044
Klebsiella
Klebsiella_oxytoca_KCTC_1686
Klebsiella_sp__AS10
Klebsiella
Klebsiella_oxytoca_KCTC_1686
Klebsiella_sp__4_1_44FAA
Klebsiella_pneumoniae_BIDMC_31
Klebsiella_variicola_CAG:634
Klebsiella_sp__KTE92
Klebsiella_pneumoniae_NB60
Klebsiella_sp__MS_92_3
Klebsiella_pneumoniae_BIDMC_31
Klebsiella_sp__1_1_55
Klebsiella_pneumoniae
Klebsiella_pneumoniae_subsp__pneumoniae_MGH_78578
Klebsiella_pneumoniae_342
Klebsiella_variicola_At_22
Enterobacter_aerogenes
Klebsiella_pneumoniae_IS46
Klebsiella_pneumoniae
Escherichia_coli_ISC56
Klebsiella_pneumoniae_subsp__pneumoniae_HS11286
Klebsiella_pneumoniae
Enterococcus_gallinarum_EGD_AAK12
Klebsiella_oxytoca_10_5246
Enterobacteriaceae
Escherichia_coli_2_156_04_S1_C1
Raoultella_ornithinolytica_B6
_Klebsiella_sp_
Klebsiella_sp__18A069
Klebsiella
Haemophilus_ducreyi
Actinobacillus_minor
Actinobacillus_minor
Haemophilus_sputorum
Haemophilus_paraphrohaemolyticus
Haemophilus_parahaemolyticus
Actinobacillus_ureae
Actinobacillus_capsulatus
Actinobacillus_suis
Actinobacillus_pleuropneumoniae
Actinobacillus_pleuropneumoniae_serovar_5b_str__L20
Mannheimia_haemolytica_serotype_6_str__H23
Mannheimia_haemolytica
Mannheimia_haemolytica_serotype_A2_str__OVINE
Mannheimia_haemolytica
Mannheimia_granulomatis
Mannheimia_haemolytica_serotype_A1_A6_str__PKL10
Bibersteinia_trehalosi
Haemophilus_parasuis
Haemophilus_parasuis
Haemophilus_parasuis
Haemophilus_parasuis
Haemophilus_parasuis
Haemophilus_parasuis
Haemophilus_parasuis
Haemophilus_parasuis_HPS9
Haemophilus_parasuis
Haemophilus_parasuis
Haemophilus_parasuis
Haemophilus_parasuis
Haemophilus_parasuis_HPS10
Haemophilus_parasuis
Haemophilus_parasuis

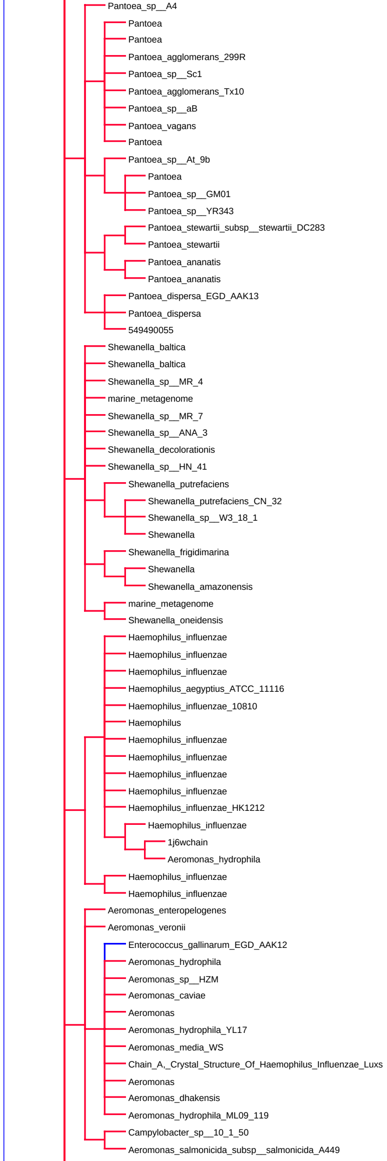
Pantoea_sp__A4
Pantoea
Pantoea
Pantoea_agglomerans_299R
Pantoea_sp__Sc1
Pantoea_agglomerans_Tx10
Pantoea_sp__aB
Pantoea_vagans
Pantoea
Pantoea_sp__At_9b
Pantoea
Pantoea_sp__GM01
Pantoea_sp__YR343
Pantoea_stewartii_subsp__stewartii_DC283
Pantoea_stewartii
Pantoea_ananatis
Pantoea_ananatis
Pantoea_dispersa_EGD_AAK13
Pantoea_dispersa
549490055
Shewanella_baltica
Shewanella_baltica
Shewanella_sp__MR_4
marine_metagenome
Shewanella_sp__MR_7
Shewanella_sp__ANA_3
Shewanella_decolorationis
Shewanella_sp__HN_41
Shewanella_putrefaciens
Shewanella_putrefaciens_CN_32
Shewanella_sp__W3_18_1
Shewanella
Shewanella_frigidimarina
Shewanella
Shewanella_amazonensis
marine_metagenome
Shewanella_oneidensis
Haemophilus_influenzae
Haemophilus_influenzae
Haemophilus_influenzae
Haemophilus_aegyptius_ATCC_11116
Haemophilus_influenzae_10810
Haemophilus
Haemophilus_influenzae
Haemophilus_influenzae
Haemophilus_influenzae
Haemophilus_influenzae
Haemophilus_influenzae_HK1212
Haemophilus_influenzae
1j6wchain
Aeromonas_hydrophila
Haemophilus_influenzae
Haemophilus_influenzae
Aeromonas_enteropelogenes
Aeromonas_veronii
Enterococcus_gallinarum_EGD_AAK12
Aeromonas_hydrophila
Aeromonas_sp__HZM
Aeromonas_caviae
Aeromonas
Aeromonas_hydrophila_YL17
Aeromonas_media_WS
Chain_A,_Crystal_Structure_Of_Haemophilus_Influenzae_Luxs
Aeromonas
Aeromonas_dhakensis
Aeromonas_hydrophila_ML09_119
Campylobacter_sp__10_1_50
Aeromonas_salmonicida_subsp__salmonicida_A449

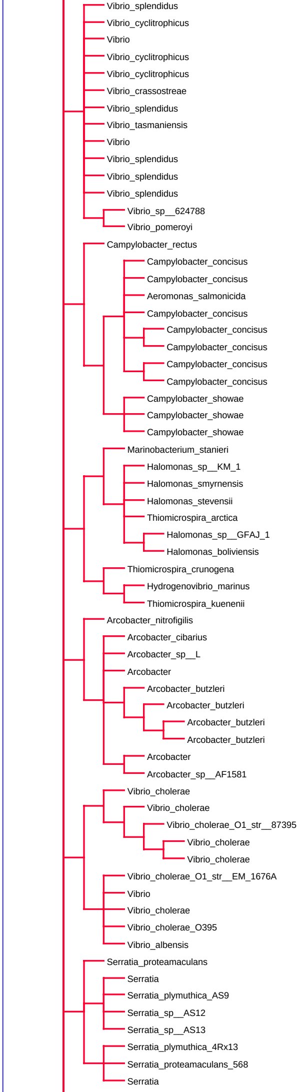
Vibrio_splendidus
Vibrio_cyclitrophicus
Vibrio
Vibrio_cyclitrophicus
Vibrio_cyclitrophicus
Vibrio_crassostreae
Vibrio_splendidus
Vibrio_tasmaniensis
Vibrio
Vibrio_splendidus
Vibrio_splendidus
Vibrio_splendidus
Vibrio_sp__624788
Vibrio_pomeroyi
Campylobacter_rectus
Campylobacter_concisus
Campylobacter_concisus
Aeromonas_salmonicida
Campylobacter_concisus
Campylobacter_concisus
Campylobacter_concisus
Campylobacter_concisus
Campylobacter_concisus
Campylobacter_showae
Campylobacter_showae
Campylobacter_showae
Marinobacterium_stanieri
Halomonas_sp__KM_1
Halomonas_smyrnensis
Halomonas_stevensii
Thiomicrospira_arctica
Halomonas_sp__GFAJ_1
Halomonas_boliviensis
Thiomicrospira_crunogena
Hydrogenovibrio_marinus
Thiomicrospira_kuenenii
Arcobacter_nitrofigilis
Arcobacter_cibarius
Arcobacter_sp__L
Arcobacter
Arcobacter_butzleri
Arcobacter_butzleri
Arcobacter_butzleri
Arcobacter_butzleri
Arcobacter
Arcobacter_sp__AF1581
Vibrio_cholerae
Vibrio_cholerae
Vibrio_cholerae_O1_str__87395
Vibrio_cholerae
Vibrio_cholerae
Vibrio_cholerae_O1_str__EM_1676A
Vibrio
Vibrio_cholerae
Vibrio_cholerae_O395
Vibrio_albensis
Serratia_proteamaculans
Serratia
Serratia_plymuthica_AS9
Serratia_sp__AS12
Serratia_sp__AS13
Serratia_plymuthica_4Rx13
Serratia_proteamaculans_568
Serratia

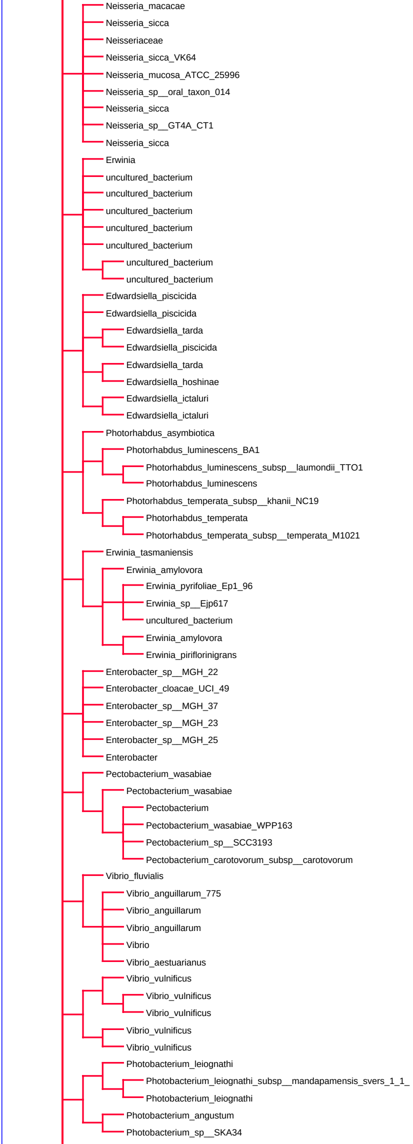
Neisseria_macacae
Neisseria_sicca
Neisseriaceae
Neisseria_sicca_VK64
Neisseria_mucosa_ATCC_25996
Neisseria_sp__oral_taxon_014
Neisseria_sicca
Neisseria_sp__GT4A_CT1
Neisseria_sicca
Erwinia
uncultured_bacterium
uncultured_bacterium
uncultured_bacterium
uncultured_bacterium
uncultured_bacterium
uncultured_bacterium
uncultured_bacterium
Edwardsiella_piscicida
Edwardsiella_piscicida
Edwardsiella_tarda
Edwardsiella_piscicida
Edwardsiella_tarda
Edwardsiella_hoshinae
Edwardsiella_ictaluri
Edwardsiella_ictaluri
Photorhabdus_asymbiotica
Photorhabdus_luminescens_BA1
Photorhabdus_luminescens_subsp__laumondii_TTO1
Photorhabdus_luminescens
Photorhabdus_temperata_subsp__khanii_NC19
Photorhabdus_temperata
Photorhabdus_temperata_subsp__temperata_M1021
Erwinia_tasmaniensis
Erwinia_amylovora
Erwinia_pyrifoliae_Ep1_96
Erwinia_sp__Ejp617
uncultured_bacterium
Erwinia_amylovora
Erwinia_piriflorinigrans
Enterobacter_sp__MGH_22
Enterobacter_cloacae_UCI_49
Enterobacter_sp__MGH_37
Enterobacter_sp__MGH_23
Enterobacter_sp__MGH_25
Enterobacter
Pectobacterium_wasabiae
Pectobacterium_wasabiae
Pectobacterium
Pectobacterium_wasabiae_WPP163
Pectobacterium_sp__SCC3193
Pectobacterium_carotovorum_subsp__carotovorum
Vibrio_fluvialis
Vibrio_anguillarum_775
Vibrio_anguillarum
Vibrio_anguillarum
Vibrio
Vibrio_aestuarianus
Vibrio_vulnificus
Vibrio_vulnificus
Vibrio_vulnificus
Vibrio_vulnificus
Vibrio_vulnificus
Photobacterium_leiognathi
Photobacterium_leiognathi_subsp__mandapamensis_svers_1_1_
Photobacterium_leiognathi
Photobacterium_angustum
Photobacterium_sp__SKA34

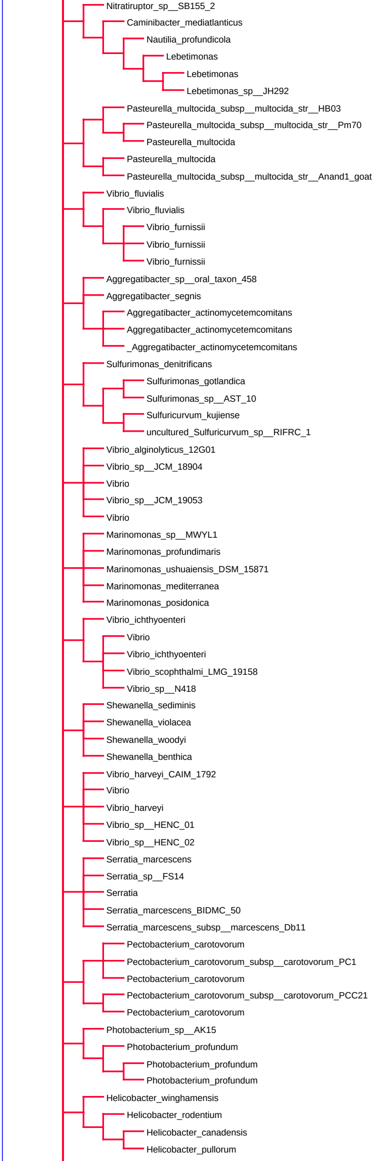
Nitratiruptor_sp__SB155_2
Caminibacter_mediatlanticus
Nautilia_profundicola
Lebetimonas
Lebetimonas
Lebetimonas_sp__JH292
Pasteurella_multocida_subsp__multocida_str__HB03
Pasteurella_multocida_subsp__multocida_str__Pm70
Pasteurella_multocida
Pasteurella_multocida
Pasteurella_multocida_subsp__multocida_str__Anand1_goat
Vibrio_fluvialis
Vibrio_fluvialis
Vibrio_furnissii
Vibrio_furnissii
Vibrio_furnissii
Aggregatibacter_sp__oral_taxon_458
Aggregatibacter_segnis
Aggregatibacter_actinomycetemcomitans
Aggregatibacter_actinomycetemcomitans
_Aggregatibacter_actinomycetemcomitans
Sulfurimonas_denitrificans
Sulfurimonas_gotlandica
Sulfurimonas_sp__AST_10
Sulfuricurvum_kujiense
uncultured_Sulfuricurvum_sp__RIFRC_1
Vibrio_alginolyticus_12G01
Vibrio_sp__JCM_18904
Vibrio
Vibrio_sp__JCM_19053
Vibrio
Marinomonas_sp__MWYL1
Marinomonas_profundimaris
Marinomonas_ushuaiensis_DSM_15871
Marinomonas_mediterranea
Marinomonas_posidonica
Vibrio_ichthyoenteri
Vibrio
Vibrio_ichthyoenteri
Vibrio_scophthalmi_LMG_19158
Vibrio_sp__N418
Shewanella_sediminis
Shewanella_violacea
Shewanella_woodyi
Shewanella_benthica
Vibrio_harveyi_CAIM_1792
Vibrio
Vibrio_harveyi
Vibrio_sp__HENC_01
Vibrio_sp__HENC_02
Serratia_marcescens
Serratia_sp__FS14
Serratia
Serratia_marcescens_BIDMC_50
Serratia_marcescens_subsp__marcescens_Db11
Pectobacterium_carotovorum
Pectobacterium_carotovorum_subsp__carotovorum_PC1
Pectobacterium_carotovorum
Pectobacterium_carotovorum_subsp__carotovorum_PCC21
Pectobacterium_carotovorum
Photobacterium_sp__AK15
Photobacterium_profundum
Photobacterium_profundum
Photobacterium_profundum
Helicobacter_winghamensis
Helicobacter_rodentium
Helicobacter_canadensis
Helicobacter_pullorum

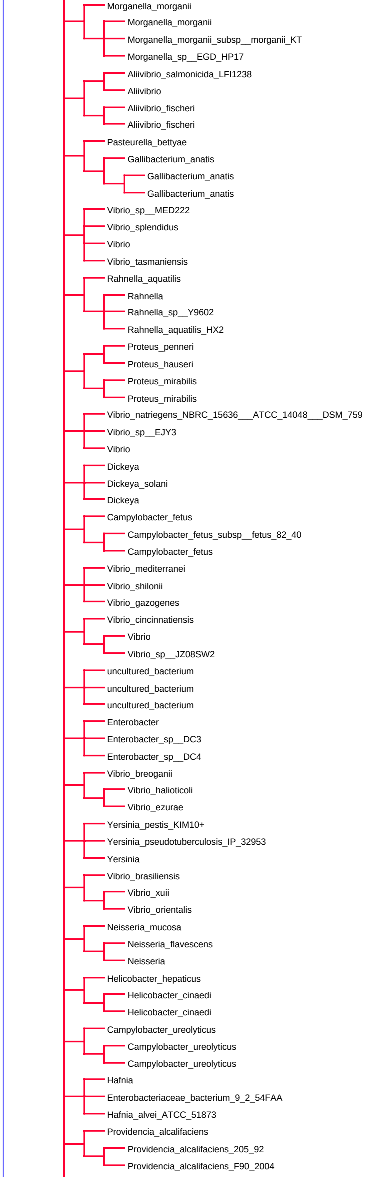
Morganella_morganii
Morganella_morganii
Morganella_morganii_subsp__morganii_KT
Morganella_sp__EGD_HP17
Aliivibrio_salmonicida_LFI1238
Aliivibrio
Aliivibrio_fischeri
Aliivibrio_fischeri
Pasteurella_bettyae
Gallibacterium_anatis
Gallibacterium_anatis
Gallibacterium_anatis
Vibrio_sp__MED222
Vibrio_splendidus
Vibrio
Vibrio_tasmaniensis
Rahnella_aquatilis
Rahnella
Rahnella_sp__Y9602
Rahnella_aquatilis_HX2
Proteus_penneri
Proteus_hauseri
Proteus_mirabilis
Proteus_mirabilis
Vibrio_natriegens_NBRC_15636___ATCC_14048___DSM_759
Vibrio_sp__EJY3
Vibrio
Dickeya
Dickeya_solani
Dickeya
Campylobacter_fetus
Campylobacter_fetus_subsp__fetus_82_40
Campylobacter_fetus
Vibrio_mediterranei
Vibrio_shilonii
Vibrio_gazogenes
Vibrio_cincinnatiensis
Vibrio
Vibrio_sp__JZ08SW2
uncultured_bacterium
uncultured_bacterium
uncultured_bacterium
Enterobacter
Enterobacter_sp__DC3
Enterobacter_sp__DC4
Vibrio_breoganii
Vibrio_halioticoli
Vibrio_ezurae
Yersinia_pestis_KIM10+
Yersinia_pseudotuberculosis_IP_32953
Yersinia
Vibrio_brasiliensis
Vibrio_xuii
Vibrio_orientalis
Neisseria_mucosa
Neisseria_flavescens
Neisseria
Helicobacter_hepaticus
Helicobacter_cinaedi
Helicobacter_cinaedi
Campylobacter_ureolyticus
Campylobacter_ureolyticus
Campylobacter_ureolyticus
Hafnia
Enterobacteriaceae_bacterium_9_2_54FAA
Hafnia_alvei_ATCC_51873
Providencia_alcalifaciens
Providencia_alcalifaciens_205_92
Providencia_alcalifaciens_F90_2004

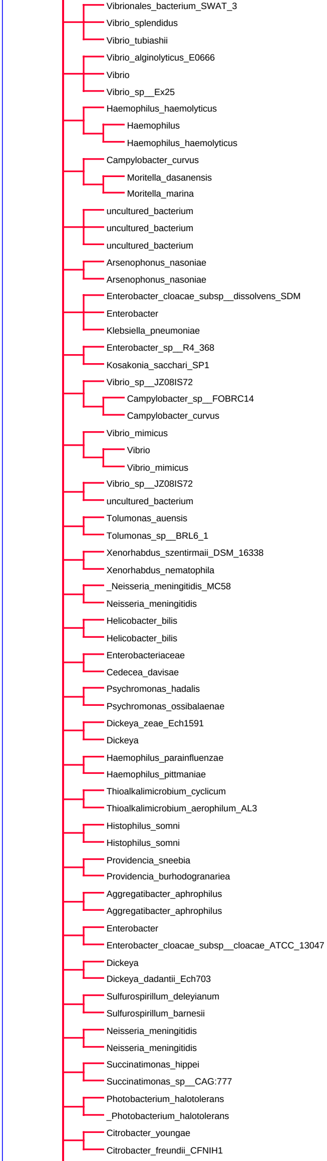
Vibrionales_bacterium_SWAT_3
Vibrio_splendidus
Vibrio_tubiashii
Vibrio_alginolyticus_E0666
Vibrio
Vibrio_sp__Ex25
Haemophilus_haemolyticus
Haemophilus
Haemophilus_haemolyticus
Campylobacter_curvus
Moritella_dasanensis
Moritella_marina
uncultured_bacterium
uncultured_bacterium
uncultured_bacterium
Arsenophonus_nasoniae
Arsenophonus_nasoniae
Enterobacter_cloacae_subsp__dissolvens_SDM
Enterobacter
Klebsiella_pneumoniae
Enterobacter_sp__R4_368
Kosakonia_sacchari_SP1
Vibrio_sp__JZ08IS72
Campylobacter_sp__FOBRC14
Campylobacter_curvus
Vibrio_mimicus
Vibrio
Vibrio_mimicus
Vibrio_sp__JZ08IS72
uncultured_bacterium
Tolumonas_auensis
Tolumonas_sp__BRL6_1
Xenorhabdus_szentirmaii_DSM_16338
Xenorhabdus_nematophila
_Neisseria_meningitidis_MC58
Neisseria_meningitidis
Helicobacter_bilis
Helicobacter_bilis
Enterobacteriaceae
Cedecea_davisae
Psychromonas_hadalis
Psychromonas_ossibalaenae
Dickeya_zeae_Ech1591
Dickeya
Haemophilus_parainfluenzae
Haemophilus_pittmaniae
Thioalkalimicrobium_cyclicum
Thioalkalimicrobium_aerophilum_AL3
Histophilus_somni
Histophilus_somni
Providencia_sneebia
Providencia_burhodogranariea
Aggregatibacter_aphrophilus
Aggregatibacter_aphrophilus
Enterobacter
Enterobacter_cloacae_subsp__cloacae_ATCC_13047
Dickeya
Dickeya_dadantii_Ech703
Sulfurospirillum_deleyianum
Sulfurospirillum_barnesii
Neisseria_meningitidis
Neisseria_meningitidis
Succinatimonas_hippei
Succinatimonas_sp__CAG:777
Photobacterium_halotolerans
_Photobacterium_halotolerans
Citrobacter_youngae
Citrobacter_freundii_CFNIH1

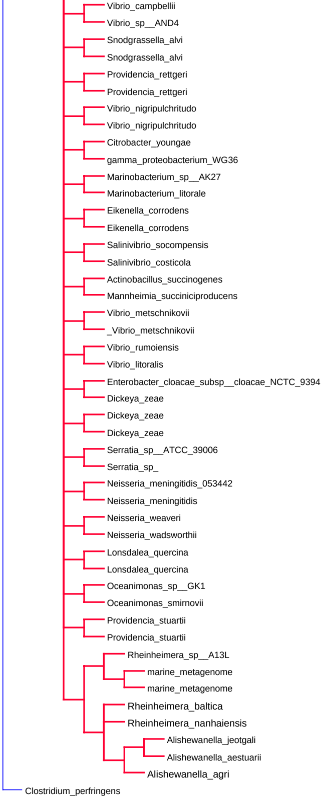
Vibrio_campbellii
Vibrio_sp__AND4
Snodgrassella_alvi
Snodgrassella_alvi
Providencia_rettgeri
Providencia_rettgeri
Vibrio_nigripulchritudo
Vibrio_nigripulchritudo
Citrobacter_youngae
gamma_proteobacterium_WG36
Marinobacterium_sp__AK27
Marinobacterium_litorale
Eikenella_corrodens
Eikenella_corrodens
Salinivibrio_socompensis
Salinivibrio_costicola
Actinobacillus_succinogenes
Mannheimia_succiniciproducens
Vibrio_metschnikovii
_Vibrio_metschnikovii
Vibrio_rumoiensis
Vibrio_litoralis
Enterobacter_cloacae_subsp__cloacae_NCTC_9394
Dickeya_zeae
Dickeya_zeae
Dickeya_zeae
Serratia_sp__ATCC_39006
Serratia_sp_
Neisseria_meningitidis_053442
Neisseria_meningitidis
Neisseria_weaveri
Neisseria_wadsworthii
Lonsdalea_quercina
Lonsdalea_quercina
Oceanimonas_sp__GK1
Oceanimonas_smirnovii
Providencia_stuartii
Providencia_stuartii
Rheinheimera_sp__A13L
marine_metagenome
marine_metagenome
Rheinheimera_baltica
Rheinheimera_nanhaiensis
Alishewanella_jeotgali
Alishewanella_aestuarii
Alishewanella_agri
Clostridium_perfringens
